# Multiplex profiling of developmental *cis-*regulatory elements with quantitative single-cell expression reporters

**DOI:** 10.1038/s41592-024-02260-3

**Published:** 2024-05-09

**Authors:** Jean-Benoît Lalanne, Samuel G. Regalado, Silvia Domcke, Diego Calderon, Beth K. Martin, Xiaoyi Li, Tony Li, Chase C. Suiter, Choli Lee, Cole Trapnell, Jay Shendure

**Affiliations:** 1 Department of Genome Sciences, University of Washington, Seattle, WA USA; 2 Molecular and Cellular Biology Program, University of Washington, Seattle, WA USA; 3 Brotman Baty Institute for Precision Medicine, Seattle, WA USA; 4 Allen Discovery Center for Cell Lineage Tracing, Seattle, WA USA; 5 Howard Hughes Medical Institute, Seattle, WA USA

**Keywords:** Gene expression, Gene expression analysis, Genomics, Differentiation

## Abstract

The inability to scalably and precisely measure the activity of developmental *cis*-regulatory elements (CREs) in multicellular systems is a bottleneck in genomics. Here we develop a dual RNA cassette that decouples the detection and quantification tasks inherent to multiplex single-cell reporter assays. The resulting measurement of reporter expression is accurate over multiple orders of magnitude, with a precision approaching the limit set by Poisson counting noise. Together with RNA barcode stabilization via circularization, these scalable single-cell quantitative expression reporters provide high-contrast readouts, analogous to classic in situ assays but entirely from sequencing. Screening >200 regions of accessible chromatin in a multicellular in vitro model of early mammalian development, we identify 13 (8 previously uncharacterized) autonomous and cell-type-specific developmental CREs. We further demonstrate that chimeric CRE pairs generate cognate two-cell-type activity profiles and assess gain- and loss-of-function multicellular expression phenotypes from CRE variants with perturbed transcription factor binding sites. Single-cell quantitative expression reporters can be applied in developmental and multicellular systems to quantitatively characterize native, perturbed and synthetic CREs at scale, with high sensitivity and at single-cell resolution.

## Abstract (web summary)

Single-cell quantitative expression reporters enable high-sensitivity quantitative characterization of *cis*-regulatory elements at the single-cell level in multicellular systems.

## Main

Developmental *cis*-regulatory elements (CREs) direct programs of gene expression that unfold with remarkable cell type and spatiotemporal specificity. This tight control underlies the emergence of form and function from a one-cell zygote. Fine-scale regulatory changes in target gene expression, caused by even single nucleotide changes, can both give rise to disease^1–3^ as well as drive evolutionary novelty^1,4^. How noncoding DNA encodes the requisite functional information remains incompletely understood even for the best-studied examples^5–8^. More broadly, biochemical marks correlated with enhancer status have now nominated >1M putative CREs in the mouse and human genomes^9^. However, functional profiling of these elements (and variants thereof) across diverse cellular states, particularly in developmental and multicellular contexts, is lagging due to the lack of scalable approaches.

In mammalian systems, most high-throughput functional studies of CREs have been performed in static contexts, typically cancer cell lines^10–13^. The scalability of these biotypes, in conjunction with massively parallel reporter assays (MPRAs)^14–16^ and related techniques^17^, has enabled the characterization of complex CRE libraries, leading to accurate sequence-to-function models^11,18–20^. However, new experimental and modeling approaches are needed to extend beyond the scalar activity of cell lines and access dynamic, multi-cell-type regimes. Scalable reporters have been used in directed mammalian differentiation models (for example, cardiac^21,22^, hematopoietic^21,23^, neuronal^22,24^ and naive to epiblast^25^) to discover developmental CREs, but these assays are usually applied to nonbranching trajectories with limited cell type heterogeneity. Until now, work on CREs in multicellular systems has predominantly been carried out with transgenic reporters assayed via in situs^26–28^, approaches that remain semi-quantitative and of limited throughput even with automation^29^. Nonetheless, even at limited scales, these studies reveal the rich phenomenology of metazoan developmental CREs, namely that kilobase-sized DNA sequences can autonomously recapitulate the complex expression patterns of their target genes even when taken out of context.

**Fig. 1:**
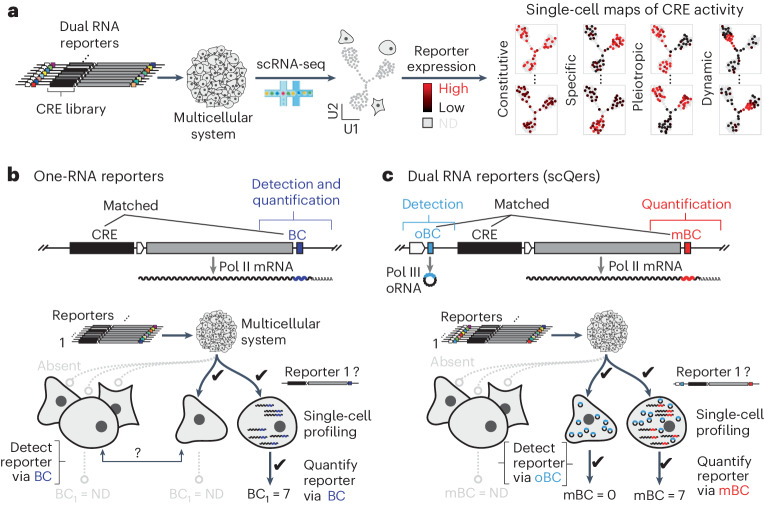
High-contrast single-cell CRE activity maps with scQers. **a**, Multiplex single-cell reporter assays. Introduction of complex libraries of integrating reporters to multicellular systems followed by scRNA-seq (U1, U2: dimensions of dimensionality reduction embedding) and computational deconvolution of reporter expression (ND: not detected). **b**, Traditional multiplex reporters harbor a single barcoded Pol II mRNA (BC, purple) driven by a library of CREs whose activity is to be profiled. In a multiplex single-cell assay, having a single transcript to both detect presence of any given reporter in a profiled cell and measure expression level is biased. In the extreme case where no mRNA is produced from a CRE in a given cell type, direct detection of the reporter is not possible (left group versus middle cell). **c**, To resolve this dropout problem, a constitutively and highly expressed Pol III-derived circularized barcoded RNA^34^ (Tornado barcodes, oBC, blue), a priori matched with the mBC (red) and CRE, is appended co-directionally upstream in a dual RNA cassette. The oBC enables robust detection of reporters in single cells, independent of reporter activity, enabling unbiased measurement of mBCs from the CRE-driven reporter mRNA.

Two recent innovations are poised to improve the throughput of mammalian regulatory biology in multicellular systems. First, stem-cell-derived models of increasing sophistication, including organoids, gastruloids and synthetic embryoids^30^, enable the scalable delivery of reporters^31^ before differentiation. Second, single-cell genomics can map cellular states and in principle be combined with multiplex reporter assays to profile CREs in multicellular models (Fig. 1a). However, in practice, multiplex reporters in single cells pose a fundamentally new challenge compared to bulk modalities: to measure the activity of any given candidate CRE, one must first determine which reporters are present in which profiled cells. As such, in porting the ‘one-RNA’ reporter strategy of traditional MPRAs directly to single-cell platforms (Fig. 1b), one relies on the barcoded messenger RNA for both (1) per-cell reporter detection and (2) quantification of expression driven by the candidate CRE. The detection task is challenging for lowly expressed reporter transcripts due to chimeric amplicons (that is, amplification products spuriously swapping barcodes originally from different molecules), which increase noise in single-cell libraries^32,33^. As such, the simplest adaptation of MPRAs to single-cell assays cannot distinguish between cells in which a given reporter is not expressed versus cells in which a given reporter is not present (Fig. 1b). This confounds the accurate quantification of reporter expression.

In this Article, to resolve this problem, we developed a dual RNA reporter that separates the detection and quantification tasks (Fig. 1c). For reporter detection, we introduce circularized^34^ RNA polymerase III (Pol III)-transcribed barcodes that enable near-complete recovery of the identity of the reporter(s) present in any given cell from single-cell RNA sequencing (scRNA-seq) data. We demonstrate that these single-cell quantitative expression reporters (scQers) are accurate over multiple orders of magnitude despite the sparsity of scRNA-seq and enable the discovery of lineage-specific regulatory elements with high sensitivity. We anticipate that scQers will enable the scalable, quantitative characterization of CREs in multicellular models of development and otherwise heterogeneous samples.

## Results

### Decoupling detection and quantification with dual reporters

We reasoned that detection and quantification could be decoupled via two separate barcoded RNAs linked on individual reporters (Fig. 1c). One barcoded RNA, highly and constitutively expressed, serves as the marker for presence/absence of the integrated reporter within any given cell. The second RNA, an RNA polymerase II (Pol II)-expressed mRNA barcoded (hereafter mBC) in its 3′ untranslated region (UTR), serves to quantify CRE activity similar to a bulk MPRA reporter. Provided that the two barcodes are a priori matched to one another, as well as to distinct CREs, one can separately detect and quantify the activity of reporters in single-cell assays.

**Extended Data Fig. 1:**
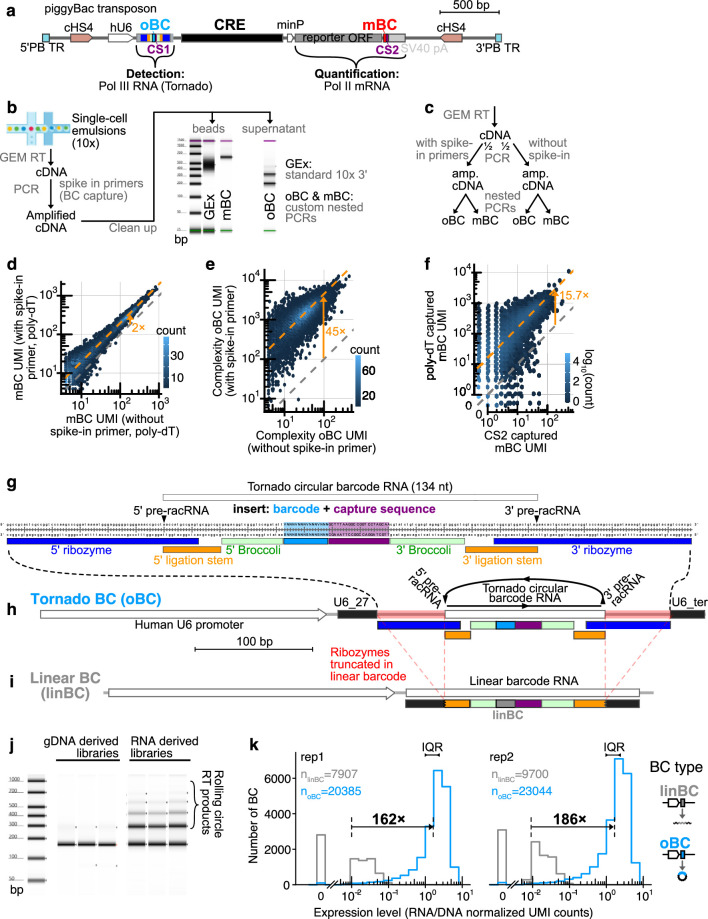
Dual RNA reporter cassette, single-cell assay, barcode capture optimization, and comparison of circularised vs. linear U6-driven barcodes. **a** At-scale schematic of the dual RNA reporter cassette in piggyBac transposon (between terminal repeats: PB TR). Flanked by convergent insulators (core chicken hypersensitive site-4 from beta-globin locus, cHS4; ref. ^48^), the human U6 (hU6) driven Tornado barcode cassette (oBC-CS1, details shown in panel **g**) is co-directionally placed upstream of the CRE library driving an open reading frame-containing reporter transcript (puromycin-P2A-GFP in the case of the promoter series in cell lines, Fig. 2a, and GFP alone for mEB experiment, Fig. 3b), barcoded in its 3′ untranslated region (mBC) upstream of an inserted capture sequence 2 (CS2), and of the SV40 polyadenylation sequence (SV40 pA). **b** Schematic of the single-cell reporter assay. After 10x Genomics (V3.1, 3′ gene expression with feature barcode) GEM reverse transcription, primers (specific to oBC and mBC RNAs) are spiked-in the cDNA amplification mix^109^. Post-cDNA amplification, in addition to standard gene expression (GEx) library generation, nested PCRs from bead fraction (mBC) and supernatant (oBC) are performed to obtain custom single-cell reporter libraries. Amplification of barcodes proceeds from different fractions as reporter mRNAs harbouring the mBC are long (>800 bp), purifying with the beads, whereas oBCs are short (134 bp), remaining in the supernatant. Example tapestation traces of resulting libraries are shown (showing laddering products from oBC libraries). **c** Experiment to assess improvement in UMI capture by spiking in primers in initial cDNA amplification. For the experiment with promoter series in cell lines (Fig. 2a), replicate B’s cDNA was split in two prior to cDNA amplification. One half, replicate B1, received spike-in primers to the oBC and mBC reporters, and the other half, replicate B2, did not. An additional round of PCR downstream of the first cDNA amplification was performed to obtain libraries in replicate B2. **d-e** Comparison of number of UMIs captured for the same cell barcode and reporter barcodes between replicates B1 (with spike-in primers) and B2 (without spike-in primers) for mBC (panel d: 2.0× median increase in UMIs captured. n = 8395 mBC-cell barcode pairs with >3 UMI) and oBC (panel e: 45× median increase in UMIs captured. n = 19323 oBC-cell barcode pairs with >3 UMI), respectively. The higher boost in capture resulting from spike-in primers for the oBC vs. mBC was likely due to the circular nature of the barcode: given the absence of 5′ end from which template switching can occur from oBC RNAs, the initial cDNA amplification (primed from the template switching oligo) effectively cannot happen except from the low abundance linear intermediates towards oBC formation; in contrast, the spike-in primers enable directly targeting sequences flanking the barcode in the circular oBC. **f** Comparison of captured mBC UMI from poly-dT vs. capture sequence 2 (CS2) on-bead reverse transcription primers (for the same mBC-cell barcode pairs). As expected from primer stoichiometry on beads, >15× increase in captured mBC UMI is seen from the poly-dT vs. CS2 primers (n = 21492 mBC-cell barcode pairs with poly-dT and CS2 mBC >0 across both replicate A and B1). CS2 thus adds marginal value for capture of the Pol II-derived polyA-tailed mBC transcripts. **g** Sequence of the Tornado system^34^ with 16 bp barcode (5′ VNNNVNNNVNNNVNNN, light blue) and downstream capture sequence 1 (CS1; burgundy) inserted in the loop of Broccoli. 5′ and 3′ (pre-racRNA) ends cleaved by ribozymes prior to circularization are highlighted (black carets). The circular product is 134 nt long. **h-i** Schematic of the human U6 promoter driven cassettes tested in a head-to-head MPRA experiments (integrated via piggyBac) to compare expression of the circular version of the barcode (Tornado barcode, or oBC, h) to the linear barcode (linear barcode, linBC, i), which is the same construct but with ‘Twister’ ribozymes removed (red highlight in h). **j** Representative tapestation (three out four libraries generated from independent biological replicates shown, two of which were sequenced and shown in panel k) traces of genomic DNA-derived vs. RNA-derived amplicon libraries prepared from the oBC vs. linBC MPRA experiment. RNA-derived libraries show clear rolling circle reverse transcription products laddering of the expected periodicity (+134 bp) expected from circular RNAs. **k** Distribution of MPRA-derived activity estimates (RNA/DNA normalised UMI) for the thousands of different, well-represented (>50 DNA UMI) barcodes of both types (hU6-driven oBC [blue] vs. hU6-driven linBC [grey]) as assessed by bulk MPRA, highlighting both the large difference in steady-state expression (>150× difference in median between linBC and oBC), and tight distribution (interquartile range <3×) for the oBC. Sub-panels correspond to two independent biological replicates. Source data

Dual RNA reporters require the contiguous production of two separate RNAs. Given that Pol II promoters can act as enhancers^35^, we expressed the detection barcode from a Pol III promoter. Interactions are expected to be minimal as a result of the largely orthogonal Pol III and Pol II machineries^36^. To avoid transcriptional collisions^37,38^, our reporter architecture (Fig. 1c and Extended Data Fig. 1a) places the hU6-driven detection barcode co-directionally upstream of the quantification cassette, which has the CRE immediately upstream of a minimal promoter (allowing for both measurement of enhancer activity and possible enhancer RNA (eRNA) production).

To mitigate the instability of short ectopic Pol III RNAs^39^, we embedded the constitutively expressed barcode within the ‘Tornado’ circularization system^34^ (Extended Data Fig. 1g,h). The resulting circular RNA barcodes, hereafter Tornado barcodes (oBC), were expressed >150-fold more highly than their linear equivalent (Extended Data Fig. 1g–k; data from genome-integrated bulk MPRA, minimal impact of random oBC sequence with ≤2.6-fold interquartile range), reaching an estimated >75,000 oBC RNA per cell per cassette^34^.

### Benchmarking with a promoter library in human cell lines

**Fig. 2:**
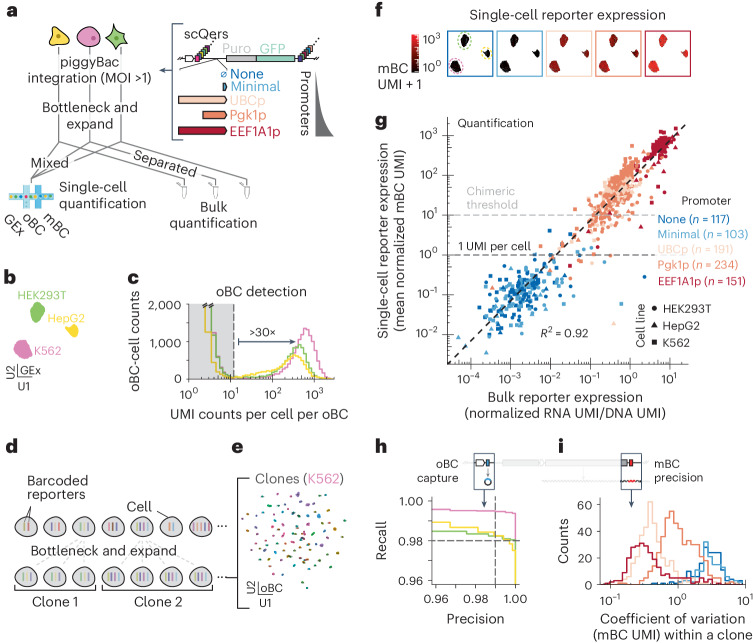
Benchmarking scQers for accuracy, precision and capture in human cell lines. **a**, An scQer library of five promoters (*n* = 1,122 unique oBC–promoter–mBC triplets, median 205 mBC–oBC pairs per promoter) was integrated in three human cell lines (HepG2, K562 and HEK293T) at high multiplicity via piggyBac. Following integration, bottlenecking and expansion, clonal cells were (1) separately subjected to bulk MPRA and (2) mixed at 1:1:1 ratio and single-cell profiled. **b**, A UMAP projection of quality-filtered single-cell transcriptomes. The three well-separated clusters correspond to the three cell lines (replicate A; cell count: K562 *n* = 2,184, HEK293T *n* = 2,090, HepG2 *n* = 1,231). **c**, The bimodal distribution of the UMI counts per oBC per cell, stratified by cell line (low count mode, truncated, gray shading: chimeric amplicons; high count modes: bona fide integrations). **d**, Clonally derived cells with a high multiplicity of reporter integrations provide internally controlled replicates of the same measurement for assessing capture of oBC and precision of mBC quantification. **e**, A UMAP projection (oBC expression space) for high-confidence-assignment cells assigned to clonotypes for K562 (replicate A; *n* = 1,430 cells, *n* = 105 clones). **f**, UMAP projection cells colored by promoter activity (average normalized mBC UMI count per cell, with pseudocount of 1). Each panel corresponds to a different promoter. **g**, Comparison between the single-cell mBC quantification (*y* axis: average normalized mBC UMI over all cells with detected matched oBC) and bulk MPRA quantification (*x* axis, RNA over DNA normalized UMI counts). Each point corresponds to an individual mBC (color: promoter, symbol: cell line). Well-represented mBCs are included (>100 bulk DNA UMI, >0 mBC single-cell UMI and ≥5 single-cell integrations). **h**, Precision–recall curves for retrieval of oBC from cells assigned to clones (consensus clonotypes taken as ground truth, aggregate over all clones with >2 cells; K562: 195 clones, 2,168 cells; HEK293T: 173 clones, 2,019 cells; HepG2: 38 clones, 1,453 cells). Dashed lines: 99% precision (1% FDR) and 98% recall (2% false negative rate, or dropout). **i**, The distribution of the coefficient of variation (mean over standard deviation) for the normalized mBC UMI counts captured measured across replicate clonal cells profiled (*n* = 946 reporters from *n* = 290 clones, across two biological replicates). Source data

**Extended Data Fig. 2:**
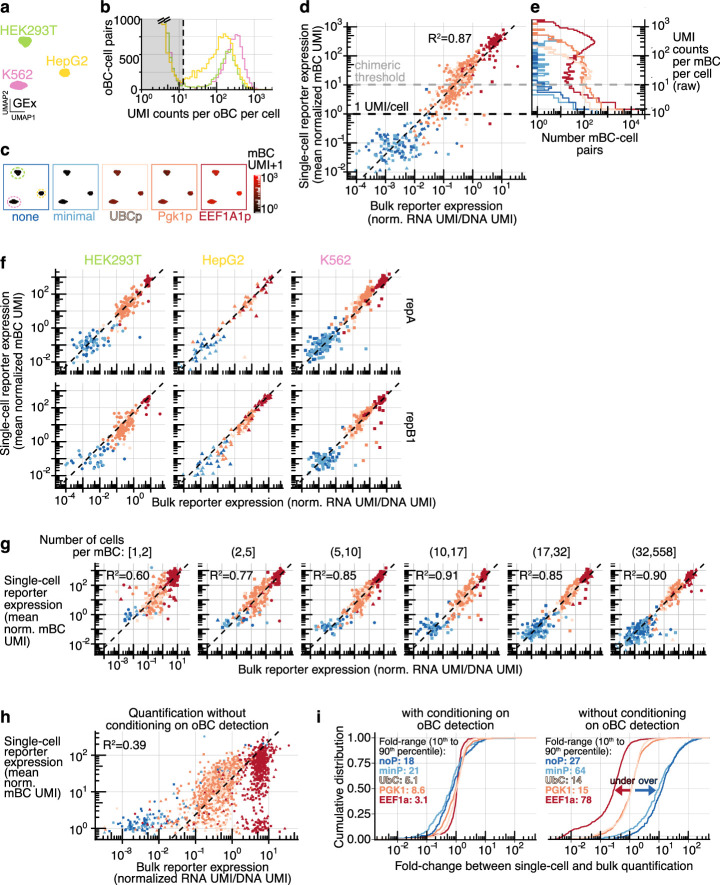
Assessment of accuracy of single-cell dual RNA reporters. **a-d** Same as Fig. 2a–c, but with data from replicate B1. **a**: Gene expression, **b**: oBC UMI count distribution, **c**: single-cell measure of reporter expression (GEx UMAP projected), **d**: comparison of bulk vs. single-cell quantification of mBC quantification. **e** Raw distribution of UMI counts per mBC per cell barcode (for valid mBC and cell barcodes pairs, not conditioning on oBC detection) stratified by associated promoter. The 10 mBC UMI/cell threshold (‘chimeric threshold’) reflects that even for highly expressed promoters, mBC UMI counts rise below that point, as a result of chimeric amplicons generated during library preparation. Without conditioning on oBC detection, these molecular species limit the dynamic range of reliable measurements with one-RNA reporters (see panel **h**). **f** Comparison of bulk MPRA quantification (x-axis, RNA over DNA normalised UMI counts) vs. single-cell quantification (y-axis: average normalised mBC UMI over all cells with detected matched oBC), same as Fig. 2g, but stratified by replicates and cell lines. Each point corresponds to an individual mBC, coloured by its associated promoter. Well-represented mBCs are included (>100 bulk DNA UMI, >0 measured mBC UMI in single cells, and ≥5 single-cell integrations detected). The diagonal dashed line follows a 1:1 slope. **g** Assessment of reporter mRNA measurement accuracy vs. number of integration events captured (both replicates). Single-cell vs. bulk quantification (same as Fig. 2g and panel **d**), but stratified by the number of cells per mBC over which the single-cell measurement is averaged (split in equal number of mBC bins). Even with as few as 5 to 10 cells captured per mBC, the correspondence with bulk measurement is on par with estimates from more highly represented mBCs (R^2^ on log-transformed values ≥ 0.85). **h** Single-cell vs. bulk quantification of mBC expression without conditioning on oBC detection (assuming all mBC capture events are valid, both replicates). In contrast to oBC conditioned measurements, quantification has a hard floor at 1 UMI/cell (slight variation around 1 from gene expression normalisation) and a limited dynamic range (y-axis spans ≈200× compared to >10^4^× with oBC conditioning, c.f., Fig. 2g and panel **d**). Only well-represented mBCs are included (same criterion as Fig. 2g: >100 DNA UMI bulk, ≥5 cells with mBC detected). Dashed line marks the 1:1 slope, highlighting systematic biases. **i** Cumulative distribution of fold-change between single-cell and bulk mBC quantification (median normalised), for both replicates, with (left) and without (right) conditional oBC detection. While the quantification conditioning on oBC is largely unbiased (centred and close to 1), quantification is biased at the high (underestimation for highly expressed EEF1A1 promoter, red arrow) and low (overestimation for low expression minimal/no promoters, blue arrow) ends of the expression spectrum. In addition to removing systematic biases, conditioning on oBC also reduces variability (quantified as the spread in fold-change, with the range spanned from 10^th^ to 90^th^ percentile for each promoter displayed on plot). Source data

The scQers cassette is defined by three components delivered to cells as a single unit: a detection oBC, a CRE and a quantification mBC. We first established that scQers report transcriptional expression in single-cells with ~2% dropout, high accuracy over a large dynamic range (<10^−1^ to >10^3^ unique molecular identifiers (UMIs) per cell), and high precision (coefficient of variation <1). To do so, we constructed a minimal library of five Pol II promoters spanning a wide activity range^40^ (Fig. 2a and Supplementary Data 1) and integrated the payloads by piggyBac^41^ transposition at high multiplicity of integration in three human cell lines (HEK293T, HepG2 and K562, median multiplicity of infection (MOI) of 4, 7 and 6, respectively). Cells were bottlenecked to a few hundred clones, expanded and then both (1) hand mixed at 1:1:1 ratios and profiled via scRNA-seq (10x Genomics 3′ feature barcoding with optimization; Extended Data Fig. 1b–f) and (2) collected separately for bulk MPRA (Fig. 2a). Thousands of cells per replicate passed standard quality filters, with cell line identity unambiguously mapped from gene expression (Fig. 2b and Extended Data Fig. 2a).

### oBCs are near-deterministically retrievable in scRNA-seq

**Extended Data Fig. 3:**
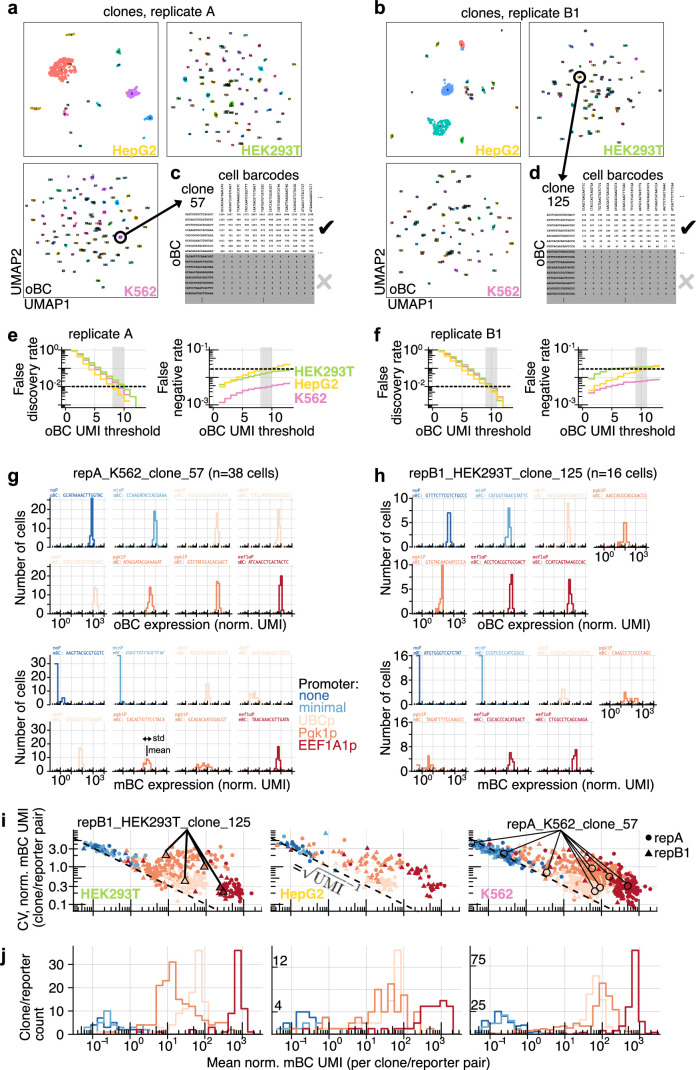
Benchmarking oBC detection and mBC capture precision with clonal analysis. **a** and **b** oBC expression space UMAP from cells assigned to high-confidence clones (coloured by mapped clone identity) with at least three cells assigned, separated by cell lines. Panel **c**: replicate A (K562: 105 clones, 1430 cells; HEK293T: 92 clones, 1330 cells; HepG2: 17 clones, 916 cells), Panel **e**: replicate B1 (K562: 90 clones, 738 cells; HEK293T: 81 clones, 689 cells; HepG2: 21 clones, 537 cells). **c** and **d** Example of raw (error corrected) UMI counts (table truncated) per cell barcode and oBC across assigned cells in clones highlighted respectively in panels **a** and **b** (oBC ordered from high to low counts). Panel **c**: clone repA_K562_clone57 with 38 cells assigned. Panel **d**: clone repB1_HEK293T_clone_125 with 16 cells assigned. Grey shading delineates oBCs not assigned to the clones, highlighting the sharp distinction in UMI counts. **e** and **f** Systematic analysis of oBC dropout across all high-confidence clones. False discovery rate (left, false positives/[true positives + false positives]), and false negative rate (right panels, false negatives/[false negatives + true positives]) as function of the oBC UMI threshold used for detection. Analyses are performed on high-confidence clones represented by at least 3 cells. Consensus reconstructed clonotypes are taken as ground truth and cells are assigned to these clonotypes with stringent threshold to remove doublets, but loose threshold to allow for up to 50% oBC dropouts per clone. At an FDR of 1% (grey shading), there are about 2% dropout (false negative rate) observed (slightly reduced performance from replicate B1 likely from halved complexity, see Extended Data Fig. 1c). Panel **e**: replicate A, Panel **f**: replicate B1. **g** and **h** Example of mBC (top) and oBC (bottom) UMI count distributions across all cells assigned to specific clones (highlighted in panels **a** and **b**). Each sub-panel corresponds to a reporter integrated in the clone. Panel **g**: clone repA_K562_clone57, with 8 integrated reporters. Panel **h**: clone repB1_HEK293T_clone_125, with 7 integrated reporters. Panels in respective positions within the oBC and mBC set are matched (for example, in repA_K562_clone57, EEF1A1 promoter with oBC:ATCAACCTCACTACTC and mBC: TAACAAACGTTGATA). **i** Coefficient of variation analysis of mBC UMI count measurements across all reporter-clone pairs stratified by cell line (left: HEK293T, middle: HepG2, right: K562). Mean over standard deviation (see panel **g** bottom: Pgk1 promoter with mBC:CACACTGTTCCTACA as schematic of both quantities) of normalised mBC UMI counts for reporters in clones as a function of mean normalised mBC UMI (reporters with >0.05 mBC UMI mean expression in clones with >4 cells assigned; replicate A: K562: 392 reporters from 83 clones, HEK293T: 198 reporters from 70 clones, HepG2: 58 reporters from 12 clones; replicate B1: K562: 213 reporters from 58 clones, HEK293T: 123 reporters from 51 clones, HepG2: 95 reporters from 14 clones). Dashed line indicates the Poisson counting scaling CV=√(UMI count)^−1^. Each point represents the quantification for a specific reporter within a clone, with point shape marking replicates and colour promoter type. As examples, reporters shown in panels **g** (clone repA_K562_clone57) and **h** (clone repB1_HEK293T_clone_125) are highlighted in black (no and minimal promoter reporters from repB1_HEK293T_clone_125 have 0 mBC UMI and therefore do not appear). **j** Assessment of position-dependent variability of integrated reporters. Panels show the distribution in mean normalised mBC UMI (expression) across reporters integrated over different clones, stratified by cell line (left: HEK293T, middle: HepG2, right: K562) and promoter type (colour). Same clone/reporter pairs as panel **i**. To account for halved library complexity in replicate B1 (see description in Extended Data Fig. 1c), reporter expression values from those clones were multiplied by two. Source data

oBCs were robustly captured on a per-cell basis. In particular, the distribution of oBC UMI counts displayed bimodality (Fig. 2c and Extended Data Fig. 2b) and >30× signal to noise. The low count mode corresponds to chimeric amplicons, and the high count mode to expression from valid integration events (~2,500 UMIs per cell per barcode, zero-truncated Poisson estimator). To assess oBC dropout, we leveraged redundant measurements across clones (Fig. 2d). Consensus integration clonotypes were identified in the bottlenecked population by relying on oBC co-detections^42,43^ (Fig. 2e, Extended Data Fig. 3a–f and Supplementary Data 2). Clonotypes served as ground truth for precision–recall analysis of detected oBCs in clone-assigned cells, revealing a false negative rate (dropout) of <2% at a false discovery rate (FDR) of 1% (Fig. 2h and Extended Data Fig. 3e–f). This represents a >10-fold improvement vis-a-vis capture of sgRNAs in single-cell clustered regularly interspaced short palindromic repeats (CRISPR) screens^43^. In sum, oBCs are transcribed barcodes that nearly eliminate dropout in scRNA-seq.

The high expression of oBCs raises the question of toxicity to cells. In line with original assessments^34^, we find little correlation between total oBC RNA expression and markers of apoptosis or immune response (for example, percent mitochondrial content *R*^2^ < 0.03, p53 expression *R*^2^ < 0.02, RIG-I expression *R*^2^ < 0.003) both in cell lines and in mEBs (experiment below).

### Accurate reporter quantification over orders of magnitude

Comparing reporter expression from single-cell and bulk quantification confirmed the accuracy of scQers. Following detection of reporter integration using oBCs (probability of multiple integrations per cell from the same oBC–promoter–mBC triplet <5%), the activity of the associated promoters can be quantified in each cell as the transcriptome-normalized average UMI counts from the matched mBC (Fig. 2f and Extended Data Fig. 2c). Single-cell averaged UMI counts across the different mBCs associated with a given promoter constituted independent measures of activity and spanned over four orders in magnitude for the five promoters (Fig. 2g and Extended Data Fig. 2d–f). Bulk MPRA measurements performed on the same cell populations were concordant across the full range of expression levels (*R*^2^ log-transformed expression ≥0.87; Fig. 2g and Extended Data Fig. 2d). Single-cell measurements of mBCs from as few as five to ten cells sufficed for accurate quantification (Extended Data Fig. 2g).

Without filtering, spurious read counts can alter reporter quantification. Indeed, library preparation requires a number of amplification steps that can generate ‘chimeric’ amplicons and lead to erroneous cell-to-barcode connections. In saturated libraries, the signature for these molecular products is a rising frequency of counts below ~10 UMIs per cell (for example, oBC: Fig. 2c, mBC: Extended Data Fig. 2e) that can result in a limit of detection substantially higher than 1 UMI per cell. A dual RNA approach does not abrogate chimeras but filters mBC reads on the basis of detection of a matched oBC in the same cell, leading to an average decrease in the tallying of chimeric counts by the proportion of cells harboring any given oBC–mBC combination. Consequently, lowly expressed mRNAs driven by the minimal and no promoter basal controls (median expression of ~0.2 UMIs per cell below the 1 UMI per cell regime inaccessible from pooled one-RNA reporters, Fig. 2g) remained accurately quantified by scQers, suggesting limited zero-inflation^44^ in our system. Leveraging our a priori matched oBC–mBC pairs, we found a high prevalence of chimeric mBC detections (mBC found in cells without a detected matched oBC: 90% EEF1A1p, 60% Pgk1p, 51% UBCp, 36% no promoter, 52% minimal promoter). As a result, quantifying activity on the basis of Pol II mBC alone (no conditioning on oBC detection) led to biases and increased variability (*R*^2^ = 0.39 for log-transformed single-cell versus bulk; 1.5- to 25-fold increased variability; Extended Data Fig. 2h,i), highlighting the quantitative advantage of dual RNA reporters.

### Measurement precision approaching Poisson counting noise

Our clonal pool of cells further allowed us to quantify variability in mBC capture. Multiply represented clones provide internal replicate measurements of the same set of reporters integrated at fixed genomic locations, controlling for an important source of variation from random integration^45–47^ (Fig. 2d). For a given reporter (mBC) integrated in a specified clone, each clonal representative sampled provides a measurement of the number of captured reporter mRNA molecules. Clones with multiple cells detected therefore enable sampling of the experimental distribution of the number of mBC UMIs per cell (Extended Data Fig. 3g,h, bottom). The variance of this distribution of mBC UMIs can then be determined, providing an estimate of the measurement precision. The minimal variance is expected to be set by Poisson counting noise, reflecting the nature of the measurement as a discrete sampling, with any additional variance corresponding to biological or technical variability. Across all reporters and clones, we find variability consistent with Poisson counting noise at low expression, and a coefficient of variation substantially below one for two of the promoters (UBCp and EEF1A1p; Fig. 2i and Extended Data Fig. 3i). The UBCp promoter in particular displayed detection close to the Poisson scaling (standard deviation/mean = 1/√mean). Variability was not strictly correlated with average expression. For example, the Pgk1p promoter, while expressed more highly than UBCp, exhibited substantially higher cell-to-cell variability (Extended Data Fig. 3i). scQers thus precisely measure reporter mRNA levels in single cells.

Systematic assessment of reporter expression across clones provided estimates of variation due to positional effects (Supplementary Note 1 and Extended Data Fig. 3j). While insulators^48^ in our construct (Extended Data Fig. 1a) substantially reduced context dependence (Supplementary Fig. 1 and Supplementary Data 3), 41–60% of mBC UMI variability in mBC UMI counts remained attributable to positional context, further confirming the technical precision of our per-cell measurement and the importance of averaging over multiple integration positions.

### Locus-level screen of putative developmental CREs

**Fig. 3:**
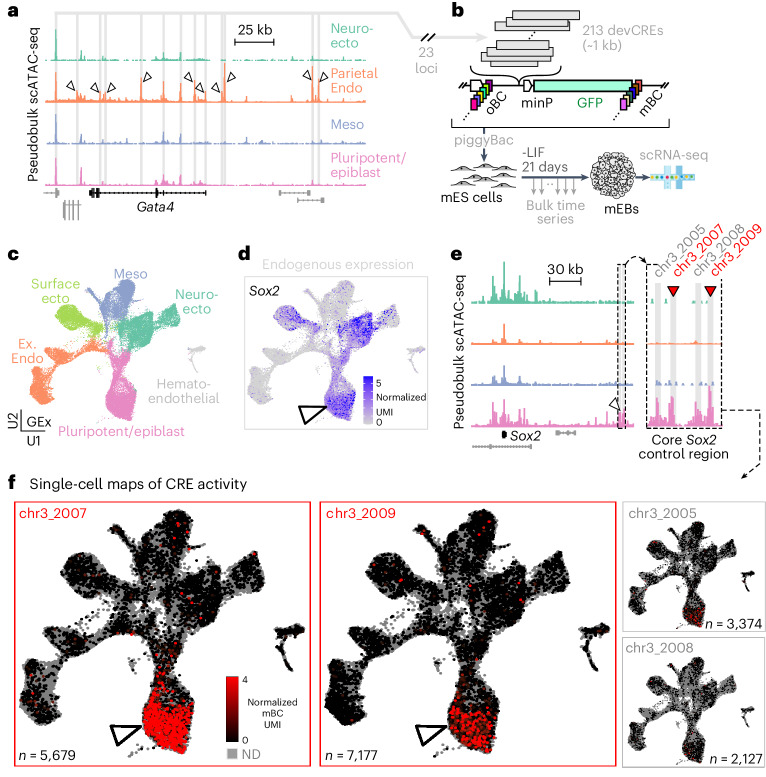
Locus-level screen of developmental CREs in mEBs. **a**, A pseudobulk pileup of scATAC-seq data at *Gata4* (±100 kb from TSS) as a representative selected developmental locus (carets indicate differentially accessible peaks). *Gata4* is expressed predominantly in parietal endoderm cells (expression Fig. 4d, top row). Reproducibly and highly accessible ATAC peaks (in expression-cognate cell type) within the 200 kb window were profiled (*n* = 13 for *Gata4*, gray shading). **b**, scQers containing 204 putative developmental CREs taken from 23 developmental loci (22 plus *Sox2* control region) were integrated at high MOI in mES cells using piggyBac. Transfected libraries included 89% CRE series, 10% exogenous promoters (same as Fig. 2a) and 1% EEF1A1p-mCherry (co-transfected for selection to increase MOI^59,60^). Reporter-integrated cells were differentiated to embryoid bodies for 21 days, with bulk sampling every 2 days, and single-cell profiling at 3 weeks. **c**, A UMAP projection of scRNA-seq (*n* = 43,799 quality-filtered cells) from three biological replicates of scQer-integrated 21-day mEB cells, with annotation from integration with in vivo data^61^ (finer annotation in Extended Data Fig. 4a). Ex. Endo: extra-embryonic endoderm. **d**, Endogenous expression (normalized UMI counts) for *Sox2* displayed on UMAP projection, highlighting pleiotropic expression in pluripotent (caret) and ectodermal lineages. **e**, scATAC pseudobulk pileup for *Sox2* locus. The caret points to the *Sox2* control region^56,57^. The inset zooms in the core. Regions profiled and differentially accessible in the pluripotent population are shaded in gray. The red carets mark the two cell-type-specific CREs. **f**, Single-cell maps of CRE activity for four CREs (separate panels). Each point represents a single cell. Gray indicates cells with no reporter detected (ND: no detection) for the specified CRE. The color marks reporter expression (average normalized mBC UMI per cell) from none (black) to high (red) for cells with detected reporters (oBC UMI >10). The color axis is truncated to 4 UMIs. Elements chr3_2007 and chr3_2009 have significant expression specific to pluripotent cells (carets) (Fig. 4a, marginal activity from chr3_2005 significant in only one of three biological replicates), mirroring *Sox2* expression in that cell type (c.f. **d**). The number of cells with detected reporter integrations is indicated on each panel. Source data

**Fig. 4:**
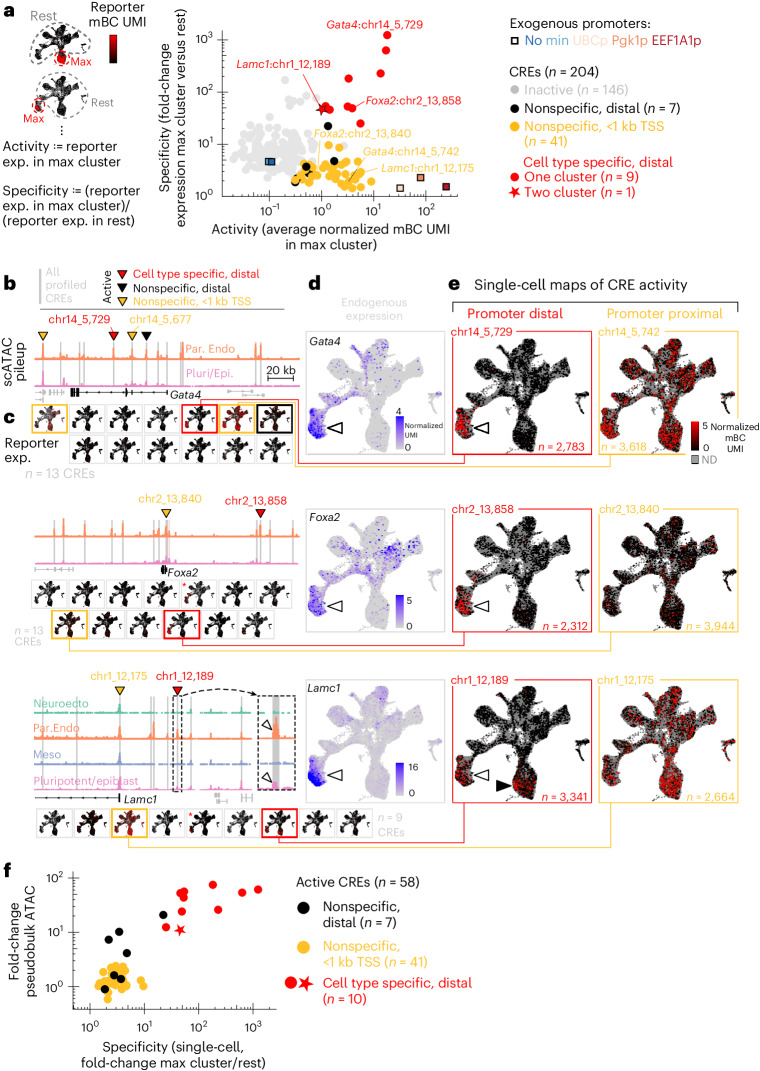
Multiplexed identification of constitutive and autonomous lineage-specific CREs. **a**, Quantification of CRE function (median from three biological replicates). Activity: reporter expression (exp. = average normalized mBC UMI count) in the maximum-expression cell type (defined from fine clusters of Extended Data Fig. 4a). Specificity: maximum-expression cell-type reporter level over expression in all other cells. Active elements (black: nonspecific, distal; orange: nonspecific, <1 kb TSS; red: cell type specific) show excess expression (bootstrap resampling) in all replicates compared to basal controls (no and minimal promoter). Cell-type-specific elements (specificity >5 and significantly higher than cell-type permuted sets) are highlighted (red). CRE *Lamc1*:chr1_1218, active in two cell types, is marked with a star. Exogenous promoters (same as Fig. 2a) are shown as colored squares. **b**–**e** are reproduced for the different loci (top to bottom: *Gata4*, *Foxa2* and *Lamc1*). **b**, Pseudobulk pileup of scATAC (pluripotent and parietal endoderm [Par. Endo]: *Gata4* and *Foxa2*, and also neuroectoderm and mesoderm for *Lamc1*) for 200 kb region centered on gene TSS. The gray shading of peaks indicate regions profiled (shaded red peak near *Foxa2* TSS: peak not in the library due to inability to identify specific cloning primers). The carets point to elements identified as active with scQers. The inset for *Lamc1* locus highlights differential accessibility in both pluripotent/epiblast and parietal endoderm cells. **c**, Single-cell CRE activity maps for all tested elements in the locus. The outline indicates activity of element in assay (coloring as in **a**). The red asterisks mark elements with activity but in <3/3 replicates. **d**, The endogenous expression (scRNA-seq, normalized UMI counts projected on UMAP) for genes corresponding to loci shown. The caret points to the parietal endoderm cells. **e**, Single-cell reporter expression (normalized mBC UMI, projected on UMAP, colormap truncated at 5 mBC UMIs per cell for contrast) for putative promoter (orange) and distal CRE (red) associated with the gene in the locus. The number of cells with detected reporters per element is indicated. The white carets point to parietal endoderm. The black caret (*Lamc1*:chr1_12189) marks reporter expression in pluripotent cells. **f**, The fold change in ATAC (cognate cluster versus rest of cells) versus single-cell reporter expression specificity (definition and color scheme as in **a**) for all active elements identified. Source data

**Extended Data Fig. 4:**
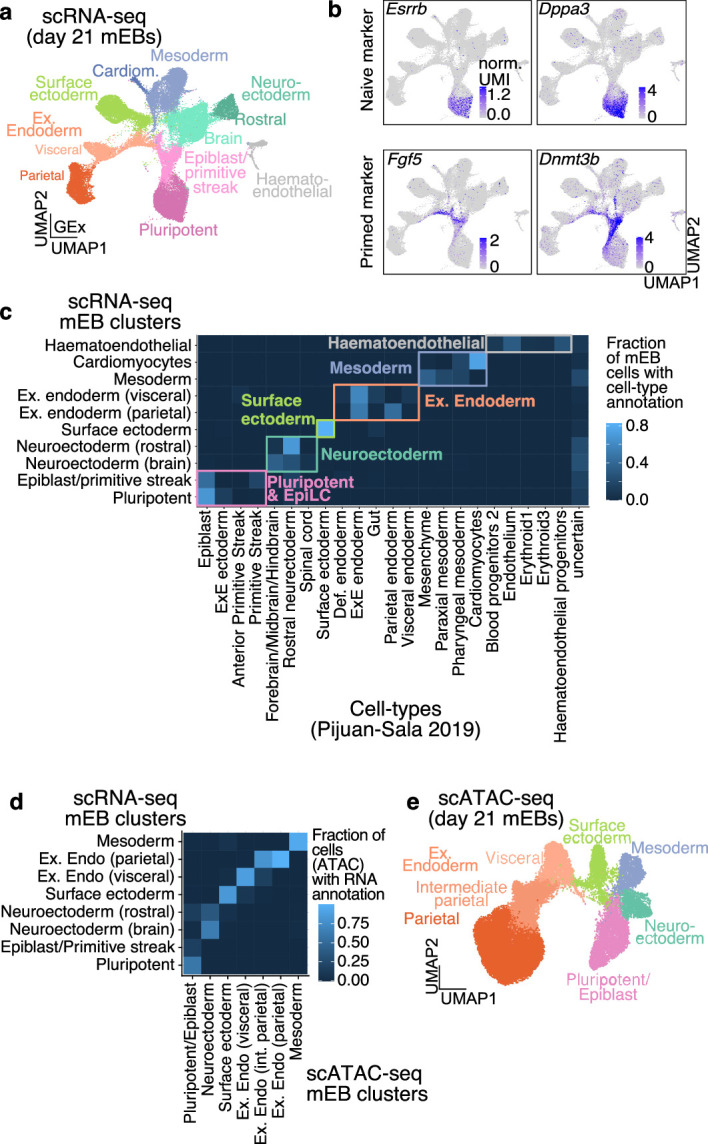
Molecular profiling and integration of single-cell data from 21-day mouse embryoid bodies. **a** UMAP of scRNA-seq data from quality-filtered cells from scQer-integrated, day 21 mEBs (same as Fig. 3c) annotated with fine-resolution cell types derived from label transfer of *in vivo* dataset^61^, as shown in panel **c**. These cluster definitions are used to quantify CRE activity over cell types (for example, Fig. 4a, Extended Data Fig. 5b). **b** Example of naive and primed pluripotent stem cell marker gene expression (normalised UMI counts) displayed on UMAP, used to annotate the respective cells as pluripotent and epiblast/primitive streak. **c** Heatmap displaying fraction of mEB-derived cells (from each cluster in panel **a**) with label transferred to cell-types from *in vivo* data from Pijuan-Sala et al.^61^. Cell types with no associated cells in mEBs (with maximum fraction < 5%) are not included for brevity. Clusters coarse-grained for representation (Fig. 3c) are boxed. Uncertain column corresponds to cells that had ambiguous label transfer. The mEB cluster marked as pluripotent was manually annotated from specific expression of canonical marker genes^63^ in those cells (panel **b**) as a result of a lack of naive mESCs in the integration dataset. **d** Integration of scATAC-seq and scRNA-seq for cluster annotation. Heatmap showing fraction of nuclei from scATAC-seq-derived clusters predicted to be from cell-type identified in scRNA-seq data, displaying unambiguous matches. Certain minor cell types (cardiomyocytes, haematoendothelial) were not found at high proportion in the scATAC-seq data. **e** UMAP of scATAC-seq data from quality filtered cells (n=46408, two biological replicates) from day 21 mEBs. Clusters are labelled based on integration with scRNA-seq data (panel **a**, panel **e**). Source data

**Extended Data Fig. 5:**
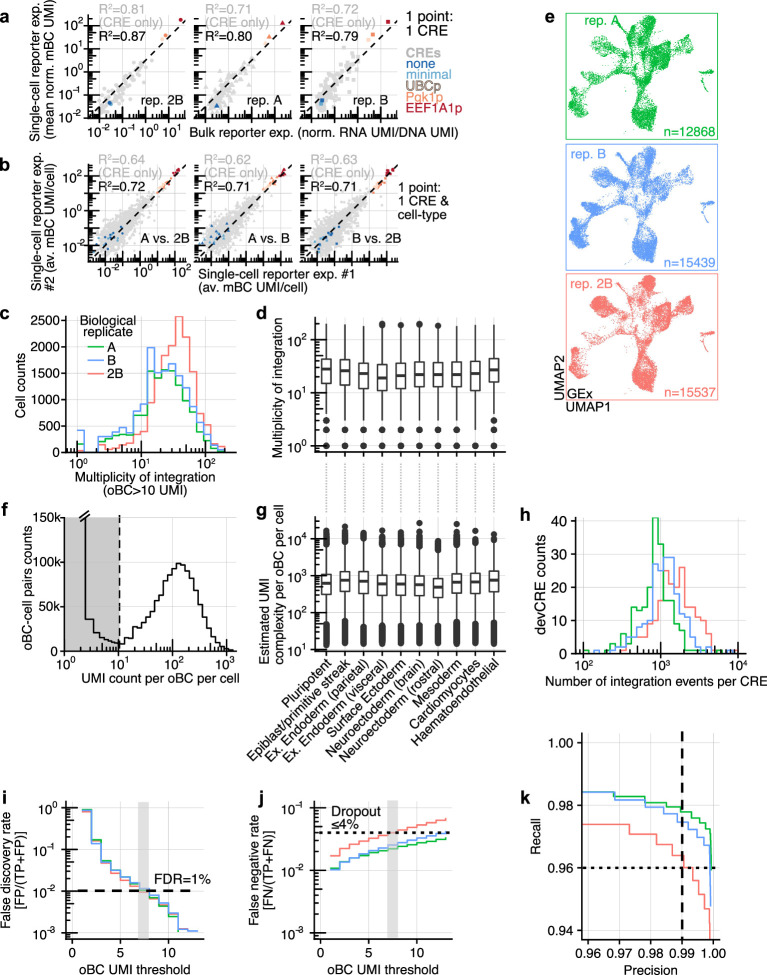
Quality metrics of single-cell reporter assay in mEBs. **a** Comparison between single-cell (average normalised mBC UMI count across all cells with detected reporter) and bulk quantification (day 21 samples, RNA/DNA ratio of summed 1% winsorised UMI counts across all barcodes) for well-represented CREs (>100 integrations, >30 total mBC UMI in single-cell assay, and >35 mBC with at least 20 DNA UMI in bulk assay) stratified by biological replicate. CREs (grey) and promoters coloured according to Fig. 2a, dashed marks a 1:1 slope. R^2^ on log-transformed values, including exogenous promoters (black) or not (grey). **b** Comparison of per-cell type reporter quantification (average normalised mBC UMI over cells in clusters of Extended Data Fig. 4a) for CREs with >0 activity stratified by biological replicates. Each point corresponds to a CRE in a cell-type (10 points per CRE). R^2^ on log-transformed values, including exogenous promoters (black) or not (grey). **c** Distribution of multiplicity of integrations (number of oBC with >10 UMI per cell) across individual cells and stratified by replicate (median: repA=20, repB=19, rep2B=31). High MOI in rep2B likely results from further selecting mCherry+ cells (1% co-transfection), not performed for replicates A and B. **d** Distribution (box plot, centre marks the median, edges of boxes define the 25th and 75th percentiles, whiskers extend to 1.5 times the interquartile range) of multiplicity of integration stratified by cell types (see Extended Data Fig. 5a). Cell type annotations same as in panel **g**. Each box plot is constructed from all cells assigned to a cell type (n=43844 total number of cells over all cell types from three independent experiments). **e** scRNA-seq gene expression UMAP (same as Fig. 3c) stratified by biological replicate (no batch correction) showing reproducibility of cell-types obtained in embryoid bodies derived from reporter-containing mESC. Number of cells for each replicate indicated in each panel. **f** Distribution of oBC UMI counts per cell (similar to Fig. 2c) highlighting robust circular barcode RNA capture in differentiated cells. Sharp bimodality and high signal-to-noise enables high-recovery reporter integration detection. **g** Box plot (centre marks the median, edges of boxes define the 25th and 75th percentiles, whiskers extend to 1.5 times the interquartile range) of estimated total UMI complexity (zero-truncated Poisson) for each captured oBC (>10 UMI) in all cells stratified by cell type, displaying similar levels irrespective of cell type. Each box plot is constructed from all cells assigned to a cell type (n=43844 total number of cells from three independent experiments). **h** Distribution of number of captured integration events per CRE (not including exogenous promoter series, determined from oBC UMI >10 from oBC-associated CRE) stratified by replicates, showing reasonably uniform coverage across profiled elements. **i-k** Precision-recall analysis of oBC detection (similar to Fig. 2h, Extended Data Fig. 3e,f) for mEB-derived cells. Despite only replicate 2B being directly bottlenecked, replicates A and B also displayed (modest) clonal expansion, which enabled analysis of oBC dropout in these samples as well. High-confidence clones with at least two assigned cells are included (repA: 600 clones, 3977 cells; repB: 635 clones, 6465 cells; rep2B: 325 clones, 8518 cells), with results unchanged if restricting to more highly represented clones. Consensus clonotypes served as ground truth for analysis. Panels **h** and **i** respectively show the false discovery rate (FP/[FP+TP]) and false negative rate (FN/[FN+TP]) as a function of the UMI threshold used to assign barcodes to cells. At 1% FDR, false negative (dropout) is less than 4%. oBC libraries from replicate 2B were not sequenced as deeply (average saturation 6.0% vs. 18.7%), suggesting that part of the dropout is due to incomplete sequencing coverage. Source data

Following optimization in cell lines, we sought to apply scQers to discover cell-type-specific CREs in an in vitro model of early mammalian development, mouse embryoid bodies^49,50^ (mEBs). We drew putative CREs for testing from the neighborhood of prioritized developmental loci (Fig. 3a,b). First, by profiling 21-day differentiated mEBs with scRNA-seq and single-cell assay for transposase-accessible chromatin with sequencing^51,52^ (scATAC-seq), we established the transcriptional and chromatin accessibility states of various cell types (Extended Data Fig. 4). scATAC-seq data from mEBs was highly correlated to in vivo data from matched cell types in E7.5–E8.5 embryos^53^ (*R*^2^ log-transformed accessibility across top 65,000 mEB peaks: for example, parietal endoderm 0.77, neuroectoderm 0.78, mesoderm 0.76), supporting mEBs as a model of gene regulation in early development. Leveraging these data, we nominated 22 developmental genes with germ-layer-specific expression and cell-type-specific chromatin accessibility landscapes (Supplementary Data 1) such as endoderm regulator *Gata4* (ref. ^54^), other lineage-defining transcription factors (*Klf4*, *Foxa2* and *Sox17*) and structural genes (laminins, collagens and tubulin). As a comprehensive set^55^ of CREs to profile from these genes, we selected all regions within ±100 kb of their transcription start site (TSS) that were reproducibly highly accessible in the expression-cognate cell type (for example, 13 putative CREs near *Gata4* in Fig. 3a; for other examples, see Fig. 4a). As positive controls, we additionally included the four constituents of the core *Sox2* control region^56,57^ (Supplementary Data 4), accessible exclusively in pluripotent cells (Fig. 3e). In total, 209 elements were included for profiling (145/209 promoter-distal >1 kb from promoters^58^, median element size 937 bp, 893/956 bp 25th/75th percentiles; Supplementary Data 1). The five exogenous promoters (same as Fig. 2a) were also spiked-in as standards. Following library construction and sequential subassemblies (Supplementary Fig. 2, 204/209 CREs represented with >20 oBC–mBC pairs, 88/145/242 10th/50th/90th percentile number of valid oBC–mBC pairs per CRE), scQers were integrated in mouse embryonic stem (mES) cells at high MOI using piggyBac^59,60^ (Extended Data Fig. 5c,d; median MOI, 23; per-cell probability of oBC–CRE–mBC triplet being integrated more than once, 1%). Reporter-integrated cells were induced to form mEBs, sampled every 2 days for bulk MPRA quantification across differentiation and scQered at the 3 weeks end-point (Fig. 3b).

### High performance in a stem-cell derived developmental system

mEBs reproducibly comprised diverse cell types unambiguously mappable to in vivo germ layers^61^ (Fig. 3c, *n* = 43,799 pass-filter cells across three biological replicates (replicates 1 and 2: separate transfections; replicate 2B: ~500-clone bottleneck of replicate 2 with 12% identified clonotypes overlap to replicate 2 and, thus, largely orthogonal; all replicates separate mEB inductions) Extended Data Fig. 5e), confirming successful differentiation despite the presence of reporters at high MOI.

scQers displayed high performance in mEBs. First, oBCs were robustly captured (median library complexity, 836 UMI/oBC/cell), displaying a bimodal distribution of oBC UMI/cell (Extended Data Fig. 5f). oBC expression was cell type independent (Extended Data Fig. 5g), enabling uniformly high recovery (<4% oBC dropout at FDR of 1% from precision–recall analysis of clonal cells; Extended Data Fig. 5i–k). Second, comparison of end-point bulk and single-cell quantification across profiled CREs confirmed accuracy of reporter expression measurement over the full dynamic range (*R*^2^ log-transformed activity 0.81; Extended Data Fig. 5a), and per-cell-type quantification was reproducible (*R*^2^ log-transformed across replicates 0.72; Extended Data Fig. 5b). Representation was reasonably uniform across tested CREs (Extended Data Fig. 5h; captured integration events per element 1,597/3,153/6,197 10th/50th/90th percentiles, and *n* = 17,971–34,745 for exogenous promoters).

### Single-cell expression maps from *Sox2* control regions

**Extended Data Fig. 6:**
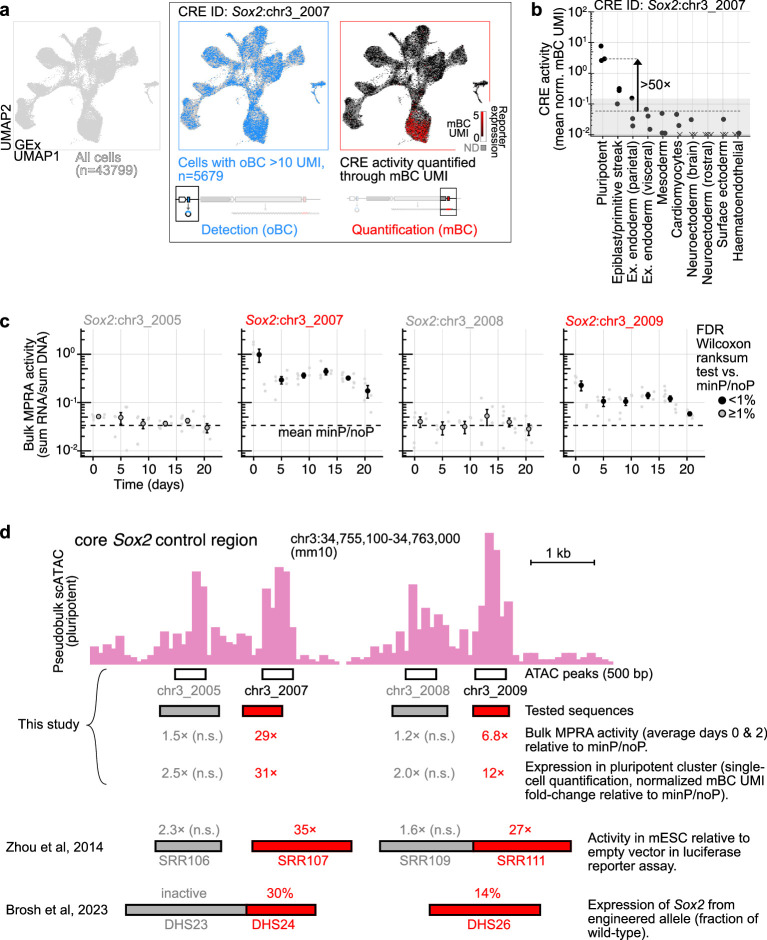
Details on activity of constituent elements of the Sox2 control region. **a** Illustration of the steps to construct a single-cell map of CRE activity for a given regulatory element. Left: All cells passing quality filters are initially considered. Middle: Reporter detection. The list of oBCs associated with the CRE of interest (here *Sox2*:chr3_2007, see Fig. 3f) from the predetermined oBC-CRE-mBC dictionary are identified. Cell barcodes with one (or more) CRE-associated oBC with >10 UMI are retained (n = 5679), shown in blue on the UMAP (grey corresponding to cells with no detected reporters to the CRE of interest). Right: Expression quantification. From the oBC-CRE-mBC dictionary, the UMI counts to CRE-associated mBC are collected. In cases where multiple reporters to the same CRE (but different oBC-mBC pairs) are detected in the same cell, the average mBC UMI is taken. To correct for the fact that some cell types have more RNA (or other technical factors), we normalise the mBC expression by the total UMI to the transcriptome for each considered cell. The resulting single-cell reporter expression can then be layered on the low dimensional projection (black low to high red), enabling visualisation of CRE activity across the manifold of cell states in the system. **b** Quantification of the average reporter expression (average normalised mBC UMI, see panel a) across cells from different cell types (defined as clusters in Extended Data Fig. 4a). Each dot corresponds to a biological replicate. Crosses correspond to cell types/replicates with average expression below 0.01 mBC UMI/cell. Arrow marks the fold change in expression between the maximum cluster (pluripotent) and the rest of cells (defined as specificity in Fig. 4a). Grey shading marks the noise floor determined from variability from the basal expression controls (minimal and no promoter). **c** Bulk MPRA quantification of the four constituents of the core *Sox2* control region (see Extended Data Fig. 7 for all CREs), showing consistent results with single-cell quantification (inactive: *Sox2*:chr3_2005, *Sox2*:chr3_2008; active: *Sox2*:chr3_2007, *Sox2*:chr3_2009). Small grey points mark individual replicates and time points. Large points are the average over n = 3 biological replicates from consecutive time points, and are filled if significantly above the basal expression controls (one-sided ranksum test, B-H corrected, <1% FDR). Error bars show the standard error of the mean. Dashed line indicates the mean of basal expression control (minimal and no promoters). The observed decrease in activity over time for *Sox2*:chr3_2007 and *Sox2*:chr3_2009 is consistent with pluripotent cells being progressively depleted from the population, thereby leading to decreased activity when averaged over all cells in bulk. **d** Sox2 control region scATAC pseudobulk pileup in pluripotent/epiblast cluster (zoom in of Fig. 3e). Under pileup, elements tested (in the same genomic position reference frame as the pileup, Supplementary Data 4 for positions) are indicated both from this study (top: 500 bp regions peak from ArchR pipeline; bottom: PCR-amplified tested sequences), and two previous studies quantifying reporter activity, Zhou et al.^57^, and Brosh et al.^7^. Grey regions were not found to be significantly active. Red regions were found to have activity in pluripotent cells (measured activity is indicated). *Sox2*:chr3_2007 from this study was not entirely nested in previously tested elements (SRR107 and DHS24), suggesting that even higher activity than measured might be achievable with a more inclusive element. The slight misalignment from the ATAC peak for *Sox2*:chr3_2007 resulted from lack of identifiable specific PCR cloning primers in the immediate 3′ region. Source data

scQers generated high-contrast single-cell maps of CRE activity (Extended Data Fig. 6a,b). As a case study, we considered gene expression control of the pleiotropic regulator *Sox2* (Fig. 3d). *Sox2* is a key factor in pluripotency maintenance^57^. Central to *Sox2* control is a distal (~135 kb from TSS) cluster of CREs necessary for driving high expression in pluripotent cells^56,57^, previously shown to function autonomously^57,62^. Of four differentially accessible elements in pluripotent cells from this control region (Fig. 3e, inset), two displayed robust activity (Fig. 3f, red; 10–30-fold higher expression versus basal controls), in agreement with previous characterization^7,57^ (Extended Data Fig. 6d and Supplementary Data 4) circumscribed to the pluripotent population (for example, >50-fold higher expression versus other cell types for *Sox2*:chr3_2007). While *Sox2* was expressed in the pluripotent and ectoderm lineages in mEBs (Fig. 3d), CREs from *Sox2* control regions were exclusively active in pluripotent cells (*Essrb*/*Dppa3* positive^63^; Extended Data Fig. 4b). Our results on this previously characterized cluster of regulatory elements confirm that scQers can report cell-type-specific expression in a multicellular system with high sensitivity and contrast. scQer experiments on six additional literature-selected cell-type-specific CREs^64–67^ further confirmed the robustness of our approach (3/6 with expected activity profiles, 3/6 inactive in mEBs; Supplementary Fig. 3h,i and Supplementary Data 4).

### Systematic identification of active CREs

**Extended Data Fig. 7:**
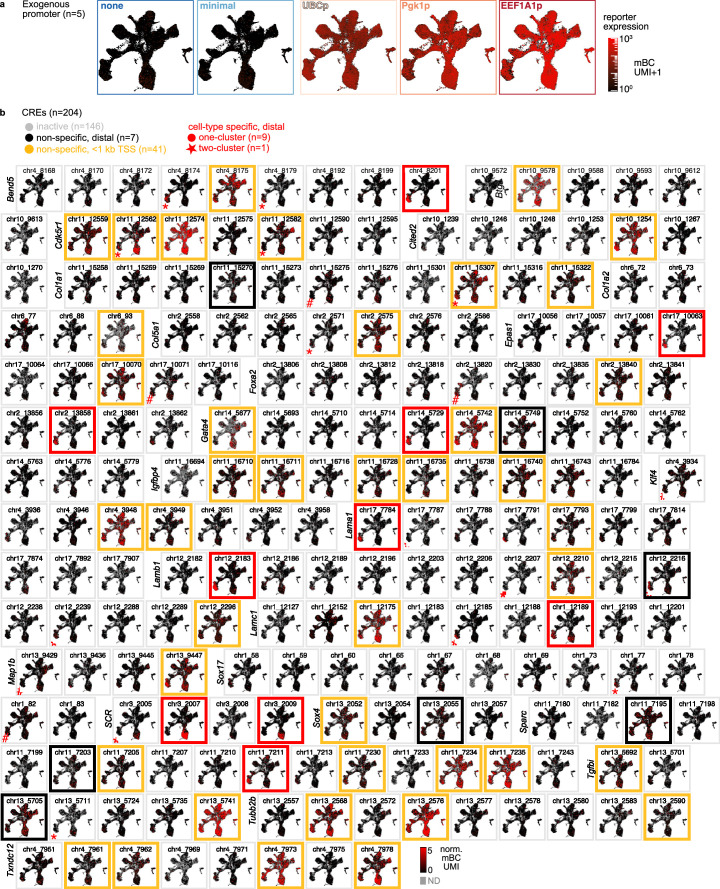
Systematic characterization of 204 putative CREs in mouse embryoid bodies. **a** Single-cell reporter expression (average normalised mBC UMI per cell) for the five exogenous promoters used as internal controls. Colour scale is logarithmic (with a pseudocount of 1). **b** Single-cell reporter expression maps for the 204 profiled CREs. Elements are organised by locus (horizontally). Map outlines indicate the element class as classified in the two-dimensional phenotypic space from Fig. 4a. Elements marked with # are found to be active (non-specific) in 2/3 replicates. Elements marked with * are found to be active and specific in at least one replicate with our thresholds. Each map is shown to the same colour scale (normalised mBC UMI from 0 and truncated to 5). Source data

We also quantified both activity and cell type specificity of other tested candidate CREs (*n* = 200), identifying multiple active elements (Fig. 4a and Extended Data Fig. 7). For each CRE, average reporter expression was determined across cells with detections, stratified by cell type. Activity was defined as the maximum per-cell-type reporter expression, while specificity was taken as the maximum per-cell-type mBC expression divided by the mean expression in all other cells (Fig. 4a). We identified 58/204 endogenous CREs with activity in significant excess of the basal controls in all three replicates (bootstrap *P* < 0.05, Methods, Supplementary Data 5). The elements with the highest expression were the active exogenous promoters (UBCp, Pgk1p and EEF1A1p) at ~30–250 mBC UMIs per cell (levels ~300× to ~2,500× above basal controls; Fig. 4a). Active endogenous CREs spanned a wide range at lower expressions (maximum per-cell-type expression ~0.3–20 mBC UMIs per cell; Fig. 4a). Notably, a sizable fraction (19/58) of the active CREs had expression under 1 mBC UMI per cell, and most were below the chimeric read threshold of 10 UMIs per mBC per cell, underscoring the usefulness of a high-sensitivity method.

**Extended Data Fig. 8:**
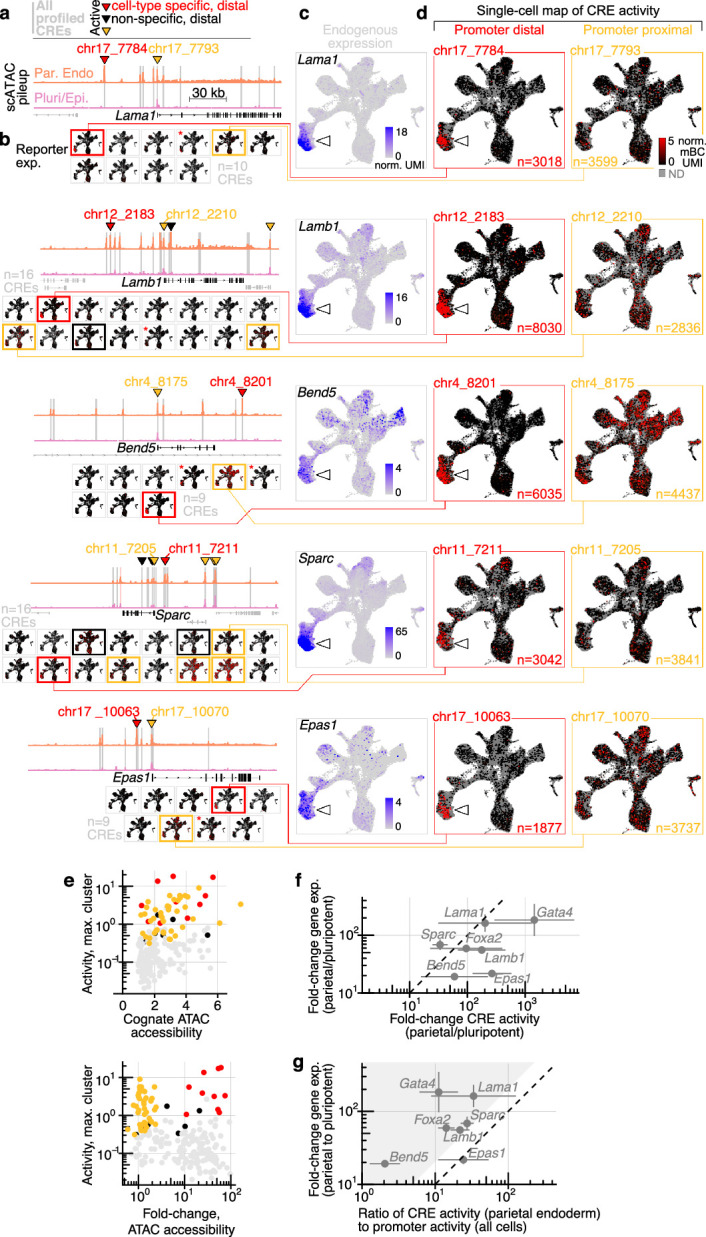
Additional loci with lineage specific distal CREs. **a-d** Same as Fig. 4b–e, but for the additional five loci for which cell-type-specific CREs were identified. Each panel a-d is reproduced across rows for the different loci (top to bottom: *Lama1*, *Lamb1, Bend5, Sparc, Epas1*). The pink shaded element at the *Sparc* locus (chr11_7186) could not be cloned by PCR due to inability to identify specific primers in the vicinity. **e** Measured maximum (across cell-types) activity (y=mean norm. mBC UMI/cell) vs. chromatin accessibility (top, x=number of ATAC reads in peak normalised to in TSS reads×10^−4^ in the cognate cell-type) and fold-change in chromatin accessibility (bottom: x=fold-change accessibility in cognate cell type over other cell types). Points are coloured based on their functional categorization (same colours as Fig. 4a, grey: inactive, black: non-specific, distal; orange: non-specific, <1 kb TSS; red: cell-type specific). **f** Fold-change in gene expression (y-axis, ratio normalised UMI in parietal endoderm to pluripotent) vs. CRE induction (x-axis, fold-change reporter levels, average normalised mBC UMI in parietal endoderm over pluripotent) for parietal-endoderm-specific distal CREs. Dashed line is 1:1. Geometric mean over three biological replicates is shown (errorbar: standard deviation of geometric mean). **g** Assessing recapitulation of endogenous expression from identified autonomous CREs. Each point corresponds to one of 7 parietal endoderm genes with putatively associated identified active CREs and promoters shown in Fig. 4 and panels a-d above (for example, *Lamb1*: CRE chr12_2183, promoter chr12_2210; CRE associations to genes are putative). Endogenous gene induction (y-axis): fold-change in endogenous gene expression (average in normalised UMI counts) from pluripotent to parietal endoderm. CRE induction over promoter baseline (x-axis): CRE activity in parietal endoderm (reporter level, average normalised mBC UMI parietal endoderm) over mean activity of associated promoter in all cells (reporter level, average normalised mBC UMI). Dashed line is 1:1. Shaded area corresponds to (CRE induction)<0.5×(gene expression induction). Geometric mean over three biological replicates is shown (errorbar: standard deviation of geometric mean). Source data

Active CREs displayed distinct expression patterns across mEB cell types. Categorizing active CREs as cell type specific versus nonspecific (permutation test), we found 10/58 developmental CREs with reproducible cell-type-specific activity (red in Fig. 4a–c and Extended Data Fig. 8a–d). Singleton validation experiments on the eight most specific CREs confirmed that the elements drove cell-type-specific expression (Supplementary Figs. 4 and 5). Of the remaining 48 nonspecific active elements, 41 (85%) were promoter proximal (for example, orange in Fig. 4e and Extended Data Fig. 8d) compared to 0/10 of cell-type-specific CREs. Conversely, 41/62 tested promoter-proximal elements were found to be active and nonspecific (while 0/62 were cell type specific). Consistent with their function and distance from TSS, all cell-type-specific CREs showed >10-fold change in chromatin accessibility in their cognate cell types; in contrast, promoters were constitutively open (<3-fold change; Fig. 4f). Notably, accessibility (rather than change in accessibility) was a poor predictor of activity or specificity (Extended Data Fig. 8e), in line with evidence of the imperfect correspondence between accessibility and function for regulatory elements^55,68^. Single-cell activity maps thus delineated two broad patterns of autonomous function: constitutively active elements (overwhelmingly TSS proximal, broadly accessible) and cell-type-specific elements (overwhelmingly TSS distal, differentially accessible).

Our assay relies on high MOI random integration of reporters for scalable multiplexing, raising concerns that genomic positional effects might dominate the signal^45,46^. To assess positional effects, we bottlenecked reporter-integrated mES cells to a few hundred clones in one of the replicates (replicate 2B) before mEB induction. Quantifying activity of the 10 cell-type-specific CREs across well-represented clones, we found that most CREs (9/10) retained specificity (>5-fold) across the super-majority (>2/3) (Supplementary Fig. 6 and Supplementary Data 6), suggesting that positional effects can be averaged over.

### Characterization of lineage-specific, autonomous CREs

Of the ten autonomous cell-type-specific CREs identified, two belonged to the core *Sox2* control region (Fig. 3f), while the remaining eight, all from distinct parietal endoderm-expressed loci (red Fig. 4e, Extended Data Fig. 8d), included a *Gata4* intronic CRE 10 kb downstream of the first exon (chr14_5729; Fig. 4e, second row) and an CRE 70 kb upstream of *Foxa2* (chr2_13858; Fig. 4e, third row). One active element at the *Lamc1* locus (chr1_12189; Fig. 4e, fourth row) was found to be active in two cell types, with concordant chromatin bi-accessibility (Fig. 4b, inset, fourth row). Identifying mostly endoderm-specific CREs was not unexpected given the uneven sampling of tested elements due to the high proportion of endoderm cells in the scATAC data.

Reporter expression driven by developmental CREs mirrored the predominant pattern of expression of their nearby putatively associated gene (Fig. 3d versus Fig. 3f, Fig. 4d versus Fig. 4e, and Extended Data Fig. 8c versus Extended Data Fig. 8d), except for the bifunctional putative *Lamc1* CRE (Fig. 4d, fourth row, black caret), which drove expression in both parietal endoderm and pluripotent cells, in contrast with endogenous *Lamc1* whose expression was restricted to parietal endoderm. For endoderm-specific CREs, the magnitude of activity induction was on par with endogenous gene induction (Extended Data Fig. 8f,g and Supplementary Note 2).

**Extended Data Fig. 9:**
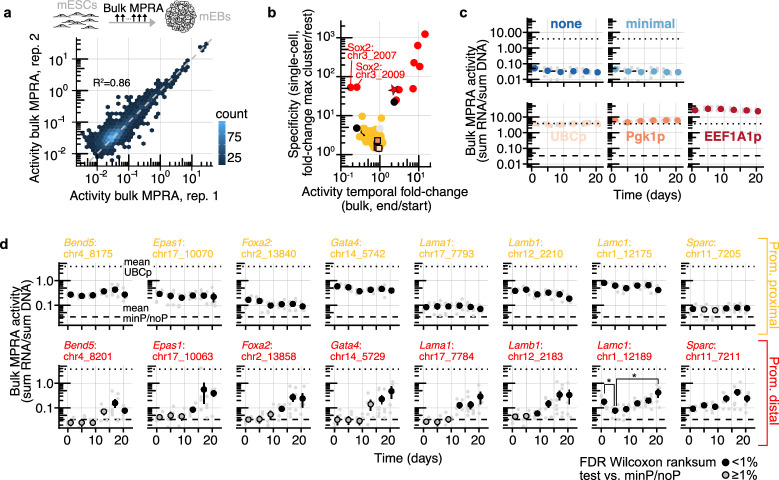
Cell-type-specific CREs are temporally dynamic along mEB differentiation. **a** Reproducibility of bulk MPRA measurement. Comparison of bulk MPRA activity (RNA/DNA ratio of summed 1% winsorised normalised UMI counts) for all CREs in two biological replicates (>10 measured barcodes in both replicates, including exogenous promoters) at all time points (n = 2508 comparisons, R^2^ from log-transformed activity). **b** Differentiating EBs were sampled every two days at passage from all replicates, and bulk RNA/DNA MPRA libraries were generated. Fold-change in bulk MPRA activity across time course (mean activity day 20.5 over mean day 1) was compared to the observed specificity of elements as quantified from the scQer end-point quantification (Fig. 4a). Elements shown found to be active in either bulk or single-cell assays are shown and coloured according to class (red: cell-type specific, orange: non-specific, <1 kb from TSS, black: non-specific, distal ≥1 kb TSS). The one grey point corresponds to the single element found to be active in bulk but not single-cell assay. Active exogenous promoters (UBCp, Pgk1p, EEF1A1p, panel b) are shown as squares. There is a correspondence between cell-type specificity and temporal change from the bulk assay. Bulk temporal fold-change is 5-10x smaller compared to single cell quantification likely due to bulk assay averaging activity from all cell-types. **c** Activity traces of bulk MPRA time quantification for the exogenous promoters included as internal controls. Small grey points correspond to activity (RNA/DNA ratio of summed 1% winsorised normalised UMI counts) from different replicates/time points. Large points are the average of three biological replicates from two adjacent time points, with error bars corresponding to standard deviation of the mean (smaller than symbol size). Average of basal expression controls (no and minimal promoters) is shown as the dashed line, and the dotted line corresponds to the mean UBC promoter activity (reproduced in panel d for scale). **d** Same as panel c, but for active cell-type-specific CREs (red) and promoters (orange) from the loci shown in Fig. 4 and Extended Data Fig. 8. Points are filled when significantly above basal expression controls (one-sided ranksum test, B-H corrected, FDR < 1%). Promoters (orange) show largely constant expression over time. CREs (red) show substantial induction over the time course. Bifunctional CRE *Lamc1*:chr1_12189 displays initial decrease followed by and increase consistent with its activity in both undifferentiated and differentiated cells (one-sided Bonferoni corrected ranksum test between day 1 and day 5, p = 0.026; and between day 5 and day 20.5, p = 0.017). Source data

Leveraging our time-resolved bulk MPRA (Extended Data Fig. 9 and Supplementary Data 7) on the same samples, we found a consistent set of active CREs (53/54 bulk active elements identified as active from scQers, 53/58 scQers identified elements found as bulk active). Importantly, elements found to be cell type specific with scQers displayed either temporal increase (Extended Data Fig. 9d, red), decrease (core *Sox2* control region; Extended Data Fig. 6c) or nonmonotonic behavior (bifunctional CRE, *Lamc1*:chr1_12189; Extended Data Fig. 9d), supporting their classification as developmental regulatory elements. In contrast, active but nonspecific elements displayed little temporal variation across differentiation (for example, exogenous promoters and endogenous elements; Extended Data Fig. 9c,d, orange), as expected for constitutive, promoter-like, CREs. A number of CRE features (for example, accessibility and number of transcription factor binding sites; Supplementary Note 3 and Supplementary Fig. 7) correlated with measured activity.

**Extended Data Fig. 10:**
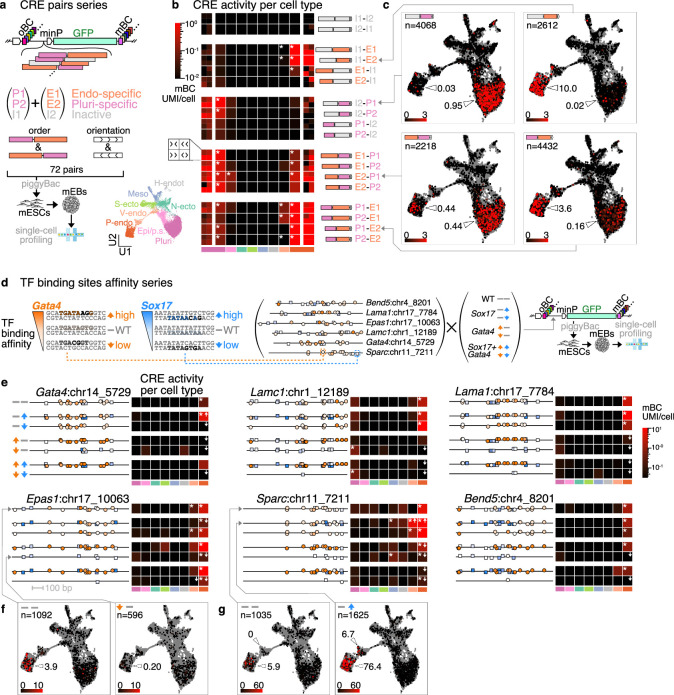
Additional applications of scQers: pleiotropic activity of synthetic CRE pairs & profiling CREs with disrupted/optimised putative transcription factor binding sites. **a** Library of pairs of CREs were constructed by joining two pluripotent (P1: *Sox2*:chr3_2007, P2: *Sox2*:chr3_2009) and one inactive sequence (I1: *Cdk5r1*:chr11_12590) with two parietal endoderm (E1: *Epas1*:chr17_10063, E2: *Gata4*:chr14_5729) and another inactive sequence (I2: *Col5a1*:chr2_2586). Combinatorial libraries with all possible orientations and orders of the 6 components were cloned in scQers, mapped to barcodes with nanopore sequencing, integrated into mESCs and profiled for activity in mEBs. Inset shows UMAP of cells passing QC (n = 20477), coloured by the mapped cell type (Pluri: pluripotent, Epi/p.s.: epiblast/primitive streak, N-ecto: neuroectoderm, H-endot: haemato-endothelial, Meso: mesoderm, S-ecto: surface ectoderm, V-endo: visceral endoderm, P-endo: parietal endoderm). **b** Cell type-specific activity (median norm. mBC UMI per cell over three biological replicates) per cell type per construct. Rows indicate different pairs of CREs (in specified order), and columns correspond to different cell types (based on the colour scheme of the inset in a, indicated at bottom). Two outermost columns of the heatmap stratify each CRE pair by relative orientation of its components for their activity in pluripotent (leftmost column) and parietal endoderm (rightmost column), central columns correspond to median over all four relative orientations for a given ordered pair. Stars (*) mark CRE pairs and cell types with activity significantly above negative controls (minP, noP, I1-I2, I2-I1) (p < 0.01 from one-sided bootstrap resampling of cells with detected constructs with B-H correction). **c** Example single-cell maps of CRE-pair activity. Number of cells with detected CRE-pairs marked, with norm. mBC UMI/cell shown on a black (low) to red (high) colour scale (grey: CRE of interest not detected). Quantified expression in parietal endoderm and pluripotent cells (median over biological replicates of mean norm. mBC UMI/cell) are indicated. **d** CRE variants optimising and disrupting the binding affinity of all putative *Gata4* and *Sox17* transcription factor binding sites in combination (variants: WT, *Sox17*-high, *Sox17*-low, *Gata4*-high, *Gata4*-low, *Gata4*-*Sox17*-high, *Gata4*-*Sox17*-low) identified within 6 parietal endoderm-specific CREs were designed based on UniProbe data^106^ (example of approach illustrated in Supplementary Fig. 7d). Schematics of CREs with mapped TF binding sites are shown (*Gata4*: orange, *Sox17*: blue; hue indicative of binding affinity). *Gata4* and *Sox17* putative binding sites within the *Sparc*:chr11_7211 element and their perturbed instances (affinity optimization or disruption via two mutations per site) are displayed as examples. Variant CREs were cloned as a scQer library, and their activity profiled after integration to mESCs and embryoid body differentiation (same experiment as panels a-c). Insert shows UMAP of cells coloured by cell type assignment. **e** Cell type-specific activity (median norm. mBC UMI/cell over biological replicates) per cell type per CRE. Panels show heatmaps of activity of different CRE series, with CRE TF binding site maps shown (left, rows), and columns correspond to different cell types (based on colour scheme of the inset in a, indicated at bottom). Stars (*) indicate significantly higher expression than negative controls (p < 0.01 from one-sided bootstrap resampling of cells with detected constructs with B-H correction). White arrows mark regions with significantly different expressions than the respective WT CRE variant (one-sided bootstrap resampling of cells with detected constructs with B-H correction; up arrow: increased expression p < 0.01; down arrow: decreased expression p < 0.01 if also WT expression > 0.1 mBC UMI/cell). **f-g** Example single-cell maps of CRE activity (for each respective panel, left: unperturbed CRE, right: perturbed CRE). Number of cells with detected CRE reporters indicated, with norm. mBC UMI/cell shown on a black (low) to red (high) colour scale (grey: CRE of interest not detected). f Example of loss-of-activity from disruption of putative *Gata4* sites within CRE *Epas1*:chr17_10063, with mean activity in parietal endoderm indicated. g Dramatic instance of gain-of-function, with >10-fold greater expression in parietal endoderm from putative *Sox17* TF binding site optimization in CRE *Sparc*:chr11_7211, also associated with ectopic expression in the related visceral endoderm. See also Supplementary Figs. 3 and 7. Source data

Overall, scQers enabled the scaled high-sensitivity characterization of both constitutive promoter-like and lineage-specific autonomously active regulatory elements across diverse cell types of 21-day mouse EBs, with CRE activity profiles matching expression of their putatively associated genes. Additional experiments with synthetic pairs of CREs and elements with optimized/disrupted transcription factor binding sites (Supplementary Note 4, Extended Data Fig. 10, Supplementary Fig. 3 and Supplementary Data 8 and 9) confirmed the usefulness of scQers to study regulatory elements.

### Influence of reporter architecture on expression output

scQers rely on a Pol III cassette in proximity to the Pol II promoter driving reporter mRNAs, raising concerns of interference between the two. To assess possible interaction, we constructed libraries with and without the U6/oBC cassette harboring the same putative CREs and promoters (Supplementary Fig. 8a and Supplementary Data 10), integrated the reporters in mES cells, differentiated the cells to embryoid bodies and performed bulk MPRA at various time points. The measured expression driven by the CREs was highly concordant with versus without the Pol III cassette both for promoters and CREs (Supplementary Fig. 8b,c; *R*^2^ of log-transformed activities >0.84). Importantly, temporal induction of the cell-type-specific CRE did not depend on the presence of the U6-driven RNA (Supplementary Fig. 8d,e). While these data do not exclude possible interference from the Pol III in all contexts, they suggest that such influence is of limited magnitude for scQers.

Given our reporter architecture, with the CRE directly upstream of the minimal promoter, we also sought to assess whether the measured mBC counts derived from eRNAs^69^ or from initiation at the minimal promoter. To do so, we tested expression from reporters with and without the minimal promoter, as well as constructs placing the CREs downstream (Supplementary Fig. 8a). Surprisingly, we found little difference in the measured expression comparing reporters with and without minP (Supplementary Fig. 8f,g), suggesting either cryptic transcription initiation (analogous to transcription initiation within the bacterial origin of replication in the original STARR-seq assay^70^), or initiation within the CREs themselves (that is, eRNAs). In addition, although positioning CREs downstream of the reporter cassette compressed the dynamic range of expression (Supplementary Fig. 8h), in line with previous systematic comparison of different MPRA architectures^13^, induction was detectable in 7/13 expected cases (Bonferroni-corrected Wilcoxon test *P* < 0.05; Supplementary Fig. 8i,j), consistent with some of the identified CREs having enhancer activity. Given the possible distance dependence of functional expression outcome to CRE positioning, more experiments will be needed to fully ascertain the molecular origin of the measured mBCs. Despite the prevalence of the CRE-minP-reporter architecture for MPRA assays^15,71–74^, there exist no 5′ end mapping data to our knowledge in that context. As such, our results draw an important distinction between reporter and enhancer assays. While this does not undermine the unique advantages of scQers to identify elements driving cell-type-specific mRNA production, researchers seeking to unambiguously measure enhancement of transcription initiation at a specified site should insulate the enhancer from the promoter or consider alternative architectures.

## Discussion

CREs orchestrate the precise unfolding of development in metazoans, enabling the emergence of a species’ form and function from a genomic blueprint. However, our ability to study developmental CREs at scale has been constrained, particularly in mammalian systems. We and others^75–77^ have recognized that a simple path forward is to intersect MPRAs with single-cell resolution technologies. Here, we overcome key technical challenges of combining these two modalities, resulting in scQers, an MPRA that decouples the detection and quantification of reporters via a dual RNA system and circularization-based enhancement of barcode recovery. scQers extend measurements into a regime fundamentally inaccessible with traditional multiplex reporters, yielding an accurate, precise and high-contrast readout of reporter mRNA levels. Beyond reporter assays, the use of oBCs, and Tornado-based stabilization more generally, may be of broad utility for robust capture in single-cell and other genomics applications ranging from CRISPR screens to cell lineage tracing.

The relatively low hit rate of our screen (8/200 cell type specific) suggests that random genome integration followed by differentiation provides a strong filter for elements autonomously competent to reconfigure chromatinized landscapes and drive expression. In addition, lack of activity might be a consequence of our use of a minimal promoter, as opposed to bona fide developmental promoters. Recent systematic studies have found promoter choice to be important in scaling the response of regulatory elements^78–80^. Beyond these technical differences, given the complex multi-CRE landscapes considered here, some tested CREs might contribute to regulation, but only in the presence of (or by directly serving as) cooperating elements, in line with recently described facilitators^8^ or chromatin-dependent enhancers^11^ (for example, tested but inactive *Sox2*:chr3_2005, which overlaps with facilitator DHS23; ref. ^7^). While most elements identified here display expression patterns mirroring that of their putatively associated gene, in-genome perturbations will be necessary to confirm their role, if any, in regulation. As they become broadly available, high-resolution enhancer-to-promoter contact maps^81,82^ could be used to prioritize CREs and further strengthen conclusions drawn from reporter measurements.

How many regulatory elements can be profiled with scQers? Based on current measurements, we estimate that 100 detections per CRE per cell type would robustly detect expressions of 1 UMI per mBC per cell. The number of single cells that need to be profiled per replicate per CRE is thus estimated to be 100 × (number of cell types)/MOI (Supplementary Note 5). The majority of the costs remain on the single-cell assay if using existing commercial droplet-based approaches. With continuous improvement in capture from alternatives, for example, single-cell combinatorial indexing^83^, we anticipate that >10-fold improvement in throughput will soon be achievable.

Several limitations of the current instantiation of scQers are worth noting. First, we currently leverage a high multiplicity of random integrations to boost power. While we show that positional effects can be averaged over to yield robust signal, the different integration positions do contribute to variability in measured expression. Second, not all cell types are amenable to plasmid transfection and transposase integration. Establishing compatibility with lentiviral delivery in particular will require substantial changes in architecture, both to minimize template-switching-mediated scrambling of predetermined oBC–CRE–mBC triplets and to avoid Tornado ribozymes confounding RNA packaging. Finally, as discussed above, alternative reporter configurations will be necessary to unambiguously discriminate between enhancer activity and possible eRNA expression.

As predictive models of regulatory activity improve^11,18,19,84–86^, quantitative experimental approaches are needed to iterate through design–test–learn cycles and validate underlying mechanistic hypotheses. Benchmarks in cell lines, a proof-of-principle screen in a multicellular stem-cell model and experiments on synthetic pairs and mutated CREs establish scQers as a scalable platform for probing gene regulation that should be portable to other developmental systems (for example, zebrafish^87^, *Ciona intestinalis*^27^, the chicken neural crest^88^, synthetic embryoids^89,90^ and in vivo neuronal subtypes with adeno-associated virus derivatives^91^). Although established here with a focus on developmental biology, we envision scQers may also facilitate the identification, optimization and compactification of highly active cell-type-specific CREs for application in gene therapy and other practical uses^92,93^.

## Methods

Primers, oligos and plasmids are listed in Supplementary Data 11. Maps of final amplicons and plasmids are on GitHub^94^. Additional methods details are provided in Supplementary Note 6. Source data are available in Supplementary Data 12–18 and Source Data Extended Data Figs. 1–10.

### scQer single-cell libraries preparation and sequencing

Each 10x lane provides three scRNA-seq libraries (gene expression, mBC and oBC). Library preparation follows the protocol from the manufacturer (steps number listed in this section refer to: v3.1 manual CG000205 Rev D, 10x Genomics, but probably applicable to updated versions with little modification) until step 2.2a (first complementary DNA amplifications). At that step, it is critical to spike-in primers specific to the mBC and oBC reporters (oSR38 and oJBL246, respectively) to a final concentration of 0.5 µM. This will ensure higher capture of the reporter RNAs and will help limit the number of PCR cycles overall. Following cDNA amplification, cleanup proceeds per the protocol (with gene expression and mBC components in the pellet fraction 2.3Ax, and oBC in the supernatant fraction 2.3Bxiv). After step 2.3, gene expression libraries are completed following the manufacturer’s protocol. We note that gene expression, oBC and mBC libraries can all be sequenced on the same Illumina Nextseq run with the design described below.

#### oBC libraries

Final oBC libraries are generated by a semi-nested second PCR using amplified cDNA (55% of fraction 2.3Bxiv as template) in 100 µl using Nextera P5 primers (for example, NextP5_index1) and custom-indexed P7 primers (for example, oJBL425-oJBL427). For example: 50 µl 2× KAPA2G Robust HotStart ReadyMix (Roche), 12.5 µl amplified cDNA from step 2.3Bxiv (supernatant), 5 µl 10 μM NextP5_index1 primer, 5 μl 10 μM oJBL425 primer, 0.5 µl SYBr green 100×, and water to 100 µl; run parameters: 3 min at 95 °C, followed by cycling with 20 s at 95 °C, 20 s at 60 °C and 20 s at 72 °C. To avoid overamplification, the reactions are tracked by qPCR and stopped at or below the inflection point. Given high expression of oBC, five to seven PCR cycles are typically sufficient to get high-concentration libraries. The resulting amplified libraries are purified by 1.5× Ampure XP beads (Beckman Coulter). To avoid loop-the-loop products, the lowest band (207 bp, amplicon: PCR2_oBC_10x_scQer.gbk on GitHub) can be size-selected before sequencing by polyacrylamide gel electrophoresis (PAGE) purification.

Sequencing of the oBC libraries follows the following structure: read1: primer standard Illumina Nextera read1, ≥28 cycles (cell barcode, UMI), index1: custom oJBL432, 6–10 cycles (sample index), read2: custom oJBL433, ≥16 cycles (oBC).

#### mBC libraries

Final mBC libraries (here, mRNA molecules captured from poly-dT reverse transcription primers) are generated with two steps of PCR, first a semi-nested PCR2 followed by an indexing PCR3. PCR2 conditions: 50 µl 2× KAPA2G Robust HotStart ReadyMix, 12.5 µl amplified cDNA from step 2.3Ax (pellet), 5 μl 10 μM oJBL324 primer, 5 μl 10 μM oJBL529 primer, 0.5 µl SYBr green 100×, and water to 100 μl; run parameters: 3 min at 95 °C, followed by cycling with 20 s at 95 °C, 20 s at 65 °C and 50 s at 72 °C. To avoid overamplification, the reactions are tracked by qPCR and stopped at or below the inflection point. Ten PCR cycles are typically sufficient to get high-concentration libraries. PCR2 products are purified by 1× Ampure XP beads. Ten percent of the PCR2 product then serves as template for an indexing PCR3: same conditions as above, with primers oJBL076 (P5) and custom-indexed P7 (for example, oJBL530-533). Typically, four to six cycles are sufficient for indexing. Final libraries are purified by 1× Ampure XP beads (633 bp, amplicon: PCR3_mBC_10x_pdT_scQer.gbk on GitHub).

Sequencing of the mBC libraries follows the following structure: read1: primer standard Illumina Truseq read1, ≥28 cycles (cell barcode, UMI), index1: custom oJBL534, 6–10 cycles (sample index), read2: custom oJBL334, ≥15 cycles (mBC).

### Benchmarking and optimization via promoter series in human cell lines

#### Cloning and subassembly of dual-RNA reporter promoter series

To generate the dual-RNA reporter plasmid libraries, we first created a barcoded ‘cloning dock’ plasmid, with restriction sites and homology regions to various cassettes enabling modular addition of (1) Tornado^34^ RNAs cargos, (2) CRE libraries and (3) reporter mRNAs. To generate the cloning dock, plasmid p001 containing a piggyBac transposon backbone^95^ was digested with XbaI and HpaI (NEB) and the backbone product purified by agarose gel extraction (Zymoclean Gel DNA recovery kit, Zymo Research). To generate the cloning dock insert, a green fluorescent protein (GFP) fragment with barcoded 3′ UTR was amplified from plasmid pSGR017 with oJBL315 + oJBL316 (all primers and oligos are listed in Supplementary Data 11) and the resulting product gel was purified by PAGE. The barcoded 3′ UTR was combined with gene block gJBL008 with the piggyBac backbone by isothermal assembly (HiFi NEBuilder, NEB), the resulting plasmid, p022, was electroporated in *Escherichia coli* (NEB, C3020), and the full complexity of the was library maintained. Throughout, constructs were confirmed by colony PCR and Sanger sequencing of multiple clones.

We then added a barcode and capture sequence to the Tornado RNA plasmid pAV-U6 + 27-Tornado-Broccoli plasmid^34^ (Addgene #124360). The Tornado plasmid was digested with NotI and SacII (NEB) and the backbone purified by agarose gel extraction. A barcoded insert fragment was generated by PCR using the pAV-U6 + 27-Tornado-Broccoli plasmid as template and primers oJBL220 + oJBL291. The barcoded insert was assembled with the purified digested Tornado backbone and gene fragment gJBL007 by isothermal assembly and electroporated in *E. coli* (NEB, C3020), maintaining the full complexity of the library. The resulting plasmid, p019, contained the oBC with capture sequence 1 (CS1) cargo inserted in the Tornado cassette. Plasmids p019 was then digested with BamHI and XhoI (NEB) and p022 with BsbI, with the insert and backbone, respectively, purified by agarose gel extraction. The components were combined by isothermal assembly to generate plasmid library p025, which was electroporated in *E. coli*, maintaining complexity. Plasmid p025 contains the two barcodes (oBC and mBC) separated by 344 bp and is the starting point to clone scQers (Supplementary Fig. 2).

To construct five libraries (one per promoter in the series, see below), p025 was separately bottlenecked to an estimated 300 clones five separate times, and the oBC and mBC were subassembled from the separate pools. Briefly, amplicons were generated from the bottlenecked p025 as template, and using primers oJBL345 and oJBL337–oJBL341 (indexed primer, one per library). Reactions were carried out in 50 µl volume with 20 ng input plasmid template (25 µl polymerase master mix, 2.5 µl 10 µM oJBL345, 2.5 µl 10 µM indexed primer oJBL337–oJBL341, 0.25 µl 100× SYBr green, and water to 50 µl) using Kapa HiFi PCR master mix (Roche) with PCR conditions: 95 °C 3 min, cycling with 98 °C 20 s, 60° C 20 s and 72 °C 30 s. Reaction was tracked by qPCR and collected at the inflection point. Amplicons were purified by 1× Ampure.

Libraries were diluted to 2 nM on the basis of the TapeStation D1000 HS quantification, and sequenced on NextSeq500 with the custom primers: read 1 primer oJBL346 (oBC, 26 cycles), index 1 primer oJBL347 (library index, 6 cycles), read 2 primer oJBL348 (oBC reverse complement, 25 cycles) and index 2 primer oJBL349 (mBC reverse complement, 20 cycles).

Sequencing data were demultiplexed using bcl2fastq. Raw fastq files were processed first by trimming unnecessary cycles from the 3′ end (ten cycles from read 1, five cycles from read 2 and nine cycles from index 1) using seqtk^96^. Forward and reverse oBC reads were joined and error corrected with PEAR^97^ (options -v 16 -m 16 -n 16 -t 16). Using custom Python and R scripts, assembled oBC reads were combined with mBC reads, and oBC–mBC pairs were counted. The read count distribution displayed a clear bimodal distribution suggesting a saturated library, and oBC–mBC pairs with >500 reads were retained as valid. To further restrict the list of oBC–mBC pairs unique across the five bottlenecked libraries, all oBC–mBC pairs were combined, and any pair containing an oBC or mBC appearing more than once (either within a library or across different libraries) was discarded to avoid mapping conflicts in the analysis of single-cell reporter data (amounting to 24% of high-read-count pairs), leaving 1,122 unique oBC–mBC pairs across the five libraries (number of oBC–mBC pairs per library ranging from 139 to 306, with a median of 205).

Finally, each bottlenecked p025 library described above was digested with BglII, purified by 1× Ampure and digested with EcoRI (NEB), and the resulting backbone was purified by agarose gel extraction. Inserts composed of various promoters with puromycin cassette and GFP linked by a P2A element were generated as follows. For the human EEF1A1 promoter (including the first intron), minimal promoter and promoterless cassette, primers oJBL254 + oJBL314 were used to amplify respective constructs from plasmids pSGR017, pSGR018 and pSGR019 respectively, yielding a promoter puromycin-P2A-GFP fragment. For the human UBC promoter (including the first intron), puromycin-P2A-GFP fragment was obtained by amplifying from pSGR017 with primers oJBL254 + oJBL392, and the promoter fragment was amplified from plasmid pB-rtTA with primers oJBL393 + oJBL394. For the mouse Pgk1 promoter (no intron), puromycin-P2A-GFP fragment was obtained by amplifying from pSGR017 with primers oJBL254 + oJBL392, and the promoter fragment was amplified from plasmid PGK1p-Cys4-pA with primers oJBL395 + oJBL396. Promoter sequences are listed in Supplementary Data 1. All fragments were gel purified, combined with their respective digested bottlenecked p025 backbones and electroporated, resulting in five dual-RNA barcode reporter plasmid libraries, one for each promoter: p029 promoterless (noP), p027 minimal promoter (minP), p042 PGK1, p041 UbC and p028 EEF1A1. Given the a priori subassembly of mBC–oBC pairs for the starting bottlenecked plasmids, and the fact that each library above was assembled separately, each promoter was associated with a list of pairs of oBC and mBC, enabling downstream quantification in a single-cell context.

Plasmid libraries were purified by midiprep (Zymo Research), concentrated by isopropanol precipitation, and pooled at a 1:1 ratio by mass. This pooled library of the five promoters was used for both the benchmarking experiment in cell lines (Fig. 2a) and was also spiked in the developmental CRE experiment in mES cells (Fig. 3b).

#### Cell culture, transfection, bottlenecking and collection

K562 cells (CCL-243, ATCC) were grown in RPMI 1640 medium (Thermo Fisher, cat. no. 11875119), supplemented with 10% FBS (Fisher Scientific, Cytiva HyClone fetal bovine serum, cat. no. SH3039603) and 1× penicillin/streptomycin (Thermo Fisher, cat. no. 15140122). HepG2 (HB-8065, ATCC) and HEK293T (CRL-3216, ATCC) cells were grown in Dulbecco’s modified Eagle medium (DMEM; Thermo Fisher, cat. no. 10313021) with 10% FBS and 1× penicillin/streptomycin. Cells were kept at 37 °C and 5% CO_2_, and passaged every 2 days (K562, HEK293T) or when cells reached confluency (HepG2, typically every 3 days). For clonal expansion, we waited for near confluence from 12-well plates (1–2 weeks) before passaging.

All cells were transfected in mid-exponential phase. K562 cells were transfected using MaxCyte electroporation following manufacturer’s protocol (1.5 M cells, with 15 µg reporter scQers promoter plasmid mix (see above), 0.5 µg super PiggyBac transposase (SBI) in 50 µl volume). Two replicates of 1 M of HepG2 and HEK293T cells were transfected using Lipofectamine 2000 (Thermo Fisher, cat. no. 11668030, Gibco Opti-MEM cat. no. 31985) with 4 µg of reporter plasmid mix and 0.2 µg of super PiggyBac transposase (SBI). Medium was changed the next day, and cells passaged as usual thereafter. After 5 days, cells were put on puromycin selection (Gibco, cat. no. A1113803, concentration 2 µg ml^−1^) and grown for an additional 10 days to allow complete dilution of nonintegrated plasmids. After >15 days of growth post transfection, populations from each cell line were bottlenecked to an estimated 250 and 500 starting clones, and expanded to large populations. Notably, HepG2 cells displayed less robust growth at low densities, and required longer time for expansion, suggesting an effectively more severe bottleneck, in line with inferred clonal population properties (fewer final clones; Extended Data Fig. 3a,b).

The bulk versus single-cell quantification experiment (Fig. 2) was performed in two replicates. The first replicate (replicate A) with populations bottlenecked at an expected 250 clones, and the second replicate (replicate B) with populations bottlenecked at an expected 500 clones. For each replicate, at the same time, cells from each line were (1) collected separately and methanol fixed for bulk quantification and (2) prepared as single-cell suspension (Supplementary Fig. 9), hand mixed at an expected 1:1:1 ratio and profiled for single-cell transcriptomics. Briefly, for the bulk methanol fixation, K562 cells (and HEK293T and HepG2 cells following lifting off plate with 0.05% trypsin) were washed once with ice-cold phospate-buffered saline (PBS), and resuspended in 80% ice-cold methanol, to a concentration of 1 M cells ml^−1^, and placed at −80 °C until further processing. For single-cell processing, cells were washed twice with PBS + bovine serum albumin (BSA) (0.04%) and diluted to 1,000 cells µl^−1^. Cell dilutions were mixed at estimated equal proportion and loaded to expected 10,000 recovered cells total on the 10x Chromium platform following the manufacturer’s protocol (CG000205 Rev D, Single Cell 3′ v3.1 with feature barcoding, 10x Genomics), as one lane per replicate (two lanes total). Replicate B showed some evidence of a partial wetting failure but otherwise displayed a good emulsion.

#### Bulk MPRA library preparation

Genomic DNA was extracted from methanol fixed cells using the DNeasy kit (Qiagen), and RNA was extracted from cells using TRIzol LS (Thermo Fisher), following the manufacturer’s instructions in both cases. MPRA amplicon libraries from DNA were generated in two steps of PCR amplification with Kapa HiFi (Roche). A total of 0.5–1 µg of genomic DNA input was used. For low-cycle number PCR1, gDNA was mixed with 50 µl 2× Kapa HiFi master mix, 5 μl 10 μM oJBL039, 5 μl 10 μM oJBL358 and water to 100 μl. Cycling parameters: 1 min at 95 °C, and four cycles of 20 s at 98 °C, 20 s at 60 °C and 30 s at 72 °C, followed by 4 °C hold. Primer oJBL358 contains ten random Ns to serve as a pseudo-UMI (hereafter referred to as UMIs for brevity) to correct for PCR jackpotting. Reactions were cleaned up with Ampure XP beads at 1×, and eluted in 20 μl of 10 mM Tris 8. Illumina adapters and sequencing indices were appended through PCR2, with 4 μl of the eluate from PCR1 taken as input, and 25 μl 2× Kapa HiFi master mix, 0.25 μl 100× SYBr green, 2.5 μl 10 μM oJBL077, 2.5 μl 10 μM indexed primers (oJBL359–oJBL364), and water to 50 μl. Libraries were amplified with tracking by qPCR with 1 min at 95 °C, and cycles up to the qPCR inflection point with 20 s at 98 °C, 20 s at 60 °C and 30 s at 72 °C. Libraries were then cleaned up with Ampure XP beads at 1×.

Amplicon libraries for RNA were obtained by first DNase-treating RNA (5 μg RNA, 2 μl TURBO DNase (Thermo Fisher), 2 μl 10× buffer, and water to 20 μl, incubated at 37 °C for 30 min, cleaned up with RNA Clean & Concentrator (Zymo Research) and eluted in 11 μl Tris 7 10 mM). One microgram of DNase-treated RNA was then taken to reverse transcription. Briefly, 2 μl (500 ng μl^−1^) RNA was mixed with 2 μl 1 μM oJBL358, incubated at 65 °C for 5 min and placed on ice. Fifteen microliters of reverse transcription master mix was then added (4 μl 5× FS buffer, 1 μl 0.1 M dithiothreitol, 1 μl 10 mM dNTP mix, 8 μl water and 1 μl SSIII (Thermo Fisher)), and the reaction was incubated at 55 °C for 60 min, followed by 70 °C for 15 min. Half of the reverse transcription reaction was then directly amplified for PCR1 (37.5 2× Kapa HiFi master mix, 3.75 μl oJBL039 10 μM, 3.75 μl oJBL077 10 μM, and water to 75 μl), with cycling parameters of 1 min at 95 °C, and four cycles of 20 s at 98 °C, 20 s at 60 °C and 30 s at 72 °C, followed by 4 °C hold. Reactions were cleaned up with Ampure XP beads at 1×, and eluted in 20 μl of 10 mM Tris 8. PCR2 proceeded as for libraries prepared from genomic DNA, with oJBL077 and indexing primers (oJBL365, oJBL366 and oJBL437–oJBL440), and reactions were stopped at inflexion point from qPCR tracking. Libraries were then cleaned up with Ampure XP beads at 1×.

Final libraries were quantified with Qubit dsDNA HS (Thermo Fisher), diluted to 3 nM, run on TapeStation D1000 HS (Agilent) for final quality assessment, and adjusted to final 2 nM on the basis of the TapeStation quantification. Libraries were pooled and paired-end sequenced on NextSeq500 with the following primers and cycle numbers: read1 (mBC forward): 28 cycles, primer oJBL369; index1 (UMI): 19 cycles, primer oJBL435; read2 (mBC reverse): 19 cycles, primer oJBL371; index2 (sample index): 10 cycles, primer oJBL370.

#### Bulk MPRA data processing and quantification

Sequencing data were demultiplexed using bcl2fastq. Raw fastq files were processed first by trimming unnecessary cycles from the 3′ end (13 cycles from read 1, 4 cycles from read 2 and 9 cycles from index 1) using seqtk^96^. Forward and reverse mBC reads were joined and error corrected with PEAR^97^ (options -v 15 -m 15 -n 15 -t 15). Using custom Python and R scripts, successfully assembled barcode reads were combined with UMI reads, mBC–UMI pairs were counted, and the read and UMI counts per mBC were determined. The read and UMI counts for the mBC present in the reporter pool (determined a priori; see section ‘Cloning and subassembly of dual-RNA reporter promoter series’ above) were collected for downstream analysis and comparison to single-cell quantification.

Expression for each mBC from the UMI counts table was computed as follows. First, the total UMI per sample (per cell line and replicate) to the mBC in our list was determined for both RNA- and DNA-derived libraries. Each mBC UMI count was then normalized by the summed of counts in its respective sample type (DNA and RNA). The normalized RNA UMI count was then divided by the normalized DNA UMI count, to generate the bulk MPRA-derived estimate of expression per mBC.

#### Single-cell reporter data processing

Four different components are needed to perform reporter quantification using our approach: (1) a triplet map connecting CREs with oBC and mBC sequences, (2) single-cell gene expression UMI counts, (3) single-cell oBC UMI counts and (4) single-cell mBC UMI counts. For this promoter series experiment, the triplet CRE–oBC–mBC map was described above. We briefly describe below how the count data are obtained for the gene expression and barcoded RNAs. In each case, the output is a count table of the form (cell barcode, gene or barcode, and UMI count).

##### Gene expression libraries

Data were converted to fastq using bcl2fastq, and fastqs were minimally processed (trimming read 1 to 28 cycles with seqtk, files renamed) to be compatible with cellranger (version 6.0.1, 10x Genomics), which was run using reference GRCh38-2020-A. Each CellRanger count output was processed with Seurat^98^. Briefly, cell barcodes were filtered to those with >700 gene expression RNA UMIs, and between 2% and 15% mitochondrial UMI fraction. This led to 5,787, 4,278 and 3,834 cell barcodes across the replicates A, B1, and B2. 10x data were normalized, scaled and clustered using standard commands (NormalizeData with LogNormalize method, finding 1,000 top variable features with FindVariableFeatures, scaling with ScaleData over all genes, RunPCA and retaining top 50 principal components (PCs) calculated on the identified variable features, FindNeighbors on the top PCs, FindClusters with 0.1 resolution, and RunUMAP with n.neighbors of 20 and using the top PCs as input features). The uniform manifold approximation and projection (UMAP) revealed three clear clusters (Fig. 2b and Extended Data Fig. 2a), hypothesized to correspond to the three cell lines profiled. Replicates B1 and B2 also displayed an intermediate cluster, probably as a result of the lane partial wetting failure, found to share marker genes from the neighboring clusters, which was excluded as plausibly composed of doublets. To confirm the cellular identity of each cluster, in addition to assessment from canonical marker genes (for example, HBG1/2 in K562, and ALB in HepG2), we compared the pseudobulked expression (mean across UMI counts for each gene) to bulk expression quantification in the three lines (as assessed from the average of stranded bulk RNA-seq ENCODE^99^ datasets in K562 and HepG2, and in HEK293T), finding unambiguous correspondence of each clusters to a single line (average log-transformed *R*^2^ = 0.72 for matches, versus 0.39 for nonmatch).

Following preliminary filtering described above, cell barcodes corresponding to putative doublets were further filtered by two stringent methods. First, each large cluster was further subclustered using the same method as above, revealing focal subclusters that shared marker genes from large neighboring clusters, and usually had nearly twofold more total RNA UMIs. Cell barcodes contained in these clusters were excluded as likely doublets. Second, scrublet^100^ was run on the filtered cell barcode set (>700 RNA UMIs, 2–15% mitochondrial RNAs), and a doublet score threshold of 0.25 was selected for filtration based on the separation of the bimodal peaks in the simulated score distribution. Cells either belonging to doublet subclusters or having a scrublet doublet score >0.25 (we observed high concordance between the two approaches) were filtered out. Finally, cells with anomalously high gene expression UMI (>4,000) or anomalously high multiplicity of reporter integration (>100, see below), also likely doublets, were removed, leaving 5,505 high-confidence cells for replicate A (K562: 2,184, HEK293T: 2,090, HepG2: 1,231), 3,533 for replicate B1 (K562: 1,303, HEK293T: 1,238, HepG2: 992) and 3,172 for replicate B2 (K562: 1,298, HEK293T: 1,056, HepG2: 818).

##### mBC libraries

Data were converted to fastq using bcl2fastq, and fastqs were minimally processed (trimming read 1 to 28 cycles and read 2 to 22 cycles with seqtk, files renamed) to be compatible with cellranger (version 6.0.1, 10x Genomics), which was run to perform error correction on cell barcodes. The resulting position sorted bam files were then parsed for the mBC reads as follows using a custom Python script: reads aligning to the reference genome or without either corrected cell barcode or UMI (tags CB and UB in the bam file) were discarded. Only reads with the exact expected 7 nt sequence (TCGACAA) downstream of the mBC (positions 16–22) were retained. A list of all UMIs corresponding to a cell barcode and mBC pair was stored, discarding chimeric UMIs (taken to be UMIs for which the proportion of reads associated to a given mBC versus all other mBC in the specified cell barcode falls below 0.2). mBCs composed of all Gs (empty read) were discarded. Importantly, the mBC UMI counts were error corrected as follows. For each given mBC and cell barcode, the Hamming distance between all UMIs was calculated, a graph was created by connecting UMIs with a Hamming distance ≤1, and the resulting the number of connected components in the graph was taken as the error-corrected UMI count for a given cell barcode–mBC pair. These error-corrected UMI counts were taken as the per-single-cell quantification of the reporter mRNA expression (see section ‘Quantification of expression in single-cell assay and comparison to bulk’ for a normalization strategy to correct for technical factors). Given that cell barcodes derived from capture sequence versus poly-dT reverse transcription primer are different on the 10x Genomics beads (bases 8 and 9 reverse complemented) on the same bead (and not error corrected by cellranger in our application), we converted the CS2 cell barcodes to their poly-dT counterparts to enable matching across the different libraries.

##### oBC libraries

oBC libraries were processed in an entirely analogous way to the strategy for mBC described above, with the following modifications: two sequencing runs were combined in a single fastq before processing, read 2 were trimmed to 23 cycles, and only reads with the GCTTTAA (constant region after the oBC) at positions 17 to 23 were retained. The number of UMIs per oBC per cell barcode was also taken as the error-corrected (1 Hamming distance) count and our measure of oBC expression in single cells (see section ‘Quantification of expression in single-cell assay and comparison to bulk’ for a normalization strategy to correct for gene expression UMIs). Similarly to the CS2 mBC data above, we again converted the CS1 cell barcode to poly-dT cell barcodes.

#### Quantification of expression in single-cell assay and comparison to bulk

To quantify reporter expression via our single-cell experiment, we first determined the set of valid oBC (present in our oBC–promoter–mBC subassembly table generated a priori) detected in each cell. As a tradeoff between specificity and sensitivity (see clonotype precision–recall analysis below), we selected a threshold of ≥12 UMI (Fig. 2c) to deem a oBC as present for a given cell barcode. The UMI counts for valid mBC–cell barcode pairs were then joined to the detected oBC in all valid cell barcodes by using the predetermined oBC–mBC (uniquely matchable) association table. In cell barcode–oBC combinations for which there were no detected mBC UMI, a value of 0 was taken (detection of reporter integration from oBC but no captured reporter mRNA). Importantly, while not detected, given our dual RNA strategy, this represents a ‘true’ zero and contributes to our measurement of expression. mBC UMI counts were normalized by the number of gene expression UMI (from the full transcriptome GEx libraries) detected in each cell, that is, (mBC UMI)/(GEx UMI) × mean(GEx UMI), where the scaling with the mean gene expression UMI across all cells served to maintain an intuitive unit in the data. Normalization by simple scaling by gene expression UMI was performed as the mBC UMI counts were correlated (*R*^2^ of log-transformed values, 0.09) with gene expression UMI with a slope close to 1 (least square fit on log-transformed data, slope 0.93). We find in both our comparison to bulk data and our clonal analysis (see below) that direct normalization of mBC by GEx slightly improves the precision of the expression measurement. To quantify single-cell expression for each mBC (Fig. 2g), we then directly averaged the normalized mBC UMI counts across all cells with a detected associated oBC.

The averaged normalized mBC UMI described above was directly compared to the bulk expression quantification (from bulk MPRA) (Fig. 2e and Extended Data Fig. 2d). In these analyses, we only include well-represented barcodes in the comparisons to focus attention on technical noise resulting from the two methods and not noise from sparse sampling of rare barcodes (mBC with 5 or more cells with oBC detected integrations, at least 1 mBC UMI captured across all integrations, and at least 100 DNA UMI from the bulk quantification).

For quantification without conditioning on oBC detection (Extended Data Fig. 2h), the average normalized mBC UMI across all cells with any captured counts was taken. Including an additional step to filter possible chimeric amplicons (removing events for which the number of reads equaled the number of UMIs, unlikely in a saturated library) did not substantially improve performance without oBC detection.

In addition to the accuracy comparison to the bulk quantification, we also directly assessed the number of incorrectly detected mBC (mBC UMI count >0, but not detected as determined by absence of the associated oBC (<12 oBC UMI) in the same cell) for the different promoters. We found the following proportions of valid (oBC matched) mBC detection events (mean proportion from replicates A and B1): no promoter: 60%, minimal promoter: 45.9%, UBCp: 51.4%, Pgk1p: 40.4%, EEF1A1p 10.5%. Spurious detections thus constituted a substantial, and sometimes dominant, proportion of events in all cases.

### Profiling developmental CREs in mEBs

#### Cell culture

##### mES cells

A low-passage-number monoclonal male BL6 (male WD44, ES-C57BL/6 gift from C. Disteche and C. Ware at University of Washington) mES cell line stably expressing dCas9-BFP-KRAB was used. Cells were grown on plates coated with gelatin (0.2%) (Sigma, cat. no. G1890) and cultured in DMEM (Thermo Fisher, cat. no. 10313021) supplemented with 15% FBS (Biowest, Premium bovine serum, cat. no. S1620), 1× MEM nonessential amino acids (Thermo Fisher, cat. no. 11140050), 1× GlutaMAX (Thermo Fisher, cat. no. 35050061), 10^−5^ β-mercaptoethanol and 10^−4^ leukemia inhibitory factor (Sigma-Aldrich, ESGRO Recombinant Mouse LIF Protein ESG1107), hereafter referred to serum + LIF medium where necessary, with daily medium changes (aspirate medium, replace with prewarmed medium) and transfer every 2 days (aspirate medium, wash with PBS (without Ca^2+^ and Mg^2+^), add 2.5 ml (for 10 cm plate) 0.05% trypsin, incubate 2 min at 37 °C, deactivate trypsin and triturate with 10 ml prewarmed medium, spin down 5 min at 300*g*, aspirate supernatant, resuspend in prewarmed medium and transfer to new gelatinized plate).

##### mEB induction and maintenance

Exponentially growing mES cells are lifted from the plate (aspirate serum + LIF medium, wash with PBS, add 2.5 ml (for 10 cm plate) 0.05% trypsin, incubate 2 min at 37 °C, deactivate trypsin and triturate to a single-cell suspension with 10 ml prewarmed medium). Cells are then counted and spun down (5 min at 300*g*). Supernatant is aspirated, and cells are resuspended to 2M ml^−1^ in CA medium (medium for EB induction: DMEM, 10% FBS, 1× MEM nonessential amino acids, 1× GlutaMAX and 10^−5^ β-mercaptoethanol). Cells are counted again, and density adjusted to 1M ml^−1^ with CA medium. Three milliliters (3M cells) is added to 12 ml of CA medium in 10 cm plates (suspension plates: nongelatinized, nonadherent). On the next day, plates are gently agitated to promote cell aggregation. Following induction, embryoid bodies (mEBs) are passaged every 2 days (no daily medium change). mEBs are collected using a serological pipette and transferred to a 50 ml conical tube (typically, three plates are pooled). Leftover mEBs on plates are recovered by a CA medium wash and pooled in the conical tube. mEBs are left to settle (initially up to 15–20 min, faster as the mEBs grow in size). Once mEBs have settled, medium is aspirated from the top, carefully avoiding disturbing the loose pellet. Fresh, prewarmed CA medium is then added to 15 ml per plate, and mEBs are redistributed to plates.

#### Construction of CRE series dual RNA reporter plasmid library

Doubly barcoded backbone p025 was recloned at higher complexity (~1M oBC–mBC pairs; see Supplementary Note 6 for details).

##### PCR cloning of putative developmental CREs and assembly in dual RNA plasmid

Putative CREs selected for profiling (see above) were cloned by PCR from mouse genomic DNA. A compromise amplicon size of 0.9 kb was taken as rough target size to balance testing large regions without overly compromising success rate. To increase specificity, a nested PCR approach was taken: a first unburdened PCR with selected primers (below), followed by a second nested PCR using primers with homology arm for cloning in the common backbone.

Outer primers for the first PCR (Supplementary Data 11) were selected by running Primer-BLAST^101^ with as PCR templates the 1,200 bp sequences for the putative CREs (350 bp symmetric extension on both sides of the ArchR called 500 bp ATAC peak window with bedtools^102^ slop, followed with bedtools getfasta to obtain sequences from mm10 genome) with the following run criteria: PCR product size 800–1,000 bp (forward primer between 0 and 200 bp and reverse primer between 1,000 and 1,200 bp), primer melting temperature: Min 57.0 (minimum) Opt 60.0 (optimal) Max 63.0 (maximum), Max Tm difference 3 (largest difference in melting temperature between the two primers), no intron junction preference, specificity check to *Mus musculus* (taxid: 1009). For certain CREs, Primer-BLAST did not return any specific result with these constraints. Constraints (on product size) were then sequentially relaxed to increase the search space, with ultimately requiring only that the product be at least 500 bp within the window. Five regions (Foxa2_chr2_13861, Sparc_chr11_7210, Lamb1_chr12_2182, Lamb1_chr12_2183 and Sox17_chr1_58) were still too repetitive for Primer-BLAST to return results but had nonrepeat sequences enabling manual primer selection. Two regions were too repetitive to find any primer pairs whatsoever and were thus not included in the screen (Sparc_chr11_7186 and Foxa2_chr2_13842). Overall, primers were ordered to PCR clone 209/2011 CREs from our initial selected set.

Inner primers for the second nested PCR were selected using batch primer3 (ref. ^103^) (nondefault options: GC clamp = 1, max poly-X = 4) using the first PCR product as a template (but allowing for at most 8 bp overlap between inner primers and the PCR1 product). Primer pairs leading the largest nested PCR product were selected and handles homologous to the backbone were added (forward: 5′accgcatcgatctcgagg[inner forward], reverse: 5′tcccaaagcagatgtagttgac[inner reverse]). Handles were added to the forward/reverse primer so that the orientation of the CRE relative to the promoter matched their relative orientation on the genome relative to the gene.

The first PCR was performed in 20 µl reactions with 40 ng of genomic DNA (collected from the mES cell line used (DNeasy, Qiagen) following the manufacturers’ instructions) with Kapa Robust (Roche) with following parameters: 95 °C 3 min; 40 cycles: 95 °C 15 s, 60 °C 20 s and 72 °C 1 min 40 s; final extension 72 °C 1 min 40 s; with individual reactions in separate wells of a 96-well plate with primers distributed using a 96-liquidator (Rainin). Products were cleaned up (1× Ampure XP beads) and visually checked on agarose gel (with >95% success rate as judged by presence of ~1-kb-sized band, possibly with nonspecific products), and eluted in 100 µl of 10 mM Tris 8. Then, 0.5 µl of the purified up PCR1 products was taken as template for the second nested PCR using the same conditions but with the inner primers. The resulting products were cleaned up (0.6× Ampure XP beads) and visually checked on agarose gel, showing a <2% failure rate and highly clean products (little nonspecific bands or smears). The products were quantified with a spectrophotometer (Nanodrop) and pooled to a 1:1 ratio by weight. This pool was used as insert for a pooled Gibson assembly as described below.

Before addition of the putative CRE PCR products, the minimal promoter GFP cassette (reporter mRNA) was inserted in the doubly barcoded backbone p025 digested with EcoRI and BglII (NEB) (Supplementary Fig. 2a) and maintained at highest clonal complexity upon transformation (electroporation without bottleneck) to generate plasmid library p043. The minP-GFP insert was generated by splice PCR (templates: minP fragment: amplification of p027 with primers oJBL314 + oJBL416; GFP fragment: amplification of p027 with primers oJBL254 + oJBL414) followed by gel extraction. Plasmid library p043 was then digested with NheI/MfeI, combined with the pooled PCR-amplified CREs via Gibson assembly, and transformed (electroporation) with a bottleneck via 100-fold dilution to an estimated complexity of ~50,000 clones (Supplementary Fig. 2a). The resulting plasmid library (p055) was then subjected to the final subassembly step to connect oBC to the CRE.

##### oBC–CRE subassembly

Given the length of the inserted CREs (~1 kb) and diversity of sequences, amplification of the region from minimal promoter to oBC was not a feasible strategy to subassemble oBC to CRE (~1.3 kb from minP to oBC). We thus relied on tagmentation followed by semi-specific PCR. Briefly, plasmid library p055 was tagmented with Tn5 (Illumina, Nextera Tagment DNA enzyme, cat. no. 15027916) at a concentration such that the expected fragment size would be larger than the oBC to minP distance (~1.3 kb), determined by a Tn5 titration curve experiment. Following tagmentation (5 µl 2× Tagmentation DNA buffer (Illumina, cat. no. 15027866), 0.4 µl Tn5 enzyme 1, 3.6 µl water, 1 µl 10 ng µl^−1^ plasmid library; 30 min at 37 °C), the tagmented plasmids were cleaned up (Zymo Clean and Concentrator, 3:1 binding buffer), eluted in 10 µl Tris 8 10 mM. One nanogram (1 µl of the elution) was amplified via semi-specific PCR with a Nextera primer with a P5 handle (oJBL512, binding to all P5 tagmentation events) and an oBC-specific upstream primer (oJBL502, binding to specific portion of the plasmid) in 25 µl (8.9 µl water, 12.5 µl 2× NEBNext master mix, 1.25 µl 10 µM oJBL502, 1.25 µl oJBL512, 1 µl tagmented plasmids and 0.1 µl 200× SYBR green) with the following conditions (gap fill: 72 °C for 5 min and 98 °C for 30 s, then 12 cycles of 98 °C for 10 s, 65 °C for 30 s and 72 °C for 1 min). As controls for the nonspecific product size distribution, the tagmented plasmids were also amplified with oJBL512 exclusively. Following purification (Zymo Clean and Concentrator), the amplified libraries were run on PAGE (6% Tris–borate–EDTA, 180 V, 30 min). As anticipated, the amplicons with primers oJBL502 + oJBL512 (semi-specific products) displayed reduced size distribution compared to oJBL512 alone amplified (nonspecific) products, with most oJBL512 exclusive amplicons >1.2 kb. Semi-specific oJBL502 + oJBL512 products between 450 bp and 800 bp were size selected on the PAGE gel, purified (minimum size size from CRE ~75 bp) and sequenced (read 1: CRE sequence, Illumina Nextera primer (no custom), 34 cycles; index 1: P7-idx, primer oJBL432 15 cycles; read 2: oBC, primer oJBL433, 30 cycles).

Following demultiplexing (from the P7 index), the sequencing data were processed by first aligning read 1 (mapping to CRE) using bowtie2 (v2.4.4)^104^ using option ‘-k 2’ to report multi-mapping regions (some of our CRE segments overlapped given the proximity of the called peaks and extension from 500 bp to ~1 kb tested regions). The resulting alignment sam file was then sorted, converted to bam using SAMtools^105^, and merged with the oBC (read 2) using custom scripts into a file storing the oBC, CRE identity of the mapping, position and strand of aligned read within the CRE. Total read counts to each oBC–CRE pair were then summed up with custom scripts, retaining information about distribution of alignment positions and strand within the CRE for downstream processing.

The piled-up count data on oBC–CRE pairs were then filtered to identify bona fide, unique pairs. First, pairs with median mapping position outside the expected range from the size selection step (<30 bp and >300 bp) and mapping on the incorrect strand were filtered out. Then, the proportion of oBC reads mapping to any given CRE was calculated across oBC–CRE pairs, and only pairs with >95% of oBC reads mapping to a unique CRE were retained. The read count distribution across all oBC–CRE pairs was bimodal suggesting a saturated library, and only pairs with >30 reads (separating the two modes) were retained. Finally, pairs with anomalously small or large mapping position dispersal (90th to 10th percentile difference mapping positional spread <30 bp or >300 bp) were filtered out. We note that the positional filters enabled unambiguous discrimination between different but overlapping CREs (given that in all cases one of the CRE would have out of range mapping positions compared to the expected size from the amplicon library). Two elements (*Gata4*:chr14_5749 and *Txndc12*:chr4_7975) shared a short identical sequence complicating the mapping, and were treated separately to not confound the fraction of oBC reads mapping to a given CRE. Following these filtering steps, we were left with 43.6k valid oBC–CRE pairs.

##### Final oBC–CRE–mBC triplet table

These oBC–CRE subassembled pairs were then linked with the previously determined oBC–mBC pairs from the starting plasmid library p025. Briefly, oBCs (from final oBC–CRE pairs) were joined to mBC via valid oBC–mBC pairs (restricting to the uniquely mapped pairs). The resulting valid triplets oBC–CRE–mBC were then joined with the oBC–promoter–mBC triplets of the exogenous promoter library (experiment from Fig. 2a), and any oBCs or mBCs appearing twice in both libraries were removed from the final triplet list. The final number of valid oBC–CRE–mBC triplets was 33,000, with a median of 145 valid mBC–oBC pairs per CRE. The resulting triplet map was used to deconvolute single-cell data in reporter quantification. Through the cloning and subassembly process, 5 out of the attempted 209 CREs dropped out (<20 valid mBC–oBC pairs), and consequently could not be quantified (*Col1a1*:chr11_15306, *Col1a2*:chr6_65, *Cited2*:chr10_1265, *Txndc12*:chr4_7952 and *Btg1*:chr10_9570).

#### Experimental details of pooled screen for CRE in mEBs

##### Transfection, cell culture and bottlenecking

Low-passage-number mES cells were expanded in serum + LIF medium on gelatin-coated plates as described above (passaged every 2 days, medium change every day) on 10 cm plates. Cells were transfected using Lipofectamine 2000 (Thermo Fisher Scientific) using reverse transfection. Briefly, cells washed with 1× PBS, and lifted by adding 2.5 ml per 10 cm plate of trypsin 0.05% (Gibco). Following incubation at 37 °C for 5 min, cells were triturated with an added 7.5 ml of medium, spun down at 300*g* for 5 min and resuspended by pipetting at an estimated 1.5M ml^−1^ to obtain a single-cell suspension. Following straining (40 µm), cells were counted and diluted to 0.5M ml^−1^ with medium. Concurrently, the Lipofectamine + opti-MEM (12 µl Lipofectamine + 238 µl opti-MEM) and the opti-MEM + DNA (240.4 µl opti-MEM + 4 µl 50 ng µl^−1^ transposase + 5.6 µl transposon mix containing 3.8 µg of plasmid, see below) were separately prepared and mixed by pipetting. The 500 µl Lipofectamine + DNA + opti-MEM mix was then added to a gelatin-coated plate, 1M cells (2 ml) from the single-cell suspension was added to the plate, and gently mixed. No transposase and no DNA controls were included. The transfected transposon was an uneven mix of three components (too boost MOI, see below): (1) 89% of the p055 oBC-CRE-minP-GFP-mBC library, (2) 10% of the oBC-promoter-puromycin-GFP-mBC series (same as for experiment in cell lines, Fig. 2a) and (3) 1% of the EEF1A1p-mCherry plasmid (p060, see below). Two biological replicates were transfected in parallel, one with the hyPBase plasmid^41^, and one with super PiggyBac (SBI). We did not find substantial difference in MOI in the two replicates (Extended Data Fig. 5c, replicate A versus B).

Transfected cells were passaged and expanded to allow for integration and unintegrated plasmid dilution. Five days post transfection, cells were split with a portion selected on puromycin (2 µg ml^−1^) and another portion remaining unselected. After 5 days on puromycin, cells from no DNA controls and no transposase controls were dead. While a large proportion of cells in samples with integrated cargos samples died, the puromycin-resistant population was expanded for 2 weeks post transfection to ensure complete dilution of the unintegrated plasmids (maintained on puromycin).

The two replicates were induced to form mEBs in CA medium (no puromycin) on suspension plates as described above (day 0, 14 days post transfection), starting with 24M cells per replicate (eight 10 cm plates with 3M cells each in 15 ml of CA medium). Replicate A was the sample transfected with hyPBase (and selected on puro), replicate B the sample transfected with the SBI super PiggyBac. Following induction, mEBs were passaged every 2 days, with sampling 5–10% of EBs at each time point for bulk MPRA (for collection, mEBs were pelleted at 5 min at 300*g*, medium aspirated, fixed with ice-cold 80% methanol, and stored at −80 °C until processing).

Still in the mESC growth period, at the 12 day time point post transfection, a subset of expanded cells from replicate B were sorted by fluorescence-activated cell sorting (FACS) for mCherry signal, and plated on an MEF monolayer (Thermo Fisher, CF1 Mouse Embryonic Fibroblasts, MitC-treated, cat. no. A34958, plated at 0.4M cells per well) in the wells of six-well plate at approximately 1,000 cells per well for bottlenecking. Following colony expansion for 4 days with daily medium change, colonies were lifted as follows: two washes with 1× PBS, addition of 750 µl collagenase type IV (0.1%, Stemcell Technologies, cat. no. 07909), 8 min incubation at 37 °C and aspiration of lifted colonies by pipetting. The collagenase-treated colonies on MEFs were then gently washed twice with 1 ml of serum + LIF medium added dropwise to recover additional colonies, and pooled with the previous ones. Lifted colonies were then spun down (400*g*, 5 min), medium aspirated, trypsin treated to single-cell suspension (250 µl 0.05% trypsin used to mix the pellet, incubated 3 min at 37 °C, inactivated and triturated with 2 ml of fresh medium, and plated on gelatin-coated plates for expansion. Counting colonies suggested about half, or 500 clones, were obtained in this way. Following expansion for 8 days, mEB induction with 24M cells (eight 10 cm plates with 3M cells each) was initiated as above. mEBs were passaged every 2 days, with sampling 5–10% of EBs at each time point for bulk MPRA as before. The bottlenecked replicate was termed 2B.

##### End-point processing and single-cell sequencing

For both nonbottlenecked and bottlenecked experiments above, mEBs were processed at the 3 weeks end-point as follows (for each replicate): two suspension 10 cm plates of mEBs were pooled into a 50 ml conical left to settle. Medium was aspirated, and mEBs were washed twice with 1× PBS, resuspended in 3 ml 1× PBS in the second wash, and split in two 1.5 ml aliquots in 2 ml tubes. PBS was aspirated from the tubes, and 500 µl of trypsin 0.25% was added per tube. Tubes were then agitated on a thermomixer at 37 °C and 650 rpm for 4 min. Cells were then gently dissociated by pipetting ten times, and placed back on the thermomixer for 2 min. One milliliter of medium was then added per sample and pipetted to obtain a single-cell suspensions, the two samples were combined in a 15 ml conical, after passing them through a 100 µm strainer. The strained single-cell suspension was counted, and cells were spun down (300*g*, 5 min), resuspended to 4M ml^−1^, and taken to FACS to obtain a clean single-cell suspension (typical gating strategy shown in Supplementary Fig. 1). More than 600,000 cells were then FACS sorted (in <50 min) in prewarmed medium to ensure the single-cell nature of the suspension (no gating on fluorescence, only on forward and side scatter) before generating the emulsions for scRNA-seq. Sorted cells were then spun down at 400*g* at 4 °C for 5 min, the medium was gently aspirated, cells were resuspended to an expected 2.5M cells ml^−1^ (based on FACS sort event counts) in ice-cold 1× PBS + 0.04% BSA, cells were further counted and volume was adjusted to have 1,200 cells µl^−1^ with ice-cold PBS + BSA.

Single-cell suspensions in PBS + BSA were taken as the starting point for the 10x Genomics protocol (v3.1 with feature barcoding). Emulsion and reverse transcription were performed per the manufacturer’s instruction. Given prior empirical experience with mEBs processing, each 10x lane was slightly overloaded (by an additional 20%) to approach the expected recovery of 10,000 cells per lane. Each replicate was profiled with two lanes of 10x, for a total of six lanes.

#### Single-cell reporter data processing

Processing proceeded in a similar way as described for experiment in cell lines. See Supplementary Note 6 for details.

##### Quantification of activity and specificity of CREs and statistical tests

The following stringent tests were performed to identify active and specific CREs. Each CRE and biological replicate was considered separately.

To assess activity, all integration events (oBC UMI >10) for the CRE considered were identified, and the total number of such integration events for the CRE recorded. A total of 10^4^ bootstrap resamplings (random sampling with replacement) of the integration events were then performed. In parallel, sampling with replacement of integration events (same number sampled as the CRE considered to control for difference in representation) from both basal promoter controls (minimal and no promoters) was performed. For each bootstrap sampling, the average normalized mBC UMI counts (see above), stratified by cell-type clusters (Seurat identified; Extended Data Fig. 4a), were determined both for the CRE and the basal promoters. The maximum expression cluster identity and expression level in that cluster was stored. Mean expression of the reporter without stratification by cluster identity was also obtained (over all bootstrap resampled integration events irrespective of cell types). Following bootstrapping, an empirical *P* value was determined as follows: the null distribution was taken as the maximum cluster expressions across all bootstrap samplings of the two basal promoters. The empirical *P* value of expression for the CRE considered to have activity in excess of the basal control (activity *P* value) was taken as the probability that maximum cluster bootstrap CRE expression was below that of the basal controls, averaged over all bootstrap sampling for the basal control events (effectively corresponding to a rank-sum test). Empirical activity *P* values (over all CREs within a replicate) were Benjamini–Hochberg corrected to obtain an FDR. Corrected empirical *P* value without stratification over clusters was similarly performed (mean probability that expression from the CRE over all integration was below that of basal control null bootstrap values). To identify active CREs, we considered elements with either per-cluster maximum expression FDR <10% in all three replicates and/or all cells expression FDR <1% (higher statistical power from more integration events) in all three replicates. A total of 58/204 CREs passed these stringent criteria and were considered active in excess of our basal expression controls.

To assess CRE specificity, a similar approach was taken, but instead of performing comparison to basal promoters, comparisons were performed to datasets with permuted cell cluster identities. For each CRE, 10^4^ repeats were performed, a bootstrapped resampled (no cluster identity permutation) set of integration events was generated, and the fold change in reporter expression (average normalized mBC UMI) between the maximum expression cluster and the rest of cells was computed. The corresponding quantity, but for a cluster-identity permuted sampling, was also performed for each sampling. The specificity empirical *P* value for each CRE was taken as the average (over resamplings) probability that the cluster permuted fold changes in expression (null distribution over all permutations) was higher than the nonpermuted one. As before, these empirical *P* values were Benjamini–Hochberg corrected (over all CREs, separately for different biological replicates). CREs that were identified as active were further marked as specific if in all biological replicates, the reporter expression fold change (maximum cluster versus all other cells) was >5 and the permutation-derived FDR <10%, leading to 9/58 elements.

To systematically assess whether elements had pleiotropic activity (active in multiple cell types), we computed the fold change in expression in all pairs of clusters versus the rest of cells, storing the maximum fold-change value and specific cluster pair for each CRE and biological replicate. The median (across biological replicates) fold changes for pairs versus individual clusters were compared. Only a single CRE had a paired/single cluster fold change in excess of 3× was *Lamc1*:chr1_12189 (also elevated: 2.6× for *Foxa2*:chr2_13858, which displayed some activity in visceral endoderm in addition to parietal (Fig. 4e, second row); and 1.5× for *Sox2*:chr3_2007, which had some activity in epiblast cells (Fig. 3f)). Other elements showed no substantial excess activity in pairs over single clusters (95% percentile in pair/single fold changes was at 1.3× and 90% percentile at 1.1×). Permutation tests similar to above confirmed Lamc1 bifunctional activity were highly significant (nonpermuted fold change highest in all 10^3^ samplings), leading to a final set of 10/58 active CREs labeled as specific.

To summarize the function of individual CREs, the median activity (defined as the maximum cluster mean reporter expression) and specificity (defined as the fold change between maximum cluster mean reporter expression versus mean reporter expression in the rest of cells) across the three biological replicates was determined (shown in Fig. 4a).

Some elements were active and/or specific in only a subset of replicates (those marked in Extended Data Fig. 7b, for example, *Bend5*:chr4_8174, *Foxa2*:chr2_13820, *Sox17*:chr1_77, *Bend5*:chr4_8179, *Lama1*:chr17_7791 and *Lamc1*:chr1_12185). These are likely candidates for active elements (falling below our limit of detection possibly because too few integration events were captured due to uneven CRE representation) but were not retained to maintain stringency in our downstream analyses. Quantification summary can be found in Supplementary Data 5.

Pseudobulk expression in separate cell types (for example, Extended Data Fig. 5b) was determined as the average normalized mBC UMI counts over all cells with detected reporters belonging to GEx clusters identified and annotated in Extended Data Fig. 4a.

### Statistics and reproducibility

No statistical method was used to predetermine sample size. The experiments were not randomized. The Investigators were not blinded to allocation during experiments and outcome assessment.

Benchmarking experiments and optimization experiments in cell lines were carried out in two independent replicates, with reproducible results. Experiments in mEBs were carried out in biological triplicates, with reproducible results. Bulk MPRA experiments comparing Pol III circular and linear barcodes were carried out in independent biological duplicates, with reproducible results. Singleton validation experiment was performed as a single experiment (with one independent differentiation for the eight tested constructs). Multiple EBs within each condition, however, showed expected behavior (cell-type-specific expression).

Detailed statistical tests and quantitative treatment of data are otherwise described at relevant sections in Methods and Supplementary Note 6.

No data were excluded from the analyses apart from a single sample/time point from bulk MPRA in mEBs (day 20, replicate 2B1, first round of experiment). This library had been generated from a lower amount of starting RNA (yield from that extraction had been lower). Inspection of read counts to basal promoters showed drastically higher apparent activity compared to other samples, suggesting that signal in the RNA originated from trace contaminant genomic DNA, which had a disproportionate weight in that sample due to the low starting RNA quality. This sample was thus excluded from downstream analysis.

### Reporting summary

Further information on research design is available in the Nature Portfolio Reporting Summary linked to this article.

## Online content

Any methods, additional references, Nature Portfolio reporting summaries, source data, extended data, supplementary information, acknowledgements, peer review information; details of author contributions and competing interests; and statements of data and code availability are available at 10.1038/s41592-024-02260-3.

### Supplementary information

Supplementary Information Supplementary Notes 1–6 and Figs. 1–9.

Reporting Summary

Supplementary Data 1 Sequences of exogenous promoters (Fig. 2a) used for benchmarking and as internal standards as well as tested accessible chromatin regions derived from mEB scATAC data (Fig. 3a).

Supplementary Data 2 High-confidence clonotypes and clonotype-assigned cells, human cell lines (Fig. 2g and Extended Data Fig. 3).

Supplementary Data 3 Quantification of variability in activity of integrated reporters [+/-U6/oBC]×[+/-cHS4] (Supplementary Fig. 1).

Supplementary Data 4 Positions of SCR elements (Extended Data Fig. 6) and other literature-selected CREs (Supplementary Fig. 3h,i).

Supplementary Data 5 Quantification of activity and specificity for all CREs measured with scQers (Fig. 4a).

Supplementary Data 6 High-confidence clonotypes and clonotype-assigned cells, mEBs (Supplementary Fig. 6).

Supplementary Data 7 Quantification of activity for all CREs from bulk MPRA time series (Extended Data Fig. 9).

Supplementary Data 8 Information on CREs with perturbed putative transcription factor binding sites (Extended Data Fig. 10 and Supplementary Figs. 2a and 7d).

Supplementary Data 9 scQer measured activity for paired, mutated and literature-selected CREs (Extended Data Fig. 10 and Supplementary Fig. 2).

Supplementary Data 10 Bulk MPRA quantification in mEBs testing different reporter architectures (Supplementary Fig. 8).

Supplementary Data 11 Oligos and plasmids used in this work.

Supplementary Data 12 Source data for Supplenentary Fig. 1.

Supplementary Data 13 Source data for Supplementary Fig. 2.

Supplementary Data 14 Source data for Supplementary Fig. 3.

Supplementary Data 15 Source data for Supplementary Fig. 4.

Supplementary Data 16 Source data for Supplementary Fig. 6.

Supplementary Data 17 Source data for Supplementary Fig. 7.

Supplementary Data 18 Source data for Supplementary Fig. 8.

### Source data

Source Data Fig. 2 Statistical source data.

Source Data Fig. 3 Statistical source data.

Source Data Fig. 4 Statistical source data.

Source Data Extended Data Fig. 1 Statistical source data.

Source Data Extended Data Fig. 2 Statistical source data.

Source Data Extended Data Fig. 3 Statistical source data.

Source Data Extended Data Fig. 4 Statistical source data.

Source Data Extended Data Fig. 5 Statistical source data.

Source Data Extended Data Fig. 6 Statistical source data.

Source Data Extended Data Fig. 7 Statistical source data.

Source Data Extended Data Fig. 8 Statistical source data.

Source Data Extended Data Fig. 9 Statistical source data.

Source Data Extended Data Fig. 10 Statistical source data.

## Data Availability

Raw sequencing data and processed files generated in this study have been deposited to GEO, with accession number GSE217690 and to the IGVF data portal (accession IGVFDS7801YPEU, IGVFDS2774OLAH and IGVFDS2622CKLA). Published data used: transcription factor binding data (Uniprobe^106^: *Gata4* (ref. ^107^) UP01372, *Sox17* (ref. ^108^) UP00014, *Foxa2* (ref. ^108^) UP00073), mouse embryo in vivo scRNA-seq^61^ (obtained from R library: ‘MouseGastrulationData’) and scATAC-seq^53^ (GEO: GSE205117). Promoter control scQer libraries (p027, p028, p029, p041 and p042) and cloning intermediate libraries with preassociated list of oBC–mBC (p025 and p043) have been deposited to Addgene (respective identifiers 1000000239, 194097 and 194098; https://www.addgene.org/pooled-library/shendure-scqers/). Source data are provided with this paper.

## Code availability

Code and scripts used for analyses have been deposited on GitHub at https://github.com/shendurelab/scQers (ref. ^94^), together with the maps of plasmids and custom sequencing amplicons structures used in this work.
