# Supplementary material for: Multiplex profiling of developmental cis-regulatory elements with quantitative single-cell expression reporters
Source: Nat Methods. 2024 May 9;21(6):983–93. doi: 10.1038/s41592-024-02260-3 (PMC11166576; doi:10.1038/s41592-024-02260-3)
Supplement: Supplementary file 1 — Supplementary Notes 1–6 and Figs. 1–9. [file 41592_2024_2260_MOESM1_ESM.pdf]

nature methods

Article

https://doi.org/10.1038/s41592-024-02260-3

Multiplex profiling of developmental *cis*-regulatory elements with quantitative single-cell expression reporters

In the format provided by the authors and unedited

SUPPLEMENTARY INFORMATION

Supplementary Figures (p. 2 to p. 16)

Supp. Fig. 1: Comparison of cHS4 and Pol III U6/oBC cassette for insulating effects
Supp. Fig. 2: scQer library construction and oBC-CRE-mBC subassemblies
Supp. Fig. 3: Quality control metrics for applications of scQer experiment
Supp. Fig. 4: Singleton validation experiment of cell type-specific CREs
Supp. Fig. 5: Structured illumination images of mEBs with singleton scQer reporters
Supp. Fig. 6: Cell-type-specific CRE expression across clones to assess positional integration effects
Supp. Fig. 7: CRE features correlated to cell-type-specific activity
Supp. Fig. 8: Assessing the impact of different reporter architectures
Supp. Fig. 9: FACS gating strategy

Supplementary Note

Note 1 (p. 16): Systematic assessment of integration positional effects
Note 2 (p. 16): Comparing CRE activity and putatively associated gene inductions
Note 3 (p. 16): Analysis of features of active parietal endoderm CREs
Note 4 (p. 17): Additional applications of scQers
Note 5 (p. 18): Estimating scale and cost of a scQer experiment
Note 6 (p. 21): Additional Methods details (with table of content)

Supplementary Data Files

Supp. Data 1: sequences of exogenous promoters (**Fig. 2a**) used for benchmarking and as internal standards as well as tested accessible chromatin regions derived from mEB scATAC data (**Fig. 3a**).
Supp. Data 2: high confidence clonotypes and clonotype-assigned cells, human cell lines (**Fig. 2g**, **Ext. Data Fig. 3**).
Supp. Data 3: quantification of variability in activity of integrated reporters [+/-U6/oBC]×[+/-cHS4] (**Supp. Fig. 1**).
Supp. Data 4: positions of SCR elements (**Ext. Data Fig. 6**) and other literature-selected CREs (**Supp. Fig. 3h-i**).
Supp. Data 5: quantification of activity and specificity for all CREs measured with scQers (**Fig. 4a**).
Supp. Data 6: high confidence clonotypes and clonotype-assigned cells, mEBs (**Supp. Fig. 6**).
Supp. Data 7: quantification of activity for all CREs from bulk MPRA time series (**Ext. Data Fig. 9**).
Supp. Data 8: information on CREs with perturbed putative TF binding sites (**Ext. Data Fig. 10**, **Supp. Fig. 2a** & **7d**).
Supp. Data 9: scQer measured activity for paired, mutated, and literature-selected CREs (**Ext. Data Fig. 10**, **Supp. Fig. 2**).
Supp. Data 10: bulk MPRA quantification in mEBs testing different reporter architectures (**Supp. Fig. 8**).
Supp. Data 11: oligos and plasmids used in this work.

SUPPLEMENTARY FIGURES

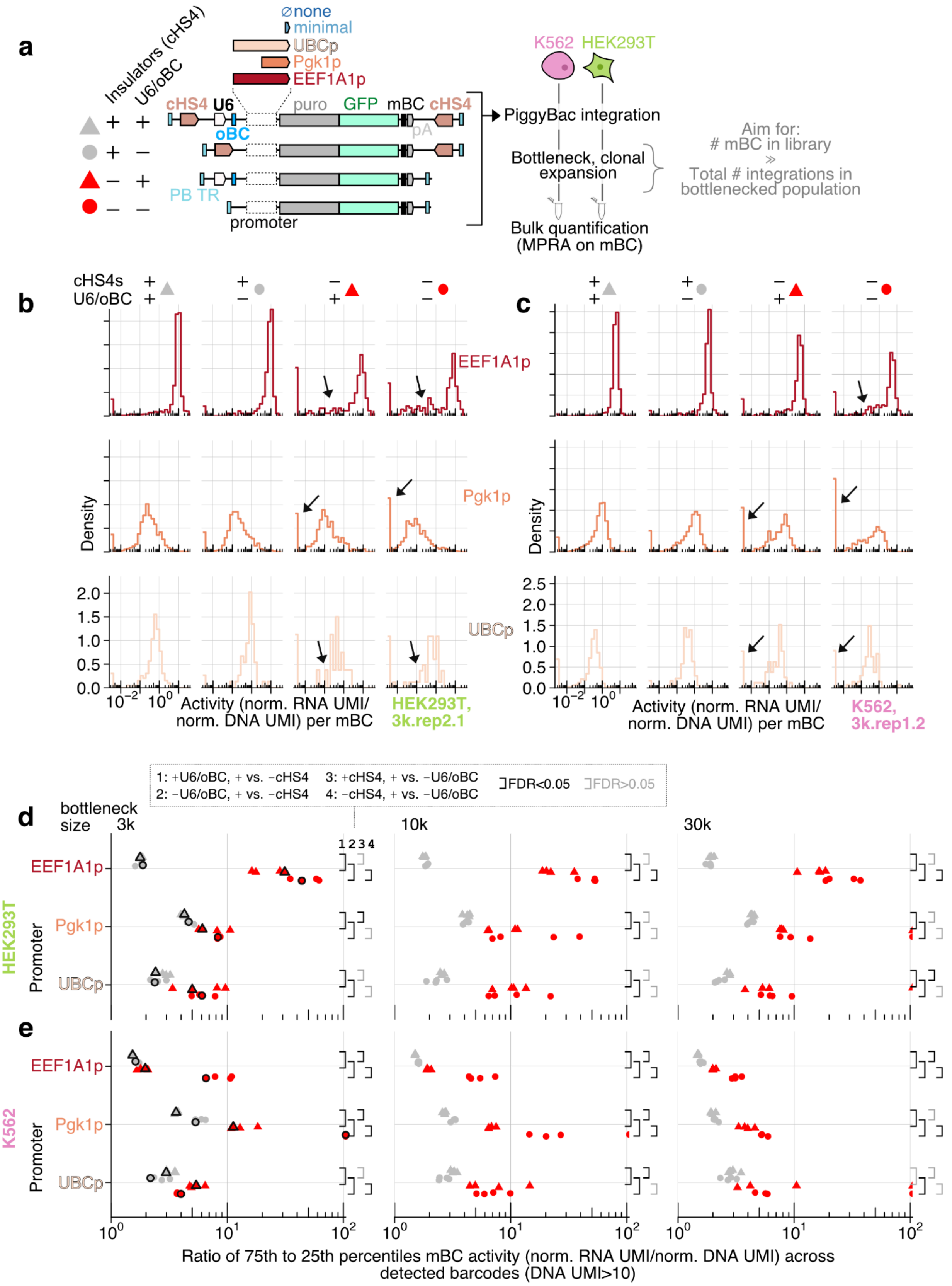

Supplementary Figure 1. Comparison of cHS4 and Pol III U6/oBC cassette for insulating effects. (legend on next page)

Supplementary Figure 1. Comparison of cHS4 and Pol III U6/oBC cassette for insulating effects.

a Schematic of experiment to assess importance of cHS4 and U6/oBC cassettes in mitigating positional effects on randomly integrated promoters. 20 libraries consisting in the 5 ectopic promoters (no promoters, minP, UBCp, Pgk1p, EEF1A1p) in the 4 contexts (+cHS4+U6/oBC, +cHS4-U6/oBC, -cHS4+U6/oBC, -cHS4-U6/oBC), with each library barcoded and pooled prior to integration (piggyBac) in K562 and HEK293T cells, consisting a final pool of 134.1k barcodes uniquely mapped to promoter & reporter architectures. Cells were then bottlenecked (3k, 10k, and 30k estimated starting populations, each in biological duplicates), and MPRA performed on expanded clonal populations (libraries in technical duplicates). In the regime where the number of integrated barcodes is small compared to the total barcode complexity, most barcodes correspond to unique integration events at a specific genomic position. Variability in the per barcode activity across the different reporter architectures provides a measure of the effect of positional variability.

b & **c** Example of mBC expression (norm. RNA UMI over norm. DNA UMI count) distributions for active promoters (rows, top: EEF1A1p, middle: Pgk1p, bottom: UBMp) across different reporter architectures (from left to right: +cHS4+U6/oBC, +cHS4-U6/oBC, -cHS4+U6/oBC, -cHS4-U6/oBC). Arrows highlight wider distributions in per mBC expression in promoters (without insulators), reflecting more positional variability. **b** Replicate HEK293T 3k.2.1, **c** Replicate K562 3k.1.2.

d & **e** Global quantification of mBC expression spread (ratio of 75th to 25th percentiles) across different biological replicates split by promoters and reporter architectures (**c** HEK293T, **d** K562; grey triangles +cHS4+U6/oBC, grey circles +cHS4-U6/oBC, red triangles -cHS4+U6/oBC, red circles -cHS4-U6/oBC). Symbols with black outlines correspond to distributions shown in panels in **b** and **c**. For each cell line, bottlenecked population, and promoters, four comparisons were performed (1: +U6/oBC, + vs. -cHS4; 2: -U6/oBC, + vs. -cHS4; 3: +cHS4, + vs. -U6/oBC; 4: -cHS4, + vs. -U6/oBC), with results of statistical test indicated by colour of square brackets (B-H corrected two-sided Wilcoxon test, grey: $p \geq 0.05$, black: $p < 0.05$). Reporters without cHS4 insulates (red) display substantially more variability in nearly all contexts (35/36), and the U6/oBC also reduces variability though only in some promoter/cell line contexts (H293T: 4/18, K562: 11/18)**.**

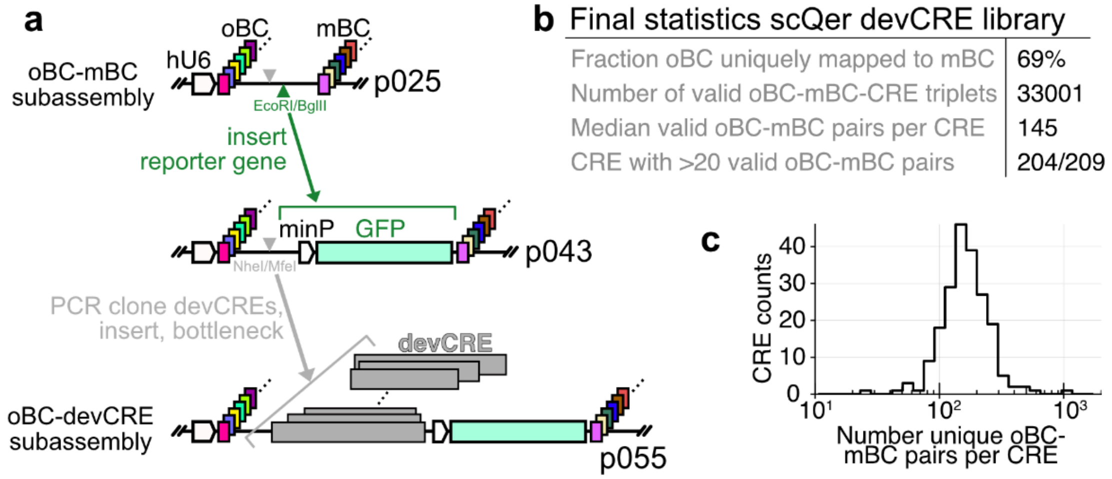

Supplementary Figure 2. scQer library construction and oBC-CRE-mBC subassemblies

a Schematic of procedure to construct doubly barcoded dual RNA reporters. First, a high-complexity (~1 M) library of doubly barcoded (oBC and mBC, separated by multiple cloning site dock) piggyBac transposons is constructed. At this step, oBC and mBC matches are determined (PCR-based library construction). The minimal promoter with GFP cassette is then inserted, and complexity maintained as much as possible. >200 CREs were PCR-cloned, pooled at 1:1 ratios by mass, and inserted in the doubly barcoded minP-GFP backbone by isothermal assembly. The resulting library was bottlenecked to ~50k clones. CRE and oBC matches were then determined on the bottlenecked library (tagmentation with semi-specific PCR). In combination with the initial oBC-mBC pairs, this completes the determination of oBC-CRE-mBC triplets needed to deconvolute single-cell data for reporter activity. Plasmid names (p025, p043, p055) are indicated.

b Compilation of statistics from scQers library used to screen putative CREs in mEBs.

c Distribution of number of unique oBC-mBC pairs per CRE following the subassembly and quality filters, displaying largely uniform representation of the >200 putative regulatory elements tested (experiment **Fig. 3b**).

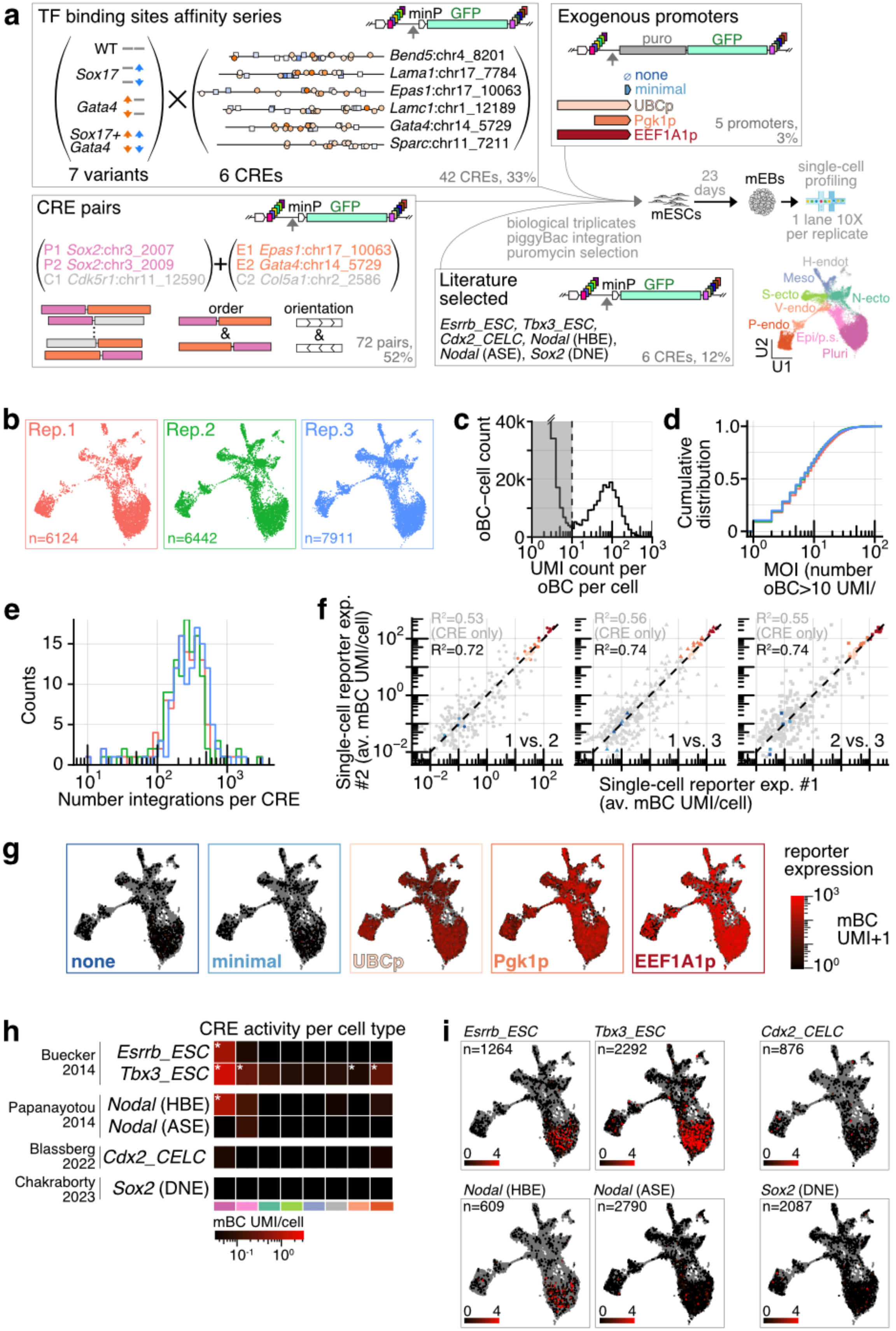

Supplementary Figure 3. Quality control metrics for applications of scQer experiment (legend next page)

Supplementary Figure 3. Quality control metrics for applications of scQer experiment

a Schematic of scQer experiment testing different applications (pairs of CREs, allelic series disrupting/optimising putative TF binding sites, additional CREs selected from the literature). Each library (boxed) was cloned, assembled to barcodes separately, pooled (in proportions shown: 3% ectopic promoter puro-GFP as internal control and to select for high MOI cells, 33% TF binding site allelic series, 52% CRE pairs, 12% literature selected CREs), integrated in mESC, differentiated to mEBs and single-cell profiled (day 23) as described before. Integration to profiling was performed in biological triplicate. Inset shows UMAP of cells passing quality control coloured by assigned cell types.

b UMAP of cells passing quality control files split by biological replicates.

c Distribution of oBC UMI counts per cell per barcode, showing highly bimodal nature, enabling identification of which reporter was present in which cells (>10 oBC UMI/cell, dashed line).

d Cumulative distribution of number of detected reporters per cell (median MOI=7).

e Number of detection events per CRE (or pairs of CRE) across different replicates.

f Expression per CRE per cell type (mean norm. mBC UMI/cell; grey: CREs, coloured: exogenous promoters) for CREs with >0 activity stratified by biological replicate. Each point corresponds to a CRE in a cell-type. R^2 on log-transformed values, including exogenous promoters (black) or not (grey), are indicated (0.72 to 0.74 including ectopic promoters, 0.53 to 0.56 considering only CREs). Lower reproducibility compared to experiment from **Ext. Data Fig. 5b** likely due to lower representations per CRE. Replicates were pooled for downstream quantification.

g Single-cell reporter expression (average normalised mBC UMI per cell) for the five exogenous promoters used as internal controls. Colour scale is logarithmic (with a pseudocount of 1).

h Heatmap quantification of expression per cell type for literature-selected CREs[64–67] (rows: CREs, columns: cell types, following colour scheme in panel **a** inset; colour-bar at bottom). Significant expression over negative controls (noP, minP; B-H corrected bootstrap resampling p<0.01) are indicated by *. The three CREs with significant activity (the *Esrrb* and *Tbx4* CREs from Buecker et al 2014, and the Nodal HBE from Papanayotou et al 2014) were most strongly expressed in pluripotent cells, as expected based on their original reported activity. Two of the 3 elements found to be inactive (a *Sox2* neural CRE, and a *Cdx2* CRE expressed in caudal epiblast-like cells) were likely not expressed as a result of low representation of cognate cell types in our system. Finally, the *Nodal* ASE was lowly expressed specifically in the expected cell type (epiblast cells), but fell below our stringent significance threshold.

i Single-cell map of activity for literature selected CRE (same as panel **h**). Number of detection per CRE indicated. Reporter expression shown from low (black) to high (red) (grey: no detection of CRE of interest).

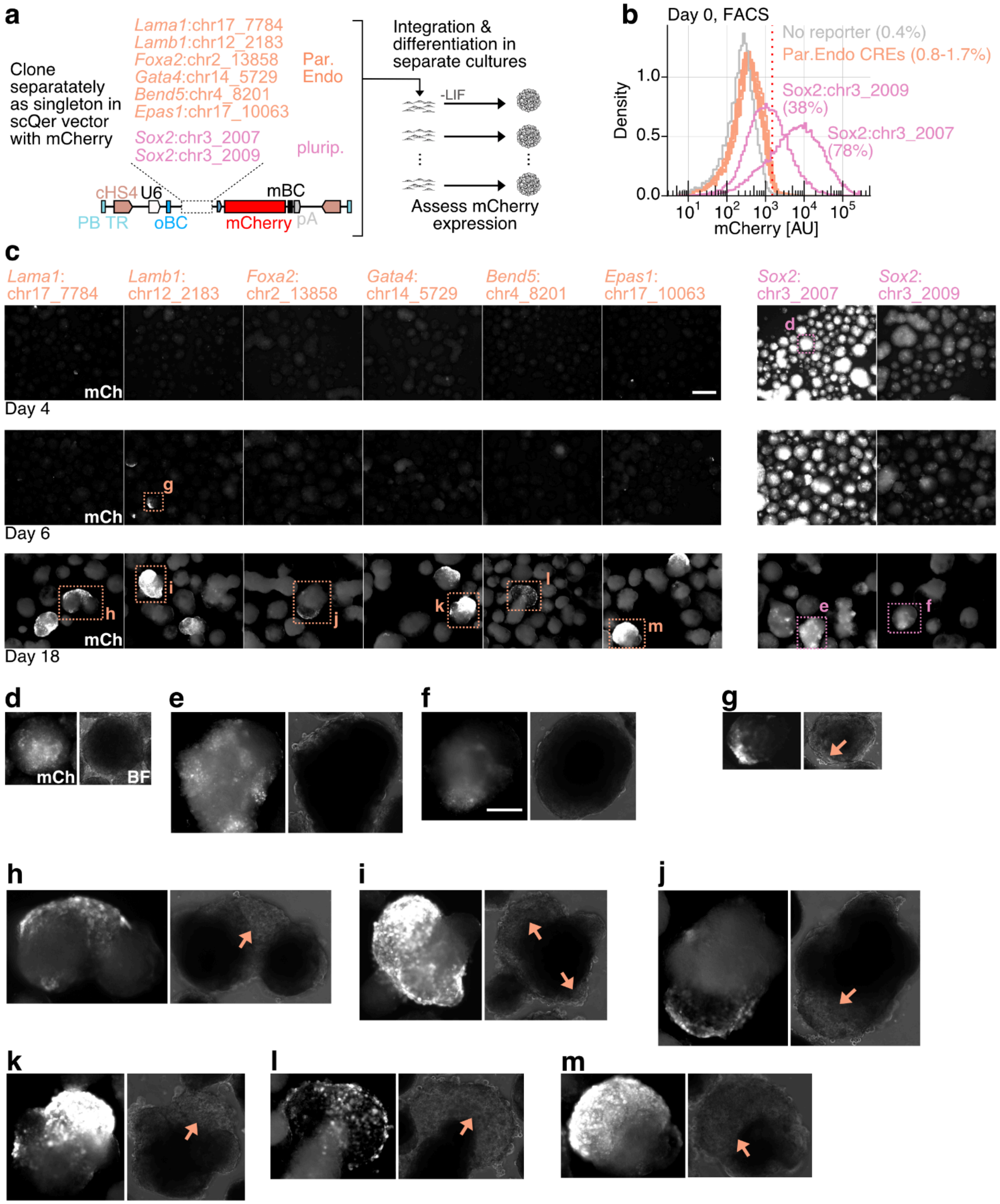

Supplementary Figure 4. Singleton validation experiment of cell type-specific CREs

a Schematic of singleton validation experiments. The 8 most highly cell type-specific CREs as assessed by our single-cell data (**Fig. 4**) were cloned individually in a scQer backbone with mCherry as the reporter gene. Each construct was transfected separately (co-transfection with 10% promoter puro-GFP series), integrated via piggyBac in mESCs and differentiated in mEBs. Epifluorescence images were acquired on alternate days, and

with structured illumination on day 24 (**Supp. Fig. 5**) to assess spatial patterns in expression. One biological replicate per singleton construct was performed (n=8 separate cultures and differentiated samples).

b Distribution of mCherry intensity per cell (FACS data) from single-cell suspension (day of embryoid body induction) of scQer-containing singleton lines. Cells harbouring parietal endoderm-specific elements exhibited limited reporter expression (0.8 to 1.7% above background, defined as the top 0.4% of the no-reporter negative control shown in grey). In contrast, pluripotent-specific elements displayed robust mCherry expression in a substantial proportion of cells (mCherry+: 38% for *Sox2*:chr3_2009, 78% for *Sox2*:chr3_2007).

c Representative fields of view (mCherry channel, Chroma 594 filter Zeiss Axio observer) from individual examples of embryoid body differentiation (columns: different CREs, rows: different times; for each singleton construct and differentiated sample, many EBs showed characteristic expression patterns displayed). Scale bar: 0.5 mm (same scale for all images). All images on the same day have the same exposure (exposure, day 0 & 4: 5 s, day 18: 2 s) and contrast to allow direct comparison. Over differentiation time courses, mCherry signal emerged in a subset of EBs for all parietal endoderm CREs (left six columns). In contrast, the signal decreased in intensity for pluripotent-specific elements (right two columns). Examples of embryoid bodies with localised expression are indicated and zoomed in panels **d**-**m**.

d-**m** Zoomed-in regions from panels in **c**, highlighting spatial pattern of expression of the reporters (for each panel left: mCherry, right: brightfield). Contrast adjusted differently in each panel. For parietal endoderm-specific elements, expression coincided with cells on the surface with rough morphology (arrows), in accordance with described endodermal cells in embryoid bodies[49]. In contrast, *Sox2* control elements displayed internal expression from largely smooth embryoid bodies. Scale bar: 200 um.

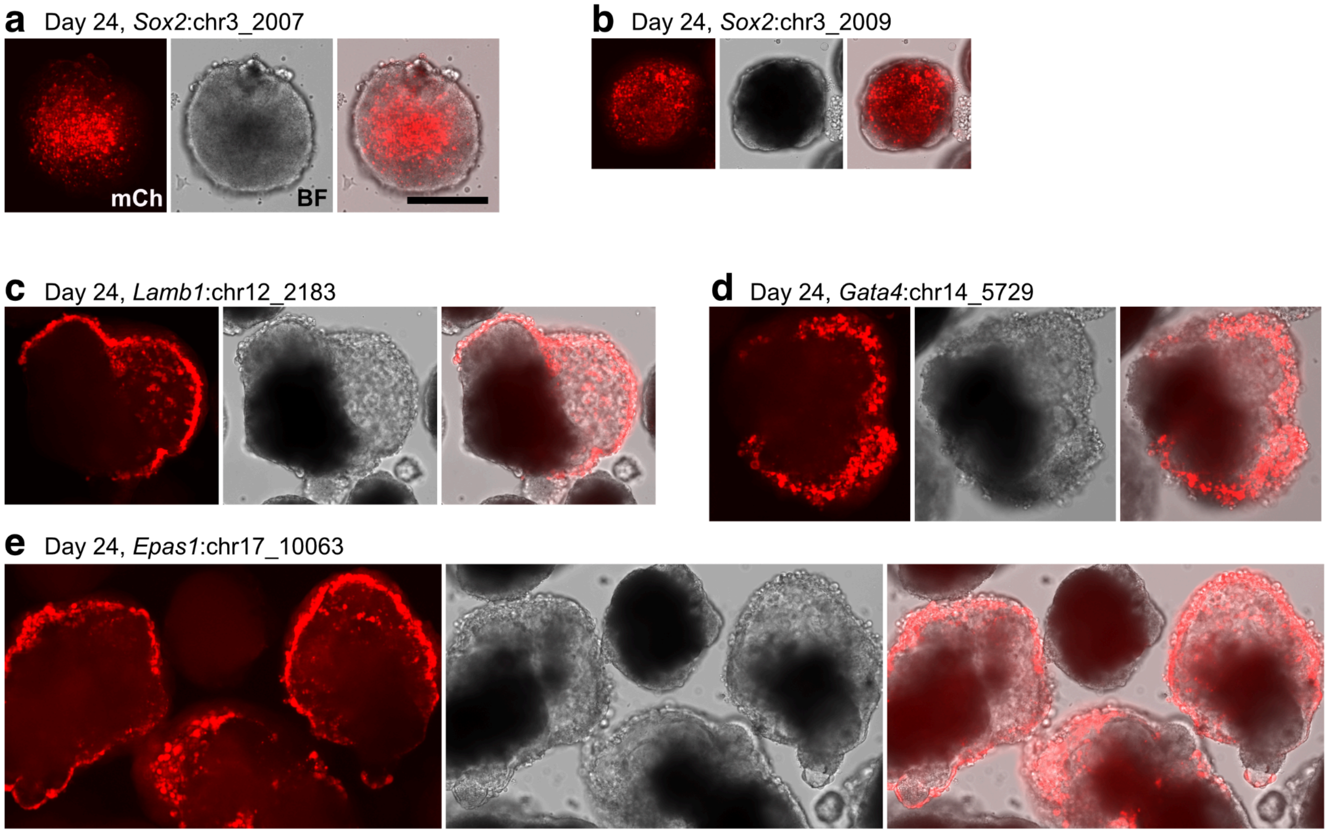

Supplementary Figure 5. Structured illumination images of mEBs with singleton scQer reporters

a-**e** Images (Keyence BZ-X810) from day 24 singleton mEBs harbouring different CREs (same differentiation experiment as **Supp. Fig. 4**, one biological replicate per singleton construct was performed). Left: mCherry (optical sectioning mode: structured illumination, custom pinhole, slit size 2, slit pitch 6, filter cube: chroma Cy3/R 49004), middle: brightfield, right: merge. Pluripotent elements (**a**, **b**) show distinct internal expression compared to parietal endoderm elements (**c**-**e**) which display signal exclusively in surface/rough cells. Scale bar: 250 um (same scale on all images). Images were acquired with the automatic exposure times on mCherry channel (**a**: 0.33 s, **b**: 1.5 s, **c**: 0.33 s, **d**: 0.33 s, **e**: 0.2 s), brightfield: 4 ms.

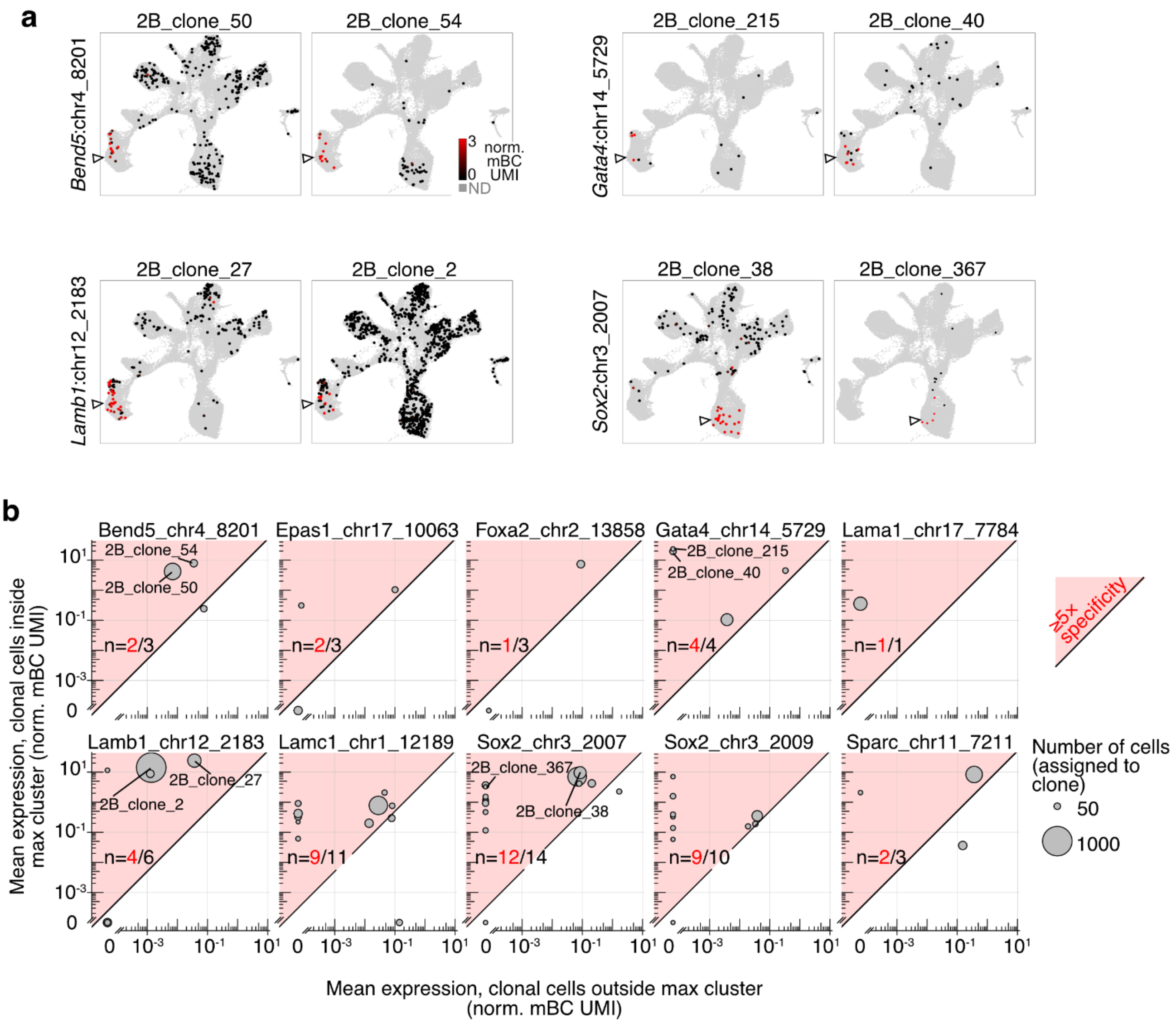

Supplementary Figure 6. Cell-type-specific CRE expression across clones to assess positional integration effects

a Examples of single-cell map of CRE activity for cells assigned to high-confidence clones for four CREs (two representative clones per element shown, marked in panel **b**). Carets indicate the cluster in which expression is expected based on quantification over all cells. Grey points in the background are all other cells not assigned to the clone.

b Systematic quantification of specificity (activity in expected maximum-expression cluster vs. rest of cells, **Fig. 4a**) across all well-represented clones (at least 5 cells in expected maximum expression cluster(s) and at least 5 cells in other clusters) for the 10 CREs identified as active and specific. Each clone is represented by a circle, whose area corresponds to the number of cells assigned to it. Clones shown in panel **a** are indicated. Red shading delineates the region where specificity is in excess of 5-fold. Fractions of clones meeting this criterion for distinct CRE are indicated on each panel. 9/10 CREs have ≥⅔ of their clones with >5-fold specificity.

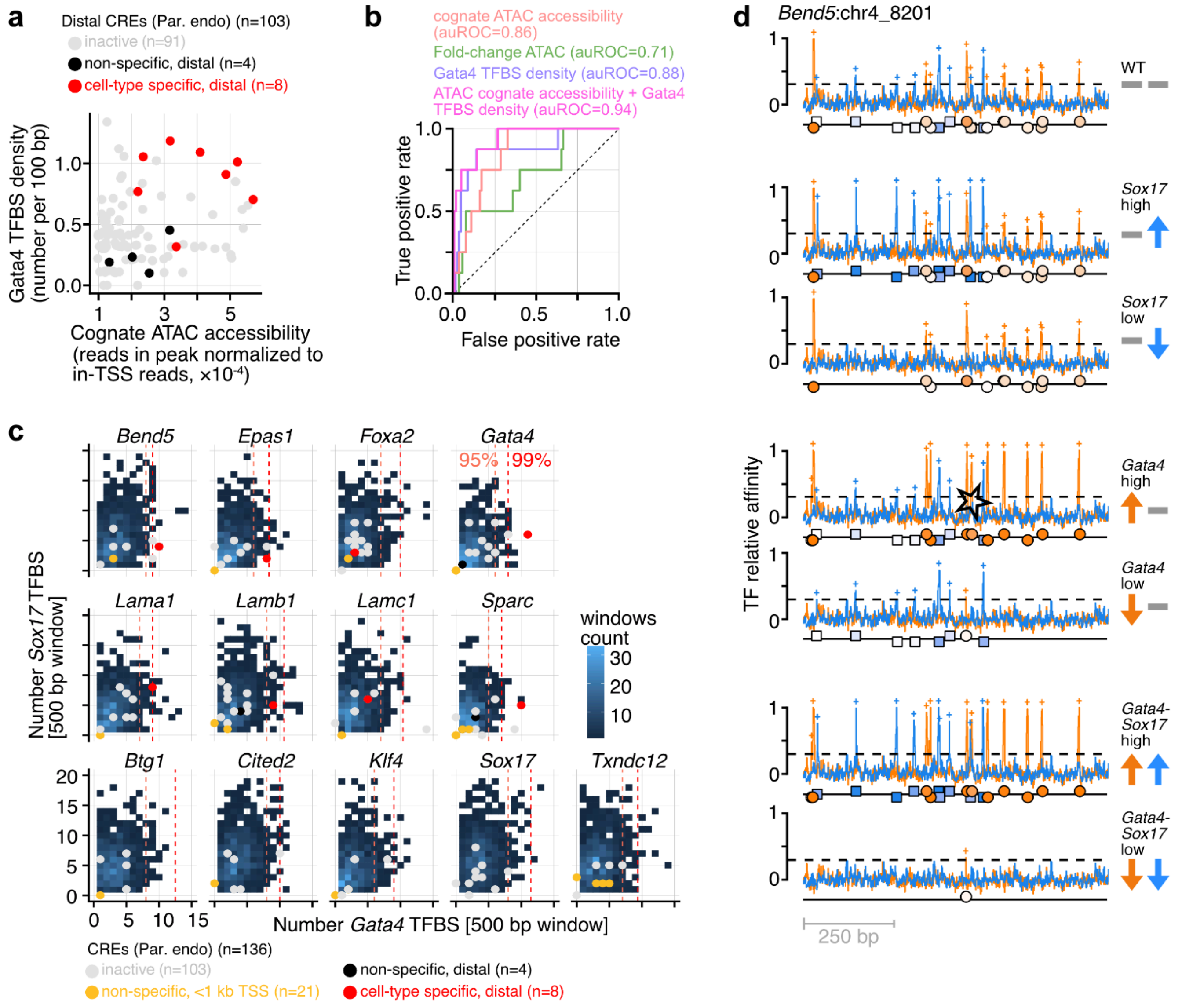

Supplementary Figure 7. CRE features correlated to cell-type-specific activity

a Plot of two features highly enriched for autonomous cell-type-specific CREs: cognate (cell-type corresponding to differential expression of putatively associated gene) ATAC accessibility (x-axis): average in peak reads normalised by reads in TSS (in each cell). y-axis: Density of Gata4 transcription factor binding sites per 100 bp (TFBS with affinity relative to the maximum affinity 8-mer >0.4). Red points mark cell-type-specific CREs. Distal (>1 kb TSS) CREs selected from parietal endoderm loci are shown (n=103).

b Receiver operating characteristic (ROC) curves for the classification task (specific vs. non-specific/inactive) from different features. Density of Gata4 TFBS, cognate ATAC accessibility, and fold-change in ATAC signal have good predictive value to discriminate functional elements (auROC >0.7). A logistic regression classifier including only cognate ATAC accessibility and Gata4 TFBS improves performance to auROC=0.94 (precision=0.6 at recall=0.75). Categories are unbalanced (active=8, inactive=95).

c Sequence analysis of all 500 bp windows (sliding step 250 bp, excluding any window overlapping with CREs with buffer flank position 500 bp on either sides) for the 13 endoderm-specific developmental loci (±100 kb from TSS of indicated gene). For each genomic sequence window, the number of transcription factor binding sites to *Gata4* and *Sox17* (affinity relative to the maximum affinity 8-mer >0.3) is recorded. Panels show the two-dimensional distribution of binding sites numbers across all windows, stratified by loci (parietal endoderm

elements). The number of binding sites is also determined for tested CREs (coloured points; red: cell-type specific, orange: non-specific, <1 kb from TSS, black: non-specific, distal ≥1 kb TSS; grey: inactive) and overlaid on the distributions for comparisons. Cell-type-specific CREs (red points) have an elevated number of Gata4 binding sites compared to other inactive CREs as well as neighboring regions in the locus. Dashed lines mark the 95th and 99th percentile in Gata4 binding site numbers at each locus. 6/8 autonomously active CREs in local top 5%, 4/8 in local top 1% of number of Gata4 binding sites.

d Example of bioinformatic approach to identify putative TF binding sites (shown for CRE *Bend5*:ch4_8201). DNA sequences were broken up in overlapping 8-mer (stride length = 1), and the relative affinity of each 8-mer for *Gata4* and *Sox17* obtained from processed UniProbe data. The trace of 8-mer relative affinity to the different TFs is plotted (*Gata4*: orange, *Sox17*: blue). Putative binding sites are identified as local maxima with relative affinity > 0.3 (dashed line). If multiple local maxima within 3 bp are identified, the maximum affinity position is retained as the putative binding site. Putative sites are marked by crosses (+) above the affinity traces, and are marked below the traces as schematic circles (*Gata4*) and squares (*Sox17*), coloured by the site affinity. Orientation of the putative binding sites is determined relative to a short core PWM (*Gata4*: GATAA, *Sox17*: ACAAT; symbol above line: forward strand, symbol below: reverse strand, symbol on line: no orientation preference). Optimising (disruption) variants are generated by replacing these putative sites by the highest (lowest) affinity 8-mer that is a Hamming distance of 2 away from the putative sites. Affinity traces for the resulting six variants of *Bend5*:ch4_8201 generated and tested experimentally are shown (*Sox17*-high, *Sox17*-low, *Gata4*-high, *Gata4*-low, *Gata4*-*Sox17*-high, *Gata4*-*Sox17*-low). The black star in the *Gata4*-high trace highlights a putative low affinity *Sox17* site disrupted by optimization of the nearby putative *Gata4* site, illustrating possible impacts on other factors' binding of our procedure.

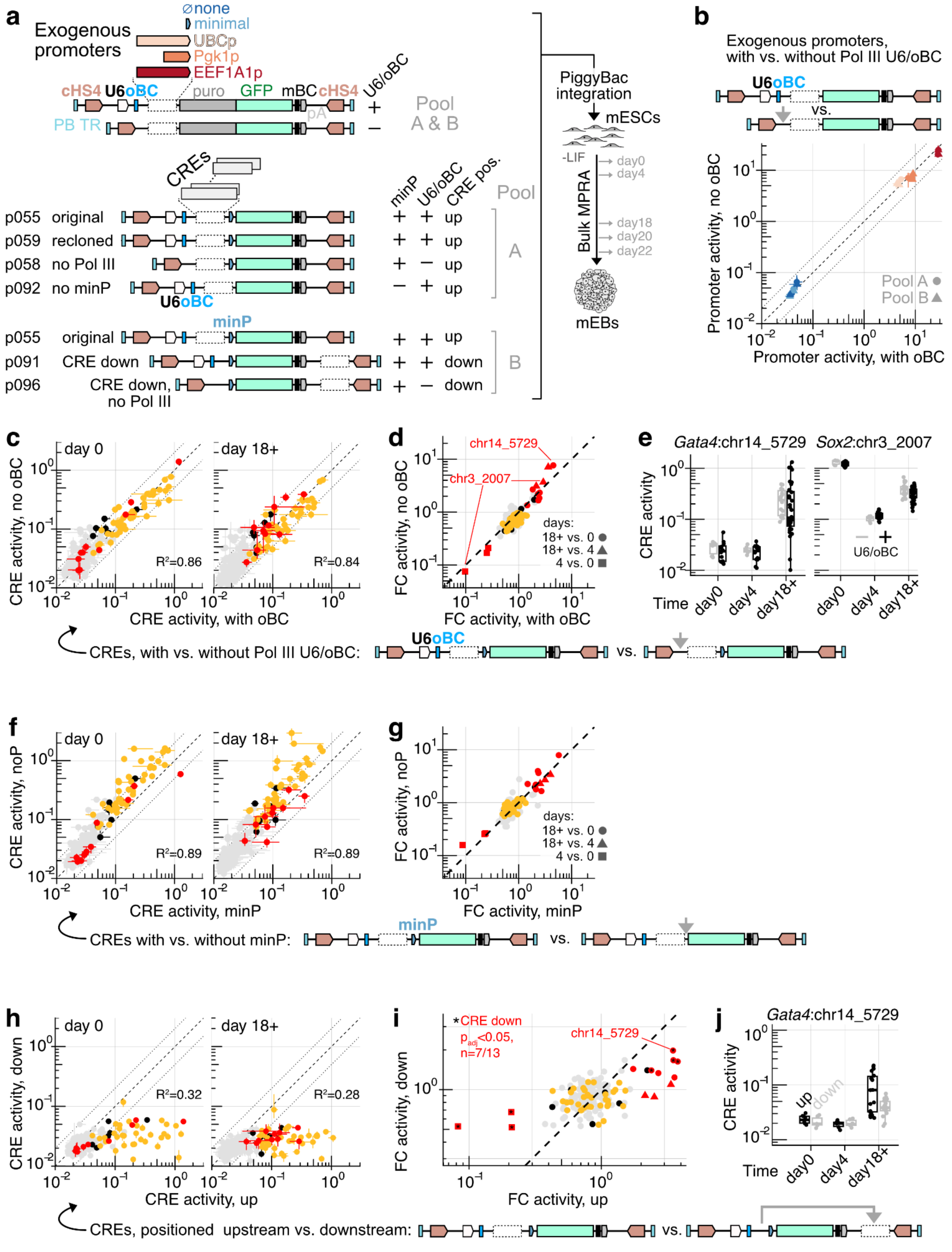

Supplementary Figure 8. Assessing the impact of different reporter architectures (legend on next page)

Supplementary Figure 8. Assessing the impact of different reporter architectures

a Schematics of constructs included in bulk MPRA assessment of the impact of components of the scQer cassette towards measured expression. Each depicted library was cloned and assembled to barcode dictionaries separately prior to pooling (two pools: A & B) for piggyBac integration in mESCs, differentiation to mEBs and bulk MPRA (three biological replicates, MPRA libraries for each replicate and time point prepared in technical duplicates). Both pools included exogenous promoters with and without U6/oBC (top) together with the same CRE library as **Fig. 3** inserted in reporters with different architectures. Pool A included constructs with and without U6/oBC, and without minP. Pool B included constructs with CREs positioned downstream of the reporter.

b Exogenous promoter expression (bulk MPRA activity quantification: sum 1% winsorised normalised RNA UMI over sum 1% winsorised normalised DNA UMI from barcodes associated with promoters over barcodes with >10 DNA UMI) quantified from pool A and B experiments for the with (x-axis) vs. without (y-axis) U6/oBC, highlighting the overall limited influence of the Pol III cassette on the promoter activity. Errorbars correspond to interquartile range over replicates (three biological replicates with two technical replicates each) for a given promoter/time point. Early (days 0, 4) and late time points (days 18, 20, 22) were respectively aggregated for quantification.

c Similar to **b**, but for CREs (left: day 0, right: days 18+), coloured according to the activity characterization categories of **Fig. 4** (grey: inactive, black: non-specific, distal; orange: non-specific, <1 kb TSS; red: cell type-specific). Day 18+ panel aggregates data from days 18, 20, and 22.

d Temporal fold-change in activity of CREs (circles: day 0 vs. day 18+ [all elements except non-monotonic], squares: day 0 vs. 4 & triangle: day 4 vs. day 18+ [elements with non-monotonic temporal dynamics, i.e., Sox2:chr3_2007, Sox2:chr3_2009, and Lamc1:chr1_12189 have intermediate fold-change displayed]) within reporter cassette with (x-axis) vs. without (y-axis) U6/oBC, highlighting the general lack of influence of Pol III transcription towards measured reporter activity. Fold-changes of elements shown in panel **e** are indicated.

e Boxplot (with individual replicate activity shown as beeswarm, whiskers extend to 1.5 times interquartile range, centre median, box extends from 25th to 75th percentiles) of activity of two dynamic regulatory elements (left: *Gata4*:chr14_5729, right: *Sox2*:chr3_2007) at different times, grey: without U6/oBC, black: with U6/oBC. Number of data points: three biological replicates each with two technical replicates per time point (day 18+ quantification includes days 18, 20, and 22). Samples with an oBC cassette (black) also have two separate quantifications originating from cloned libraries p55 and p59 pooled in the experiment (see panel **a**). n=6, 6, 18 (grey, days 0, 4, and 18+ respectively) and n=12, 12, 36 (black, days 0, 4, 18+ respectively).

f & **g** Same as **c** & **d**, with comparison with vs. without minP, demonstrating overall lack of importance of the minimal promoter for the measured activity.

h & **i** Same as **c** & **d,** with comparison of CREs positioned upstream (original) vs. downstream of the reporter, showing generally muted expression. However, temporal fold-change is still significant for 7/13 dynamic CRE/time point comparisons (Bonferroni adjusted one-sided Wilcoxon test p-value for cell type-specific CRE comparisons, CRE/time comparisons with p<0.05 are marked by *).

j Same as **e**, demonstrating that element *Gata4*:chr14_5729 still leads to activation of reporter expression even when positioned downstream of the reporter. Number of data points: three biological replicates each with two technical replicates per time point (day 18+ quantification includes days 18, 20, and 22). n=6, 6, 18 (days 0, 4, and 18+ respectively).

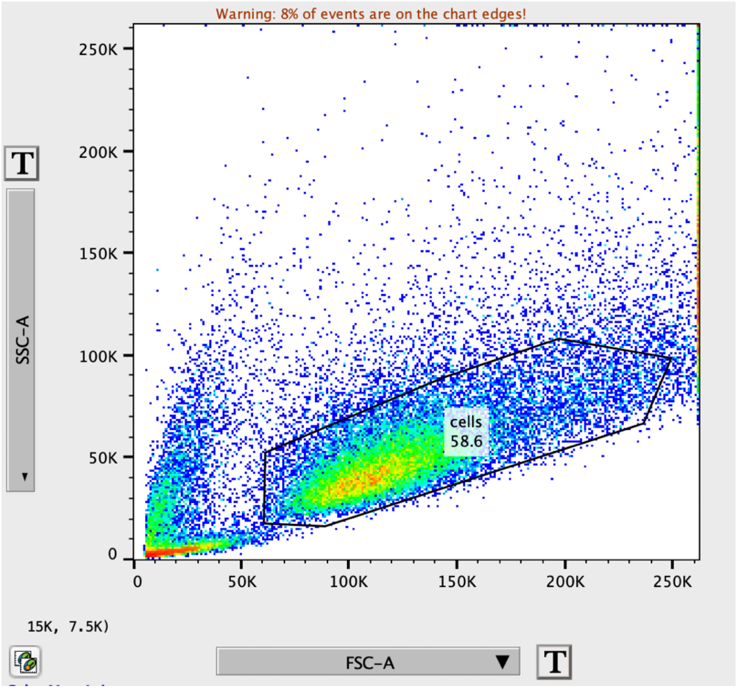

Supplementary Figure 9. FACS gating strategy

Example of a typical FACS gating strategy used to obtain high quality single-cell suspension from dissociated cells prior to 10x Genomics library preparation.

SUPPLEMENTARY NOTES

Supplementary Note 1: Systematic assessment of integration positional effects

Clonal analysis (**Fig. 2d**) was also informative with respect to reporter expression variation driven by positional effects (assuming distinct clones harbour reporters integrated at different genomic locations). We observed promoter and cell line-specific effects, with EEF1A1p and UBCp showing remarkably little clone-to-clone variation (interquartile range across clones, UBCp: <2.4 for all cell lines; EEF1A1p: <1.5 in K562 and HEK293T, 4.1 in HepG2, **Ext. Data Fig. 3j**). In contrast, promoter Pgk1p showed both cell line differences in expression and higher variability across clones (IQR 4.8 in HEK293T, 5.9 in K562, 7.2 in HepG2, **Ext. Data Fig. 3j**). Decomposing the mBC UMI variability into positional effects (via clone assignment) vs. the sum of remaining biological and technical noise, showed that precision was limited by genomic context, underscoring the low variability of our capture and the importance of averaging over multiple independent integration positions (fraction mBC UMI variance attributable to clone identity: EEF1Ap=0.60, Pgk1p=0.41, and UBCp=0.57). Still, for the three active promoters considered here, clone-to-clone variability was substantially lower than that of uninsulated reporters[45], suggesting that insulators included in our design (**Ext. Data Fig. 1a**) partially mitigated positional variegation. Integration of promoter-driven reporters with four possible architectures ([+/-U6/oBC]×[+/- flanking cHS4]) provided additional evidence that the cHS4 insulator[48] indeed reduced positional effects across cell lines and promoters. Interestingly, we also found significant though modest and context-specific insulating effects of the U6 cassette (**Supp. Fig. 1**, **Supp. Data 3**).

Supplementary Note 2: Comparing CRE activity and putatively associated gene inductions

For endoderm-specific CREs, the magnitude of activity induction (fold-change of mean norm. mBC UMI per cell in parietal endoderm vs. pluripotent) was on par with endogenous gene induction (fold change parietal endoderm to pluripotent, **Ext. Data Fig. 8f**).

What proportion of endogenous regulation do the identified autonomous CREs recapitulate? This question is difficult to directly address because absolute reporter UMI counts cannot be uniformly compared to gene expression UMI counts (i.e. due to gene-to-gene differences in conversion between endogenous mRNA levels and captured UMI counts). Taking activity of the active promoter putatively associated with the induced gene (orange in **Fig. 4e**, **Ext. Data Fig. 8d**) as a baseline (with the caveat that mRNA levels driven by promoters in our reporter system might not be perfectly reflective of endogenous activity), we found that the activity of the autonomous CREs captured a substantial proportion of the expression fold-change, but in 6/7 cases less than a half (shaded **Ext. Data Fig. 8g**), as perhaps expected for multi-CRE landscapes as considered here.

Supplementary Note 3: Analysis of features of active parietal endoderm CREs

A number of features were enriched in the 8 active cell-type specific CREs within all 103 tested distal parietal endoderm elements tested. Active CREs displayed higher chromatin accessibility (1.8-fold more accessible, 2.2-fold more differentially accessible, both p<0.03 B-H corrected one-sided t-test), but showed no difference in evolutionary conservation (average phyloP score[108]), nor were they significantly closer to the TSS of their putative target gene. Indeed, at all loci, the autonomously active CRE was not the closest element from the TSS (**Fig. 4b**, **Ext. Data Fig. 8b**). Active elements also showed no evidence of opening earlier than other elements in a pseudotime analysis[109], arguing against them being 'seed enhancers'[110,111]. With regards to finer-level sequence features, active CREs contained a higher density of endodermal regulator Gata4 binding sites, but only if considering binding

sites of intermediate-to-high affinities (between 1.3 and 2.2-fold more binding sites for relative affinity lower thresholds between 0.2 and 0.45, p<0.03 B-H corrected one-sided t-test, 8-mer affinities from Uniprobe[104–106], binding sites also elevated comparing to all 500 bp windows ±100 kb from TSSs, **Supp. Fig. 7c**). While additional examples are needed to draw general conclusions, this suggests clusters of intermediate affinity binding sites of key regulators might be important for mammalian developmental CRE function, in line with the suboptimization hypothesis[27,112]. Two other endodermal regulators, Foxa2 and Sox17, did not show a higher number of binding sites in active CREs. In short, active parietal endoderm CREs displayed significantly elevated ATAC accessibility and Gata4 transcription factor binding sites (**Supp. Fig. 7a**), with a logistic classifier using these two properties accurately classifying active/inactive elements (auROC=0.94, **Supp. Fig. 7b**, precision=0.6 at recall=0.75).

Supplementary Note 4: Additional applications of scQers

Pleiotropic expression from synthetic pairs of CREs

The modularity of CREs, i.e., their ability to function independently and collectively direct complex activity profiles, is a cornerstone of regulatory genomics[4]. To generate pleiotropic expression patterns[113,114] in our *in vitro* system, we created a library of chimeric CRE pairs (pluripotent-specific paired with parietal endoderm-specific and inactive elements as controls, **Ext. Data Fig. 10a**). The pairs were assembled in all possible orders and orientations (72 different possibilities, combinatorial library construction using Gibson assembly with shared homologies, association of CREs to barcodes using nanopore sequencing). The resulting scQer library was profiled as before in mEBs (QC metrics, **Supp. Fig. 2**, **Supp. Data 9**).

Cell-type-specific CREs displayed expression in their expected cell types when paired with inactive elements (B-H corrected bootstrap p<0.01), **Ext. Data Fig. 10b-c**. Importantly, the majority of active pairs led to significant expression in both cognate cell types (7/8, B-H corrected bootstrap p<0.01), **Ext. Data Fig. 10b-c**, consistent with the expectation of additive CRE activity. Notably, CRE order had a substantial effect on quantitative activity, with the promoter proximal element displaying higher expression across all constructed pairs (B-H corrected bootstrap p<0.01; median fold-change expression decrease in proximal vs. distal-containing pairs: *Epas1*:chr17_10063: 21.8, *Gata4*:chr14_5729: 15.8, *Sox2*:chr3_2007: 4.9, *Sox2*:chr3_2009: 7.4), similar to the decrease observed for reporters with CREs placed downstream of the reporter (**Supp. Fig. 8h**). Identifying the molecular origin of this effect is beyond the scope of this work, but these scQer measurements highlight opportunities and challenges towards engineering complex yet quantitative expression profiles in multicellular systems from synthetic CRE assemblies.

Profiling allelic series of CREs with optimised/disrupted transcription factor binding sites

Transcription factor binding to regulatory DNA is the proximally mechanistic step towards induction of gene expression. Changing TF-DNA binding affinity through perturbations to sequence can lead both to loss and gain-of-function (e.g., by optimising binding affinity[27,112,115]).

To assess importance of binding affinity of developmental transcription factors (TFs) within identified cell-type-specific CREs, we focused on *Gata4* (and *Gata6* given similarity of motifs) and *Sox17* (endodermal TFs[116], differentially expressed, motifs with enrichment in accessible chromatin). For six parietal endoderm elements, wild-type sequence and six variant CREs with optimization and disruption of *Gata4*/*6* and *Sox17* putative binding sites were designed (WT, *Sox17*-high, *Sox17*-low, *Gata4*-high,

Gata4-low, *Gata4-Sox17*-high, *Gata4-Sox17*-low). Identification of putative binding sites, together with mutated sequences with high/low affinities, were based on *in vitro* biophysical measurements (UniProbe[104]*Gata4*[105], *Sox17*[106], **Supp. Fig. 7d**, **Supp. Data 8**). The resulting allelic series was cloned as a pool in a scQer library, and its activity profiled in mEBs as before, **Ext. Data Fig. 10d** (same experiment as **Ext. Data Fig. 10a-c**, **Supp. Fig. 2**, **Supp. Data 9**).

Our hypothesis was that disruption and optimization of TF binding sites would respectively ablate and increase activity across the CREs profiled. Data instead revealed a diverse range of effects (**Ext. Data Fig. 10e**). Disruption of *Gata4/6* binding sites universally ablated function (lower than WT for 6/6 *Gata4*-low and 6/6 *Gata4-Sox17*-low CREs, B-H corrected bootstrap p<0.01 compared to WT, median reduction: 77-fold for *Gata4*-low, and 75-fold for *Gata4-Sox17*-low), e.g., **Ext. Data Fig. 10f**. Surprisingly, *Gata4/6* binding site optimization also often decreased activity (3/6 CREs), and never significantly increased it, suggesting either that sequences chosen from biophysical measurements did not reflect actual TF affinity in cells, that the sequence changes also altered binding of other TFs nearby (e.g., *Sox17*, **Supp. Fig. 7d**), or that increased binding inhibited function, as has been reported in other contexts[117]. Decrease of *Sox17* binding sites affinity was more innocuous (significant decrease in expression in 1/6 CREs) while optimization had varied effects (2/6 CRE with increased and 1/6 CRE with decreased activity). Notably, a drastic >10-fold higher expression was observed for *Sparc*:chr11_7211 with optimised *Sox17* sites compared to WT (**Ext. Data Fig. 10g**). This mutated element also displayed an increase in a similar cell type (visceral endoderm). Finally, optimising both *Gata4* and *Sox17* sites tended to decrease expression (3/6 CREs), and never to increase it.

In sum, profiling CREs with systematically perturbed putative binding sites with scQers confirmed the functional importance of these sequence features while underscoring the extent of context-dependence and CRE-specific responses to multi-position changes in sequences.

Supplementary Note 5: Estimating scale and cost of a scQer experiment

<u>Synthesis of CREs</u>

One of the factors limiting the scale of the reported experiment was our desire to test sequences of ~1 kb in size (as a rough lower bound on the sequences tested in vivo compiled in the VISTA database[118] and a compromise for PCR cloning). Indeed, we were unsure whether sequences on the scale synthesizable on arrays (200-300 bp) would be fully functional in a developmental system. Since our CREs were PCR-cloned for cost reasons, we aimed for a 96-well plate scale, i.e., hundreds. Given our success rate (97.6%, 204/209 recovered), we conceivably could have gone for a larger number. We anticipate that as longer DNA synthesis becomes more readily available at scale, or the biology of developmental CREs becomes better characterised (such that working with shorter tiles is unequivocally shown to suffice), this will not be a limiting consideration. We also consider this limitation (cost of synthesis) to be shared with all methods studying regulatory elements and can be circumscribed by other means (e.g., testing fragments of the genome enriched for functional elements, as has extensively been done in previous work, e.g., ATAC-STARR-seq[119], FAIRE-STARR-seq[120]).

<u>Cloning and assembly of barcode dictionary</u>

Assuming a (possibly highly) complex starting library of CREs, our cloning strategy relies on adding the elements in a pre-barcoded backbone. The barcodes in the backbone are generated using random primers, and using standard electroporation and molecular biology, library complexity of millions can routinely be achieved (e.g., our starting backbone included ~1M oBC-mBC pairs). Following CRE addition and bottlenecking, the oBC-CRE-mBC triplet dictionary needs to be generated. Our current approach with

tagmentation & semi-specific PCR could be streamlined in future iterations (especially if the CREs profiled are shorter) to be done in a single step of PCR and mapped with paired-end sequencing. In terms of sequencing cost associated with this procedure, we estimate that 25-fold read coverage of the library complexity (number of unique oBC-CRE-mBC) should suffice to obtain high-confidence triplet assignments. For library complexity, we suggest about 100 oBC-mBC pairs per CRE; a larger barcode space decreases the likelihood of spurious chimeric counts being tallied towards quantification. For our library, with ~200 CRE each with ~200 BC pairs, the sequencing coverage necessary for assignment can be estimated to be $200 \times 200 \times 25$ = 1M read, which is a modest cost (0.25% of NextSeq2000 P2, ≈4$). More generally, the number of necessary reads for dictionary construction is estimated to be (# CRE)×100 BC pairs × 25 reads/triplet = (# CRE) × 2500. Even for libraries of thousands of CREs, these sequencing costs amount to a small fraction of the overall sequencing budget, as detailed below.

Single-cell experiment and sequencing

We now turn to the question of scale for the limiting step: the single-cell experiment. We estimate the per-CRE-characterization-cost as a function of a few simple parameters, some set by the biological system of interest and exact nature of the library of elements.

One key variable is how many observations (detections) per CREs are needed to perform a reliable measurement. Our random transgenesis approach comes at the cost of the need to average over barcodes (i.e., over integration positions). Empirically for the cell type-specific elements we discovered (limit of detection at about average 1 mBC UMI per cell) with droplet-based 10X Genomics assay, we find that ~20-40% of mBC had a detected valid UMI in their cognate cell type. Demanding about 20 non-zero observations for averaging positional effects leads to an estimate of 100 detections per cell type. Hence, the total number of cells that need to be profiled per CRE (per biological replicate) for high sensitivity is on the order of 100 detections/cell type × the number of cell types. So, the total number of cells to be profiled can be estimated at around (assuming similar composition of cell types, more cells would need to be profiled for rare cell types etc.):

$N_{cell} = N_{CRE} \times$ (100 detections/CRE/cell type)×(number of cell types)×(replicates)/MOI.

With parameters in our first experiment (MOI ≈20, ≈10 cell types), this amounts to: $N_{cell} \approx N_{CRE} \times 50 \times$ 3 rep. A simple way to increase the cost efficiency of scQer is to work with CREs prioritised by other functional methods (e.g., bulk characterization), increasing the obtained information from the single-cell experiments.

Sequencing coverage needed:

GEx: We estimate from experience that r_{GEx} ≈4000 reads per cell suffice in many applications (one could conceivably go lower, especially with diverse cell types) to reliably map cell types and obtain good single-cell resolution.

mBC: we typically found those libraries easy to sequence to saturation, in part because a high proportion of the tested CREs were inactive. If working with an heterogeneous sample (multiple cell types) with CREs tested anticipated to have cell type specificity, this should remain largely valid. As a point of reference with our biotype and libraries, r_{mBC}≈40 reads per mBC per cell on average led to about 4-fold saturation (4 reads per mBC UMI detected).

oBC: given the high expression (across all cells) and excellent single-cell capture of the oBC, saturating coverage would be costly (estimated UMI complexity per oBC per cell 0.8-2.5k depending on the biotype, which means with an MOI of 20, ≈20k total complexity; so that 5-fold coverage would require 100k reads per cell). Thankfully, reliable reporter detection does not require the full captured oBC complexity to be sequenced. In experiments described in this work, 300 reads per oBC per cell lead to about 5% duplication

rate (reads/UMI-1) and near deterministic per cell barcode detection. Importantly, not all oBC UMIs need to be captured to reliably perform this binary detection task. We estimate that 4-fold less coverage, or $r_{oBC} \approx 75$ reads per oBC per cell, would suffice.

Adding these contributions, we arrive at an estimate of the total number of reads scaled by the number of cells profiled (with MOI=20):
$R = N_{cell}\ (r_{GEx}\ + MOI \times r_{mBC} + MOI \times r_{oBC}) \approx N_{cell}\ (4000 + 800 + 1500)$

Hence, while the oBC and mBC do not constitute a negligible addition to the estimated sequencing budget, given their lower respective needs compared to GEx, we estimate (ultimately dependent on the exact application) that ~60% more reads are needed to sequence a single-cell reporter experiment (relative to a conventional single-cell GEx experiment).

Total cost estimate (single-cell + sequencing):

The costs need to account both for the single-cell library reagents ($c_{sc/cell}$ per cell cost) and the sequencing (c_{read} per read cost). Together with earlier equations, this amounts to:

$Cost = c_{sc/cell} * N_{cell}\ + c_{read}\ R = N_{cell}\ (c_{sc/cell} + c_{read}\ [r_{GEx}\ + MOI \times r_{mBC} + MOI \times r_{oBC}])$

Together with our estimate for the number of cells per CRE above, this constitutes a framework to estimate the scale of the experiments. We can estimate actual costs. For 10X Genomics reagents, the per cell cost $c_{sc/cell}$ is about 0.15$/cell. The cost with a Nextseq2000 P3 100 cycles kit is at about 2.6$ per M reads (Fall 2023). Hence:

Cost (10X Genomics) =
N_{cell} (0.15\$/cell + 2.6×10^{-6} \$/read [4000 + 800 + 1500 reads/cell]) =
N_{cell} (0.15\$/cell + 0.016\$/cell)

Above, the sequencing and single-cell library preparation costs are kept separate to illustrate the different components, and shows that the bulk of the cost remains on the single-cell library generation with the droplet-based approach. Importantly, we note that alternatives to droplet-based single-cell approaches are constantly being developed and improved upon, most notably single-cell combinatorial indexing (sci)[83]. Based on this existing optimised sci protocol, the per cell cost decreases by about a factor of 100. Hence, we anticipate that throughput gains on the order of a factor of 10 at similar costs will be achievable in the near future.

Supplementary Note 6: Additional Methods Details

1 Benchmarking and optimization: promoter series in human cell lines 22
1.1 Single-cell reporter libraries preparation 22
1.2 Optimization of reporter RNA capture 23
1.3 Estimating per oBC per cell captured library complexity 24
1.4 Estimating the probability to have multiple integration per cell for one plasmid 24
1.5 Clonal cell analysis 25
1.5.1 Clonotype identification 25
1.5.2 Mapping of cells to clonotypes 26
1.5.3 Systematic oBC dropout analysis (precision-recall) 26
1.5.4 Analysis of reporter barcode expression variability across clones 27
2 Profiling developmental cis-regulatory elements in mouse embryoid bodies 28
2.1 Optimization of high multiplicity of integration with piggyBac in mESCs 28
2.2 scATAC-seq on mEBs 28
2.2.1 Experimental method 28
2.2.2 Processing of scATAC-seq data 29
2.3 Prioritization of developmental loci and putative CRE selection 29
2.4 Cloning details 30
2.4.1 Recloning of oBC-mBC backbone plasmid 30
2.4.2 oBC-mBC subassembly 30
2.4.3 Construction of EEF1A1p-mCherry transposon plasmid 31
2.5 Single-cell reporter libraries preparation and sequencing 31
2.5.1 Quality filtering from gene expression libraries 32
2.5.2 mBC and oBC libraries 32
2.5.3 Single-cell quantification of reporter expression 32
2.6 Bulk MPRA (CREs, mEB time series experiment) 33
2.6.1 Library preparation and sequencing 33
2.6.2 Data processing and quantification 33
2.7 Single-cell data integration 34
2.7.1 Integration between scRNA-seq and Pijuan-Sala et al in vivo scRNA-seq 34
2.7.2 Integration between scRNA-seq and scATAC-seq and correlation with in vivo data 35
2.8 Clonal cell analysis 36
2.8.1 Clonotype identification, refinement, cell assignments, basic metrics, and dropout assessment 36
2.8.2 CRE expression pattern across clones 36
2.9 Analysis of features of profiled putative developmental CREs 36
3 Pol III driven circular vs. linear barcode MPRA experiment 37
3.1 Cloning of plasmids 37
3.2 Transfection, cell culture, and cell harvesting 38
3.3 Massively parallel reporter assay library generation and sequencing: 38
3.4 Data pre-processing and quantification 39
3.5 Estimating expression levels of oBC per cell per integrated cassette 40
4 Assessing the influence of cHS4 and U6/oBC on genomic integration positional effects 41
4.1 Experiment description and statistical rationale 41
4.2 Construction of reporter libraries with/without cHS4 and U6/oBC 42
4.2.1 Cloning of reporters 42
4.2.2 Association of mBC to promoters 42
4.3 Transfection and bottlenecking of cell population 43
4.4 Bulk MPRA experiment and data analysis 43
4.4.1 Bulk MPRA library construction 43
4.4.2 Bulk MPRA analysis 44

5 Control experiments assessing impact of reporter architecture (bulk MPRA in mEBs).. 44
5.1 Construction of reporter libraries with different architectures.. 44
5.1.1 Cloning of reporter libraries.. 44
5.1.2 mBC to CRE subassembly strategies.. 46
5.2 Transfection of final pooled libraries and mEB induction.. 47
5.3 Bulk MPRA experiment and analysis... 47
6 Singleton validation experiments... 49
6.1 Cloning of singleton mCherry scQer reporters... 49
6.2 Singleton mEB differentiation experiment.. 49
7 Example applications of scQers: CRE pairs and TF binding sites allelic series.. 51
7.1 Identification of putative transcription factor binding sites.. 51
7.1.1 Putative Transcription factor binding sites identification... 51
7.1.2 TF binding site optimization and disruption.. 51
7.2 Cloning of scQer libraries... 52
7.2.1 Literature selected elements... 52
7.2.2 Paired CREs combinatorial assembly.. 52
7.2.3 CREs with mutated putative transcription factor binding sites.. 53
7.3 Generation of barcode to CRE dictionaries.. 53
7.3.1 Connecting pairs of CREs to barcodes with long read (Nanopore) data... 53
7.3.2 CRE with allelic series of perturbed transcription factor binding sites... 54
7.3.3 Final barcode dictionary for pooled experiment... 55
7.4 scQer experiment, data processing, and analysis... 55

1 Benchmarking and optimization: promoter series in human cell lines

1.1 Single-cell reporter libraries preparation

For single-cell reporters, three libraries are generated: the standard 3' gene expression (GEx) library from 10x, and two custom derived libraries, one for each reporter RNA (oBC and mBC). The latter are obtained from nested PCRs from the amplified cDNA as we detail below.

Briefly, single-cell library preparation proceeded following the manufacturer's protocol (v3.1 manual CG000205 Rev D, 10x Genomics), with some critical modifications listed here. First, one of the replicate's cDNA (replicate B) was split in two equal halves (and brought to same final volume with elution solution 1) after GEM RT cleanup (step 2.1.s) prior to cDNA amplification to allow for a direct comparison the UMIs captured with different enrichment strategy (hereafter replicate B1 and B2). For cDNA amplification, primers specific to the mBC (oSR38) and oBC (oJBL246) reporter transcripts were spiked-in the reaction (similar to TAP-seq[107]) at final concentration of 0.5 uM to boost UMI capture for replicates A and B1 (but not for replicate B2, to allow direct comparison with replicate B1). Following cDNA amplification, both the bead and supernatant derived material (steps 2.3Ax and 2.3Bxiv respectively) were saved for downstream processing.

Gene expression libraries for all replicates were prepared following the manufacturer's protocol from 25% of the bead fraction amplified cDNA.

oBC enriched libraries were prepared as follows. For replicate B2 (no primer spiked in), a first outer PCR1 was performed using 25% of the supernatant amplified cDNA with primers oSR40+oJBL246 using Kapa Robust (Roche) and tracking with qPCR until the inflection point (50 uL 2x master mix, 12.5 uL supernatant cDNA, 5 µL 10 µM oJBL246, 5 µL 10 µM oSR40, 0.5 uL SYBr green, and water to 100 µL; run parameters: 3 min at 95C, and cycles 20 s at 95C, 20 s at 60C, 20 s at 72C).

Amplicons were cleaned up with 1.75x Ampure XP beads, and 1/10 of the eluate was carried to the inner PCR with the remaining replicates. For replicates A and B1, the outer PCR was performed during the cDNA amplification via the spiked-in primer, and 25% of the supernatant amplified cDNA was taken as input for the next PCR. Semi-nested inner PCR was performed on all samples with primers NextP5_index1 and indexed primers oJBL425-oJBL427, with the same parameters as PCR1 and stopped before the inflection point. Final libraries were purified by 1.5x Ampure XP beads.

As a result of our Pol II reporter construct having a capture sequence (CS2, **Ext. Data Fig. 1a**) downstream of the mBC, reporter mRNAs could be captured from both the poly-dT and CS2 reverse transcription primers on the 10x beads. To systematically compare capture efficiency resulting from the two types of primers, two different libraries were generated (poly-dT captured, and CS2 captured). For poly-dT captured libraries, similar to oBC libraries, we first performed outer PCR on replicate B2 (no spiked-in primers in cDNA amplification) using primers oSR38+oJBL207, using the same PCR conditions as for oBC except for an elongation time of 50 s and an anneal temperature of 65C. 25% of the bead fraction of the purified amplified cDNA was used as template. Following 1x Ampure XP clean up, 10% of the eluate was taken for PCR2. PCR2 was performed on all replicates (directly using 25% of the bead-derived amplified cDNA for replicates A and B1) using primers oJBL324+oJBL495 and the same parameters as PCR1, tracking by qPCR and purifying by 1x Ampure XP beads. (We note that usage of primer oJBL495 was in the first version of the protocol. Presence of the Nextera Read 2 handle forced sequencing of the mBC libraries on a separate sequencing run from GEx libraries to avoid priming conflicts on read 2. We recommend using the updated set of primers oJBL529 and associated indexing primers as described for the mEB library scQer preparation to enable sequencing all libraries on the same sequencing run). A final PCR was performed to index amplicons with primers oJBL076 and indexed primers (oJBL496-oJBL498), and the resulting amplicons purified by 1x Ampure XP beads. The CS2 libraries were prepared entirely analogously to poly-dT captured libraries, except with the following primers: PCR1 for replicate B2 (SR38+SR40), PCR2 all replicates (oJBL529+oSR40), PCR3 all replicates (NextP5_index1+ indexed primers oJBL530-oJBL532).

We note that for both mBC and oBC libraries, semi-nested PCR is necessary to obtain a clean amplicon library (multiple non-specific amplification products were visible following the outer PCR, but a highly specific product was obtained following the semi-nested inner PCR).

All libraries were diluted to 2 nM per the Tapestation D1000 HS reading, pooled, and loaded on a NextSeq 500 for paired-end sequencing the following custom conditions: read 1: 66 cycles (no custom primer); index 1: 10 cycles (primers spiked in: oJBL432, oJBL494); read 2: 76 cycles (primers spiked in: oJBL433, oJBL334). oBC libraries were resequenced to improve saturation of the highly complex oBC libraries following: read 1: 34 cycles (no custom primers); index 1: 10 cycles (primer oJBL432); read 2 38 cycles (primer oJBL433). CS2 mBC libraries were sequenced separately, with: read 1: 30 cycles (no custom primer); index 1: 15 cycles, primer oJBL534; read 2: 18 cycles, primer oJBL334. For mBC and oBC libraries, read 1 provided the cell barcode and UMI, and read 2 the reporter barcode (sequenced with custom primers).

<u>1.2 Optimization of reporter RNA capture</u>

Two experiments were performed to quantitatively characterise UMI capture in our system. First, as described above, one of the sample (replicate B) cDNA was split in two prior to amplification. This enabled a direct comparison of the number of UMIs captured with vs. without the addition of reporter specific primers during cDNA amplification (as opposed to relying on the template switching oligo). For both oBC and mBC, we compared the UMI counts across cell barcode-mBC pairs (valid cell barcode

from gene expression data, valid reporter barcode from subassembly) in replicate B1 (with spike in primers in cDNA amplification) and B2 (without spike in primers in cDNA amplification), **Ext. Data Fig. 1d**. For mBC, we found a median 2.0x increase in UMI counts (for mBC/cell barcode pairs with 4 or more UMIs in both replicates, **Ext. Data Fig. 1d**) in replicate B1 compared to B2, suggesting increased captured resulting from spike-in (both replicates had number of reads per UMI much larger than 1, and in fact larger for replicate B2 [median reads/umi =17.9] compared to B1 [median reads/UMI=6.8], such that this difference cannot be attributed to increase sequencing coverage for replicate B2), consistently with the range previously reported in TAP-seq[107]. Performing the same analysis for oBC led to a much larger boost in the number of UMIs captured (45x increase, also not attributable to high coverage of replicate B1, **Ext. Data Fig. 1e**) as a result of the spiked-in primer. This larger difference was expected from the circular nature of barcode: given the absence of 5' end from which template switching can occur from circular RNAs, the initial cDNA amplification (primed from the template switching oligo) effectively could not happen except from the linear oBC intermediates (expected to represent a minor fraction) in replicate B2.

In addition, we tested which of poly-dT vs. capture sequence derived primers captured more reporter mRNA. Our reporter cassette (**Ext. Data Fig. 1a**) harbours capture sequence 2 (CS2) downstream of the barcode, enabling a direct comparison, for the same reporter in the same cell, of the different number of UMIs captured from the two different RT primers. Comparing across valid cell barcodes and mBC pairs with at least one UMI captured from both poly-dT and CS2, we found a median of 15.7x more UMIs captured from poly-dT primers, likely a direct reflection of the higher stoichiometry of these primers on the 10x Genomics beads (**Ext. Data Fig. 1f**). As such, in all mRNA expression quantifications, we use the poly-dT captured number of mBC UMIs. In addition, we note that using artificial poly-A sequence in place of CS1 on the oBC would likely result in a similar boost in capture and complexity from this system (RT primer saturation should not be a problem given successful overloading of fluidic emulsions without observed decrease in capture efficiency[121]).

1.3 Estimating per oBC per cell captured library complexity

The single-cell oBC libraries were highly complex and as result were not sequenced to saturation. UMI count distributions shown in **Fig. 2c**, **Ext. Data Fig. 2b** (and similarly in mEB, **Ext. Data Fig. 4f**) were therefore not a measure of the full complexity of the libraries (median duplication rate, i.e., read counts over UMI counts minus 1, of 8.3% and 8.8% respectively for oBCs in the high count mode [≥12 UMIs]). To estimate the total complexity for each oBC in each cell, we used the maximum likelihood estimator from the zero-truncated Poisson distribution[122], i.e., if for a given oBC with a given cell barcode x:=(reads counts)/(UMI counts), and λ:=(read counts)/(oBC complexity), then $x = \lambda(1-\exp(-\lambda))^{-1}$. Inverting the relation for each cell barcode and oBC pair in high count mode (provided reads counts is not equal to UMI counts), we find a median complexity of 3652 (K562), 2306 (HEK293T), and 1675 (HepG2) UMI per oBC per cell barcode (replicate A). As expected from splitting the cDNA in two, the estimated complexity was essentially halved for replicated B1 (1731 in K562, 1217 in HEK293T, 850 in HepG2).

1.4 Estimating the probability to have multiple integration per cell for one plasmid

In order to estimate the probability that a unique oBC-promoter-mBC plasmid ends up integrating multiple times in a given cell, we simulated the genomic integration process by drawing multiplicity of integration from the empirically observed distribution (assuming no multi-integration events), and drawing oBC-promoter-mBC combination with replacement with frequency taken as the

quantified proportion in our pool (as assessed during the subassembly stage by sequencing the barcodes). We find the probability of multiple integration, regardless of barcode, to be less than 5% in both replicates. This is somewhat higher than expected from the multi-integration probability from a scenario with fixed number of integration sampling exactly equiprobable barcodes (equal <2% under these circumstances). Spread in both MOI (here 2 to 8 integrants per cell interquartile range) and non-even barcode representation (1122 unique oBC-promoter-mBC triplets in our pool, with 10th to 90th percentile of representation in the pool spanning a 10-fold range: 0.00018 to 0.0017), thus contribute to modestly inflate the likelihood of a multi-integration event, which we nevertheless expect to be rare (<5%) from this empirically derived estimate.

1.5 Clonal cell analysis

Identifying clonal cells harbouring multiple genetic payloads from single-cell data is a non-trivial computational problem, even with high signal to noise ratio, in part due to doublets and barcodes multiply represented across different clones. After assessments of existing approaches[42,43,123] and our own attempt (iterative clustering in high dimensional PCA space from oBC expression), we settled on a modification of the heuristic put forth by Wang and colleagues[43], based on one-sided Fisher's exact test, followed by our own addition of custom quality filtering. Details are provided below.

1.5.1 Clonotype identification

We identified high-confidence integration genotypes (hereafter clonotypes) in a two step procedure. First, a raw clonotype identification, followed by a refinement step.

As a first pass identification of clonotypes, we followed Ref[43] and looped through cells (considering cell barcodes assigned to different cell lines from their transcriptome and different replicates separately), assembling a list of clonotypes. Specifically, for each cell, the list of detected oBC was extracted (≥12 oBC UMI, see below for justification for threshold in addition to corresponding to the minimum of the UMI count distribution, **Fig. 2C** and **S3B**). The list of barcodes from the cell was then compared to the oBCs detected in all other stored clonotypes via a one-sided Fisher's exact test, with contingency table given by (number of oBC in cell and clonotype, number of oBC in clonotype but not in cell; number of oBC in cell not in clonotype, number of oBC from library neither in the clonotype nor in the cell). The test serves to assess the probability that random sampling of oBC leads to as much overlap between cell and clone as observed. A 5% Bonferonni corrected ($0.05/(n_{cells}^2/2)$) p-value was used as a threshold to determine whether a cell was a likely member of a clonotype or not. If oBC from the cell did not overlap significantly from any stored clonotypes, the cell was taken as the representative of a new clonotype. If overlap with multiple existing clonotypes was identified, the cell was marked as a likely doublet. Given the number of cells and barcodes in our experiment (promoter series), *bona fide* clones with only two reporters integrated did not meet the stringency threshold of our test and were thus excluded *de facto*. We note that this heuristic takes the set of oBCs detected from the first representative of a clonotype as the set for all cells (no aggregative correction applied), and as such the resulting cell assignments and clonotypes depend on the order at which cells are considered in the loop. To address this, we implemented an additional downstream filtering step, and returned to the problem of assigning cells to clonotypes after the final list of high-confidence clonotypes was determined.

To refine the raw list of clonotypes identified above, we first exclude clonotypes assigned to a single cell. Then, for a given clonotype, we obtained the union of all detected oBCs ≥12 UMI (clonotype oBCs) from cells assigned to that clonotype. For each of these clonotype oBC, the fraction of cells assigned to the clonotype with detection of that oBC was then determined. We then determined the number of clonotype oBC which were detected between 25% and 75% of cells within the clonotype ($n_{25\% \text{ to } 75\%}$), and the number of clonotype oBC detected in more than 75% of the same cells ($n_{>75\%}$). We also stored the maximum fraction of cells with detection of any one of the oBC within a clonotype (max_frac_detect). We found these quantities to be useful to filter out likely doublets and clonotypes with too much barcode overlap from valid and easily distinguishable clonotypes. We retained clonotypes for which $n_{>75\%}$>2 $n_{25\% \text{ to } 75\%}$ and max_frac_detect>0.9. The list of oBC corresponding to a clonotype was then taken as those detected in >50% of cells assigned to that clonotype. Finally, completely nested clonotypes (ones whose set of oBC was a strict subset of another) were eliminated.

1.5.2 Mapping of cells to clonotypes

Using the list of clonotypes and associated oBC (described above), we returned to the complete dataset to assign cells to clonotype (thereby avoiding the issue of cell ordering affecting the outcome). Specifically, we obtained the list of detected oBCs (≥12 oBC UMI) in each individual cell. We then computed two quantities across all clonotypes, 1) f_1:= fraction of oBCs detected in the cell of interest also present in the clonotype, and 2) f_2:= fraction of clonotype oBCs detected in the cell of interest. In words, f_1 tracks possible additional barcodes detected in the cell not associated with the clonotype (e.g., doublets), while f_2 monitors possible dropouts. For each cell, the top clonotype was taken as the one with the largest f_1, and the associated f_2 was also retained. Cells were assigned the status of a high confidence singlet if f_1>0.975 and f_2>0.5. Hence, we stringently filter out possible doublet (require high f_1), but remain loose on possible dropouts (allow for low f_2, compared to performance, see below). Cells with f_2≤0.5 were considered missed clonotypes.

Through this procedure, we obtained a high proportion of cells assigned as singlets to high-confidence clonotypes (replicate A: K562 67%, HEK293T 66%, HepG2 75%; replicate B1: K562 61%, HEK293T 58%, HepG2 55%), and substantial fraction of the non-singlet cells had an MOI of 2 or lower (replicate A: K562 82%, HEK293T 63%, HepG2 61%; replicate B1: K562 61%, HEK293T 54%, HepG2 30%). Hence a high proportion of missed clonotypes came from low MOI cells and the high stringency of our p-value threshold. The number of clones was somewhat lower than estimated going in the bottleneck, possibly as a result of clonal competition. HepG2 in particular displayed a more severely bottlenecked population (**Ext. Data Fig. 3a-b**), in line with the longer time necessary for those populations to expand (slow growth was observed at the low plating density). Final clonotypes with cell assignments are listed in **Supp. Data 2**.

Final assignments (with clonotypes with 3 or more cells assigned) were displayed on oBC expression space UMAP (**Ext. Data Fig. 3a-b**) using Seurat (Normalisation method “RC”, PCA run on variable features with 100 principal components, UMAP with n.neighbors=10 on top 50 PCs).

1.5.3 Systematic oBC dropout analysis (precision-recall)

The high-confidence clonotypes identified through the consensus of co-detected barcodes served as an approximate ground truth to systematically assess the detectability of oBC in our assay. Specifically, for all clonotypes (with 3 or more cells assigned) and singlet-assigned cells as described above, we computed for different oBC UMI count detection threshold the number of true positives (TP:= number of oBC detected in cell also in the clonotype), false positives (FP:= number oBC detected in the cell not present in the clonotype), and false negatives (FN:= number of oBC in the clonotype not

detected in the cell). At each oBC threshold, the false discovery rate was taken as FDR:=sum(FP)/(sum(FP)+sum(TP)), and the false negative rate FNR:=sum(FN)/(sum(FN)+sum(TP)), where the sums are over all cells and clonotypes. Results stratified by cell lines are shown in **Fig. 2h** (stratified by replicates: **Ext. Data Fig. 3e-f**). Direct representative oBC count distributions are shown in the count matrices to two typical clones shown in **Ext. Data Fig. 3c-d**. In order to prioritise stringency, we selected a UMI of 12 as threshold for the expression analysis presented throughout (different threshold for experiment in embryoid bodies, see below). We note that given the loose stringency of our threshold for assignment (tolerating cells with up to 50% dropout in oBC), this analysis should be relatively unbiased given that the FNR is in the few percent range at the threshold of 12 oBC UMI.

1.5.4 Analysis of reporter barcode expression variability across clones

In addition to providing assessment of oBC dropout, clonal cells present an opportunity to measure variability in the number of captured reporter mRNAs, while controlling for possible positional effects. For each singlet-assigned cells to high confidence clonotypes, the count distribution of oBC UMI and mBC UMI (GEx normalised) corresponding to the integrated reporter cassettes was obtained (e.g., **Ext. Data Fig. 3g-h** for two example clones). The mean across cells assigned to clonotypes and standard deviation in these quantities was determined (analysis restricted to clones with >4 cells assigned to allow for a robust assessment of the standard deviation). The coefficient of variation (standard deviation over mean) was displayed as a function of the mean (**Ext. Data Fig. 3i**), showing scaling close to the limit set by Poisson counting even for some of the highly expressed promoters, and typically much lower than one. This provides direct evidence that when controlling for positional effects and conditioning on presence of the reporter by orthogonal means (here with oBC detection), single-cell measurements can be highly precise.

To assess the proportion of variance attributable to positional effects vs. other technical and biological factors, we used the law of total variance to decompose in mBC UMI variability. For each separate cell line and promoter, we computed the variance of the mean mBC UMI per clone-reporter pair (explained variance) and the mean variance of mBC UMI across clones-reporter pairs (unexplained variance). We find the proportion of unexplained variance to be (average of replicates A and B1): EEF1A1p K562=0.46; HEK293T=0.37, HepG2=0.37; Pgk1p K562=0.44, HEK293T=0.73, HepG2=0.60; UBCp K562=0.33, HEK293T=0.41, HepG2=0.55. The reporter values in the main text are the average over the cell lines.

Variation of the mean expression for the different promoters across clonotypes also provided estimates for the magnitude of genomic context positional effects. We found restricted variability for most promoters, although with evidence for cell-type-specific differences (interquartile fold-change range, for all promoters listed from K562, HEK293T, and HepG2: UBCp: 2.1, 2.2, 2.4; Pgk1p: 5.9, 4.8, 7.2; EEF1A1p: 1.5, 1.5, 4.1), somewhat smaller than the positional effects observed from the Pgk1 promoter in mES cells[45] which had an observed fold-change interquartile of ≈8, suggesting that positional variegation arising from local the epigenetic environment might be partially mitigated by presence of insulators (core cHS4[48]) in our construct (**Ext. Data Fig. 1a**). See **Supp. Fig. 1** for a direct test confirming the importance of the cHS4 sequences.

Clonal analysis also allowed us to compare the distribution of CV across clones for the raw UMI counts, and the GEx normalised UMI counts. We found small but consistent decreases in variability both for oBC (median GEx normalised CV/raw CV = 0.80) and some promoters (UBCp and EEF1A1p: median GEx normalised CV/raw CV=0.85, no difference for, the no promoter, minimal, and Pgk1p promoters), justifying our use of this normalisation in our quantification of reporter mRNA.

2 Profiling developmental cis-regulatory elements in mouse embryoid bodies

2.1 Optimization of high multiplicity of integration with piggyBac in mESCs

As described above, we used co-transfection of selectable carrier transposon to boost multiplicity of integration following previous successful reports[59,60] of the procedure. Importantly, to prevent any bias on expression of the integrated reporters associated with developmental CREs, we leveraged orthogonal selection modalities not associated with the CREs (puromycin and red fluorescent protein, not green fluorescent protein). We directly confirmed the increase in MOI by comparing qPCR-estimated cargo DNA doses from genomic DNA of cells at 11 days post transfection with and without puromycin selection. The qPCR was performed as follows: gDNA extraction with DNeasy, dilution to 100 ng/uL, per well reaction with 5 uL 2x PowerUp master mix (Thermo Fisher, cat. no. A25741), 2 uL 10 mM Tris 8, 1 uL 5 uM forward+reverse primer pair, 1 uL 100 ng/uL gDNA. Primer pairs used: GFP: oJBL039+oJBL040, puromycin cassette: oJBL043+oJBL044, *Tfrc1* (endogenous locus for normalisation): oJBL276+oJBL277. We observed on average a 4.0 to 7.0-fold increase in MOI (with vs. without puro selection, cargo dose with per-sample normalisation from endogenous locus) across three biological replicates (with 10% co-transfection of puromycin containing cargo). We note that in our hands, other approaches used to optimise MOI (selecting on higher dose of puromycin, tuning relative and absolute concentration of transposon and transposase, selection on top 10% GFP intensity) did not improve MOI to the same extent as this co-transfection method. Notably, by adding a second selection round on mCherry positive cells on puro selected expanded cells (mCherry plasmid co-transfected at 1% of the cargo DNA), we saw a further increase in MOI in replicate 2B (median MOI ≈20 for replicates A and B, and up ≈50% to median ≈30 for replicate 2B, **Ext. Data Fig. 4c**) suggesting further optimization might be possible to increase median MOI beyond what has been achieved and boost power. We note that transfecting more cells might then be necessary to avoid extensive bottlenecking (already some detectable through clonal analysis, see below, even for the non explicitly bottlenecked populations replicates A and B).

2.2 scATAC-seq on mEBs

2.2.1 Experimental method

Single-nuclei preparation for scATAC-seq were prepared from day 21 mEB as follows: At day 21 of mEB cultures, mEBs (two 10 cm suspension plates) are collected into a 50 mL conical tube and washed 2x with 1x PBS (without Ca^{2+}, Mg^{2+}). After consecutive PBS washes, mEBs are treated with 1.5mL of 0.25% trypsin and incubated in 37C bath with gentle agitation (steady concentric swirls in 50mL conical tubes) for 3 minutes. For further dissociation, mEBs are then gently triturated 10 times with a P1000 pipette and again incubated at 37C for 3 minutes with gentle agitation. After second incubation, mEBs are gently triturated 10 times with a P1000 pipette. Trypsin digestion is inactivated with CA medium and cells filtered to single-cell suspension through a 100 um filter into a new 50 mL conical. Single cell suspension was counted, and cells spun down at 300 g for 5 minutes. After removing supernatant, wash 1x with 1 mL of 1x PBS + 0.04% BSA and gently pipette mix 5x. Transfer to a 1.5 mL tube and spin at 300g for 5 min at 4C. Wash again with 1mL of 1x PBS + 0.04% BSA. Again, spin at 300 g for 5 min at 4C, and proceeded with 10x Genomics "Nuclei isolation for Single Cell

ATAC Sequencing" protocol (V1), with two biological replicates (different mEB differentiation) each with two lanes of 10x (four reactions total).

2.2.2 Processing of scATAC-seq data

Fastq files were generated by running makefastq. Fastq files were then processed to fragment files using 10x Genomics cellranger count (cellranger-atac-cs version 1.2.0, reference = refdata-cellranger-atac-mm10-1.2.0), which were processed through the ArchR pipeline[124]. Arrow files were created with function createArrowFiles (minTSS=4, minFrags=1000). Two different double scores were computed with function addDoubletScores (LSI based: k=10, knnMethod="LSI", LSIMethod=1); UMAP based: k=10, LSImet=1, UMAPparam: n_neighbors=40, min_dist=0.4, metric="euclidiean"). In addition, we used AMULET[125] (from its v1.0-beta version, running function ATACDoubletDetector.py and adding as problematic region a union of the ENCODE excluded list, segmental duplications, simple repeats, repeat masker, and microsatellites from mm10 obtained from UCSC). Nuclei were then filtered for: TSS enrichment >8 and >1995 fragment counts. Following dimensional reduction (addIterativeLSI: useMatrix = "TileMatrix", name = "IterativeLSI", iterations = 2, varFeatures = 100000, dimsToUse = 1:30), and clustering (AddClusters: reducedDims = "IterativeLSI", method = "Seurat", name = "Clusters", resolution = 0.5), doublets were stringently removed by inspecting distribution of fragment counts, doublet scores (ArchR derived), and AMULET doublet scores per clusters. All nuclei from clusters with anomalously high doublet scores across metrics were removed. In addition, individual nuclei with either >17782 fragment counts, LSI doublet score > 0, UMAP doublet score > 0, or AMULET score > 0.3 (thresholds assessed from the distribution of anomalous doublet clusters) were filtered out as likely doublets. In the end, 31% of nuclei were removed with these filters, leaving 46408 nuclei passing quality filters (20329 nuclei from replicate 1, 26079 nuclei from replicate 2).

The resulting filtered nuclei were dimensionally reduced and clustered (same parameters as above), leading to 9 clusters with >200 nuclei. Clusters with highly correlated accessibility (determined from pseudobulk averaging over cells in cluster) over all peaks (R^2 on log-transformed accessibility >0.55) and proximal in the low-dimensional projection were merged. The intermediate endoderm cluster (connecting the visceral and parietal clusters) was kept separate to avoid diluting the signal from the two otherwise well-delineated extraembryonic endoderm clusters. The final 7 clusters are depicted in **Ext. Data Fig. 4e** (see later section for integration/annotation). scATAC pseudobulk pileup traces (e.g., **Fig. 3a**, **4b**) were generated by first the normalised data using ArchR's function groupRegionSumArrows. A subset of all cell-type pseudboulks was shown due to space limitations in figures.

2.3 Prioritization of developmental loci and putative CRE selection

In order to select regulatory elements possibly implicated in control of gene expression in our system, we prioritized loci on the basis of a number of criteria. First, we identified highly differentially expressed genes within neuroectoderm, endoderm, mesoderm, and pluripotent clusters from mEBs (SGR and SD, in preparation unpublished data) using Seurat FindMarkers function, retaining genes with at least 25% detected expression in the respective clusters, and either a fold-change in expression >1.6x or a fold-change in fraction of cells detected with expression >2. We then identified all peaks from the scATAC data with score (as generated by ArchR) >20 within 100 kb of the TSS of each gene. The resulting gene-peak data table was then augmented with information about the ATAC peaks (accessibility in cell-type cognate to the differential expression, fold-change in accessibility, average phyloP score[108], distance to the nearest gene, overlap with ccRE[9], orthology to a reciprocal human

ccRE). Genes were retained for further assessment if their ±100 kb neighbourhood included 3 or more highly accessible and differentially accessible peaks (top 90th percentile) in the cell-type cognate to the differential expression. In addition, loci harbouring one or more non-exonic peaks with evidence of conservation (average phyloP>0.75 or presence of orthologous human ccRE) and within 50 kb of a very highly differentially expressed genes (>3.8 fold-change in expression or >7.7 fold-change in fraction of cells with detected expression) were retained. Finally, loci with one or more non-exonic peaks with either: 1) strong conservation (>2 average phyloP score) and high differential accessibility (>15x fold change), or 2) evidence of conservation (average phyloP>0.75 or presence of orthologous human ccRE) and very high differential accessibility (>15x fold change), were retained. Filtering on these different criteria led to a list of 89 loci, which were manually evaluated. To arrive at our final list of 22 loci (**Supp. Data 1**), genes were ranked by the number of peaks satisfying the above criteria (conservation and differential activity), and examples from parietal endoderm, neuroectoderm, and mesoderm cell-types with overall low gene density (to avoid the possible complication of neighbouring gene regulation) were selected.

Following loci prioritisation, the final set of putative CREs selected was any peak within 100 kb of the annotated differentially expressed genes above $>9.4\times10^{-3}$ accessibility (normalised by TSS reads, average in all cells annotated to differential-expression-cognate cell-type) reproducibly in both scATAC replicates, leading to 206 regions. A strongly differentially accessible peak 2 kb upstream of the gene *Tubb2b* TSS (*Tubb2b*:ch13_2580) which had fortuitously not passed our thresholding criteria was included. We finally added the 4 constituents of the core Sox2 control region as CREs of interest to include, for a total of 211 elements. Robust primers for PCR-cloning could be designed for 209/211 of them (primers: **Supp. Data 11**, list of CRE sequences and positions: **Supp. Data 1**), see section below, and 204/209 were sufficiently represented in our constructed scQer libraries to allow for quantification (**Fig. 4a**, **Ext. Data Fig. 5h**, **Supp. Fig. 2b-c**).

2.4 Cloning details

2.4.1 Recloning of oBC-mBC backbone plasmid

Doubly barcoded backbone plasmid (p025) was recloned in order to increase the complexity of the barcode pairs. Briefly, new barcodes were appended by amplifying the region between the oBC and mBC with primers with random (5'VNNVNNVNNVNN for the oBC) primers (oJBL513+oJBL514) with Kapa HiFi (15 cycles). Following 1.5x Ampure XP beads clean up, the barcoded insert was further amplified (15 cycles Kapa HiFi) to append homology arms for Gibson assembly (oJBL515+oJBL516). The final insert was PAGE purified. The insert-compatible backbone was reconstructed from two PCR products from p025 (oJBL524+oJBL527, oJBL526+oJBL525) of about 2.6 kb each (agarose gel purified). The three pieces were then combined by Gibson assembly, and electroporated in *E. coli* (C3020, NEB). Full complexity of the library was maintained, and estimated to be ≈1M clones by colony counting transformants.

2.4.2 oBC-mBC subassembly

Following re-cloning of p025, the oBC-mBC pairs in the library were obtained as described for the promoter series experiment by a single step of PCR (primers oJBL337+oJBL345) to append handles for sequencing (on NextSeq 500, with library structure: read 1 oJBL346 (oBC, 30 cycles); index 1 oJBL347 (library index, 15 cycles); read 2 oJBL334 (mBC reverse complement, 18 cycles); index 2 oJBL348 (oBC reverse complement). Pre-processing also was carried out as described, resulting in

oBC-mBC pairs with each associated with a read count. Given the complexity of the library (unsaturated), a cutoff of at least 5 reads was applied to retain oBC-mBC pairs (1.2M pairs). To mark possible non-uniquely paired barcodes, we computed the proportion of read counts to each oBC and mBC from a given oBC-mBC pair. oBC-mBC pairs with oBC or mBC with read counts proportion belonging to the pair of less than 95% were marked as likely non-unique (78.1% likely unique pairs by this criterion).

2.4.3 Construction of EEF1A1p-mCherry transposon plasmid

To obtain an orthogonal selection for co-transfection to boost MOI (see below), we cloned a constitutively expressed red fluorescent protein into the piggyBac transposon. Briefly, p001 (piggyBac transposon backbone) was digested with XbaI and EcoRI (NEB), and size selected on agarose. The EEF1A1 promoter was amplified from p003 using primers oJBL536+oJBL537, and mCherry was amplified from a puro-mCherry containing plasmid with primers oJBL538+oJBL539 with Kapa Robust. The resulting fragments were size selected on agarose, and combined with the digested backbone by Gibson assembly, and transformed. The final plasmid taken from an individual colony was confirmed by Sanger sequencing and used for co-transfection.

2.5 Single-cell reporter libraries preparation and sequencing

The three single-cell libraries (gene expression GEx, oBC, mBC) for the mEB experiment were prepared as described for the benchmarking experiment in human cell lines (with no splitting of the cDNA, spike-in at 0.5 uM of primers oJBL246 and SR38 at first cDNA amplification to enrich for the reporter barcodes), with the following modifications.

oBC libraries were prepared the same way as for human cell line experiments, but with different P7-indexed primers for the final inner PCR (oJBL501-oJBL506). In addition, to avoid loop-the-loop products in the oBC libraries (anecdotally decreasing sequencing quality), the lowest band in the circularized ladder amplicons (see e.g., **Ext. Data Fig. 1b**, **Ext. Data Fig. 1j**) was size selected on PAGE for each library and used for sequencing.

Given the limited added value of CS2 capture for mBC (**Ext. Data Fig. 1f**), only the poly-dT captured libraries were generated for the mBC, with the following primers for the two rounds of PCRs (following initial cDNA amplification). PCR2: oJBL324+oJBL529. PCR3: oJBL076+ P7-indexed primers (oJBL530-oJBL533).

GEx, oBC, and mBC libraries were sequenced at the same time for replicates A/B on NextSeq500 (read 1: 28 cycles, no custom primers; index 1: 10 cycles, spike in primers oJBL432, oJBL534; read 2: 54 cycles, spike in primers oJBL433, oJBL334). The three libraries for replicate 2B were similarly sequenced, except with 8 cycles on index1 and 56 cycles on read 2. The oBC libraries for replicates A and B were re-sequenced as part of a NextSeq2000 run, with 28 cycles on read1, 10 cycles on index1, and 20 cycles on read2, with primers SR40+oJBL433 in well 1, and primer oJBL432 in well 2.

2.7 Single-cell data processing

2.5.1 Quality filtering from gene expression libraries

Fastq files were generated using the makefastq command from cellranger (v6.0.1), and the gene expression count matrices were then generated with cellranger count, with transcriptome reference mm10-3.0.0. Raw count matrices were then imported as a Seurat[96] object (filtering genes expressed in less than 3 cells, and cell barcodes with less than 50 genes measured). Cell barcodes in the high total UMI mode with low mitochondrial RNA proportion were filtered as likely *bona fide* cells (fraction of mitochondrial UMI >1% and <15%, total gene expression UMI > 400 for samples from replicates A, B, and 2B lane1, and >1000 for 2B lane2, which was fortuitously sequenced more deeply). The filtered count matrices were then used to evaluate doublet scores using scrublet[98] (scrub_doublets command, 30 principal components, mean_center=true, normalize_variance=true), and cell barcodes with doublet score > 0.3 (separating the two modes of the simulated doublet distribution from scrublet) were filtered out. Datasets from all replicates were then combined in a single Seurat object, dimensionally reduced and clustered (NormalizeData, normalization.method= “LogNormalize”, scale.factor=10000; FindVariableFeatures with selection.method = “vst”, nfeatures=1000; ScaleData with all genes as features; RunPCA with identified variable features and 100 principal components; FindNeighbors, dims=1:50; FindClusters, resolution=0.2; RunUMAP, dims=1:50, n.neighbors=50) without batch correction given the good correspondence between replicates (**Ext. Data Fig. 5e**). The cluster identities were taken as categories for cell-type expression testing (see integration section below).

The following additional quality filtering steps were applied to retain high confidence singlet cells. Clusters comprising less than 1% of cells were considered likely doublets/artifacts, and corresponding cells were removed. Cells members of each cluster identified were separately sub-clustered with the same procedure as above (except resolution 0.5 in FindNeighbors). Any sub-cluster with a median doublet score above 0.15 was deemed composed of likely doublets, and corresponding cells were removed. Cells with anomalously high gene expression UMI counts were removed (with technical lane specific thresholds: >10k for A.1, >9k A.2, >12k B.1, >9k B.2, >15k 2B.2, no anomalous cells in 2B.1). Finally, cells with an estimated MOI > 200 (roughly corresponding to the top 0.1% of the distribution, MOI estimated through oBC UMI > 10, see below) were filtered out. In the end, n=43799 cells passed all these quality filters (12859 replicate A, 15422 replicate B, 15518 replicate 2B).

2.5.2 mBC and oBC libraries

Raw data was processed in the same way as for the human cell line promoter experiment to obtain a table of barcodes (mBC or oBC) with read and UMI counts per cell barcode. For oBC libraries of replicates A and B, two sequencing runs (for higher depth) were combined into one by concatenating their fastqs (trimming to the same read size) prior to running in cell ranger for the first processing step. Only cell barcodes passing the QC filters from the GEx analysis were retained in the final count tables.

2.5.3 Single-cell quantification of reporter expression

A similar approach as the promoter series experiment was taken to quantify expression in individual cells. For a given CRE of interest, all cells with associated oBC (in the list of valid oBC-CRE-mBC triplets) captured at >10 UMI counts were retained. The associated mBC UMI counts, in the respective cells, was then divided by the depth normalised GEx total UMI counts, and multiplied by the mean normalised GEx total UMI count across all cells (as before, to on average have a normalisation factor with a mean of 1 to not systematically distort the scale of UMI counts while correcting for systematic factors such as cell sizes and overall efficiency of in-emulsion reverse

transcription). To correct for slight differences in coverage between replicates, total GEx UMI count across cells was taken per replicate, and used to normalise (by direct division, under the valid assumption that the GEx libraries are far from saturation) the GEx UMI count in individual cells (different scaling factor per replicate). In cells in which multiple reporters (with different oBC-mBC pairs) corresponding to the same CRE were detected (via the oBC), the average across this normalised mBC UMI count was taken to obtain the per-cell estimate (for displaying the single-cell CRE activity maps). For statistical tests and quantification, the average was taken across integration events as opposed to across cells (not first averaging internally within each cell, and then averaging over all cells, instead directly averaging over all integration events, such that each detection event carries the same weight).

2.6 Bulk MPRA (CREs, mEB time series experiment)

2.6.1 Library preparation and sequencing

Bulk MPRA libraries for the CRE time series were generated similarly as described for the human cell lines promoter series experiment (**Fig. 2a**) with the following modifications. Genomic DNA and RNA were extracted with the AllPrep kit (Qiagen). 40 samples (different replicate/batch/time points) were processed overall, comprising 13 samples for replicates A and B across 12 time point (day 0, 4, 6, 10, 12, 14, 16, 18, 20, 21; with two technical replicates for day 16) and 14 samples for rep2B (two technical replicates for day 0, 8, 12, 16, 18, 20; one replicate each for day 4, 6). From each sample, 2 libraries (1 gDNA-derived, 1 RNA derived) were constructed, for a total of 80 libraries. Libraries were prepared in three batches (batch1: replicates A and B, days 0, 4, 8, 12, 16; batch2: replicates A and B, days 2, 6, 10, 14, 16, 18, 20, 21; batch3: all samples from replicate 2B). For RNA, DNase treatment was applied to the first batch, but was found to be unnecessary (comparing to no reverse transcription controls), and was consequently not performed on the other two batches. As before, reverse transcription used primer oJBL358. The first PCR using the cDNA for the RNA-derived libraries was with primers oJBL077+oJBL039. The first PCR from genomic DNA was with primers oJBL039+oJBL358. The second PCR (performed on both RNA and gDNA derived samples) using primer oJBL077 and a set of indexed primers (oJBL359-oJBL366, oJBL437-oJBL448, oJBL555-oJBL564).

Each preparation batch was sequenced separately. Batch 1: Nextseq500; read 1: 28 cycles, primer oJBL369 (mBC forward); index 1: 8 cycles, primer oJBL435 (UMI); read 2: 43 cycles, primer oJBL371 (mBC reverse); index 2: 6 cycles, primer oJBL370 (P5-index). Batch 2: Nextseq2000, same set of primers (well 1: oJBL369+oJBL370, well2: oJBL370+oJBL435), read 1: 28 cycles, index 1: 10 cycles, read 2: 20 cycles, index 2: 6 cycles. Batch 3: Nextseq500: read 1: 18 cycles, index 1: 10 cycles, read 2: 20 cycles, index 2: 6 cycles.

2.6.2 Data processing and quantification

Data was pre-processed in the same way as for the human cell line bulk MPRA. Briefly, following demultiplexing with bcl2fastq (v2.20), mBC reads were trimmed to their expected lengths (15 nt) with seqtk's trimfq. mBC reads were then joined/error-corrected using PEAR (v0.9.11, options -v 15 -m 15 -t 15). The correctly assembled barcodes were then reformatted and merged with the (pseudo-)UMI read using custom python scripts, resulting in a list of mBC-UMI paired reads. The read counts for each mBC-UMI pair was determined, and a final pileup performed to generate a table of total UMI and read counts for each mBC.

From these raw mBC UMI counts, only mBC sequences from valid oBC-CRE-mBC triplets (including the promoter series) from our subassembly (34121 mBC total, 33001 from CREs, and 1120 from exogenous promoters) were retained, and appropriate metadata information (sample, time point, RNA/DNA, etc) was appended. DNA UMI counts for each barcode (across each sample) were then normalised for sequencing depth dividing by the summed UMI counts from that sample. RNA UMI counts were similarly normalised. To obtain an activity for each CRE, we first only included well-represented mBC (requiring >20 DNA reads) for quantification. Then, we 1% winsorised DNA and RNA normalised UMI counts (to mitigate extreme outliers) across all barcodes from a given CRE. The winsorised normalised UMI counts were then summed across mBCs (for a given CRE) for DNA and RNA, and the ratio was taken to be the activity of the CRE in that sample. For a given CRE, the averaged activity from all samples from two adjacent time points (days 0 & 2, 4 & 6, 8 & 10, 12 & 14, 16 & 18, 20 & 21) were shown in **Ext. Data Fig. 9**, with error bar the standard deviation of the mean across these samples.

As a statistical test of activity, we used a Wilcoxon rank-sum test (one-sided). At each aggregate time point, the activity from all samples from the CRE of interest was compared to the activity of basal expression controls (minimal and no promoter) from all samples/time points. The resulting p-values (across all time points and CREs) were Benjamin-Hochberg corrected. Activity displayed as significant when the false discovery rate was below 1% (**Ext. Data Fig. 6c**, **Ext. Data Fig. 9c**, summary of quantification in **Supp. Data 7**). The fold-change in activity over time (**Ext. Data Fig. 9b**) was taken as the mean activity for day 20.5 (all samples from days 20 and 21) over day 1 (all samples from day 0 and 2).

2.7 Single-cell data integration

2.7.1 Integration between scRNA-seq and Pijuan-Sala et al in vivo scRNA-seq

We compared our day 21 mEB (containing scQers) scRNA-seq data to available *in vivo* data from mouse development[61] (E6.5 to E8.5) to annotate identified clusters from low dimensional projections of our data. Samples spanning time points E6.5 to E8.5 were obtained (using the R library 'MouseGastrulationData', function EmbryoAtlasData with all samples except ids 11, 22, and 23). The count matrix was extracted together with the metadata, and a Seurat object was created after converting the gene names for compatibility, and merged with the mEB dataset. We performed integration as previously described [126]. Briefly, a list of objects was generated from the merged Seurat object, the two datasets were separately normalised and features identified (NormalizeData; FindVariableFeatures, selection.method="vst", nfeatures=2000). Functions SelectIntegrationFeatures, FindIntegrationAnchors, and IntegrateData were sequentially applied to the list, and the integrated data was then dimensionally reduced via scaling and PCA (ScaleData, RunPCA with 30 principal components). The PCA embedding space from the integrated dataset was used to identify neighbours using a method adapted from[127]. For each cell in the mEB dataset, the top 10 closest distance neighbours in the dataset-integrated PCA space from the *in vivo* dataset were identified, and their cell-type annotation stored. Cell annotation from the *in vivo* data was transferred if >6/10 nearest neighbours had the same cell-type label, and taken as 'uncertain' otherwise. This provided an annotation label for each cell in our mEB dataset. To aggregate the annotation across clusters in the mEB data, we determined the fraction of cells per mEB derived clusters with *in vivo* cell-type annotation, shown in the heatmap of **Ext. Data Fig. 4c**. In that representation, *in vivo* cell types with a maximum fraction across all mEB clusters <5% were not displayed for brevity. Final mEB cluster

annotations were determined by inspection, and coarse-grained clusters (**Fig. 3c**) naturally combined cell-types from the same lineage. One important distinction was the label of pluripotent cells, not present in the *in vivo* dataset given that the earliest time point covered was E6.5. The putative cluster of pluripotent cells was closest to epiblast cells within this constrained label-transfer assignment (**Ext. Data Fig. 4c**), but inspection of key marker genes of naive pluripotency [63,128] such as *Esrrb*, *Dppa3* (**Ext. Data Fig. 4b**) were sharply expressed in that cluster, in contrast to markers of primed pluripotency (*Fgf5*, *Dnmt3b*) which were expressed in other clusters (**Ext. Data Fig. 4b**). These justified our identification of this cluster as pluripotent cells.

Performing the label transfer on coarse-grained annotations (grouping all endodermal cells, ectodermal cells, etc.) decreased the proportion of the 'uncertain' label, which in some instances was spuriously created by mEB cells associated with mixed populations from otherwise well-defined lineages (e.g., the multiple different mesodermal cell types). We verified that the final label transfer was robust to the number of neighbours (5 to 20) considered in the PCA integrated embeddings.

2.7.2 Integration between scRNA-seq and scATAC-seq and correlation with in vivo data

The scRNA-seq and scATAC-seq in mEBs was not performed on the same set of cells or as a co-assay (but samples were derived from the same mESC line). We therefore relied on computational approaches to relate the clusters of the low dimensional representations from the two modalities. To that end, we performed unconstrained integration using ArchR[124] function addGeneIntegrationMatrix which uses the functionalities of Seurat[126]. The resulting assignments unambiguously mapped clusters from the RNA to the ATAC (**Ext. Data Fig. 4d**), with some of the finer resolution achievable in the scRNA-seq (e.g., different mesodermal and neuroectodermal clusters) not distinguishable in the scATAC possibly as a result of the fewer number of nuclei sampled from these cell types.

As additional verification for the validity of these cell-type assignments on the scATAC data, we compared the data to available scATAC datasets from mouse embryos at E7.5 and E8.5[53]. We downloaded pileup bigWig scATAC files from all cell types (GEO: accession GSE205117), and generated bigWig pileup from our mEB datasets (ArchR's getGroupBW function, tileSize=50, maxCells=100000, ceiling=10, normMethod="ReadsInTSS"). We then computed the average accessibility across all peaks called by ArchR using UCSC utility function[130] bigWigAverageOverBed. Restricting to the top 25% scoring peaks called in the mEB scATAC dataset (ArchR score >20, corresponding to 65k peaks), when then computed the R^2 on log-transformed peak accessibility in the *in vivo* and mEB datasets across all cell-types/clusters. The overwhelming majority clusters in the mEB scATAC data assigned from the comparison to scRNA-seq had their highest correlations to corresponding *in vivo* cell types: mEB parietal endoderm vs. *in vivo* parietal endoderm R^2=0.77; mEB mesoderm vs. *in vivo* mesenchyme R^2=0.76, vs. Pharyngeal_mesoderm R^2=0.72, vs. Paraxial mesoderm R^2=0.72; mEB neuroectoderm vs. *in vivo* Forebrain, Midbrain, Hindbrain R^2=0.78, spinal cord R^2=0.75; mEB pluripotent/epiblast vs. *in vivo* epiblast R^2=0.86; mEB visceral endoderm vs. *in vivo* ExE endoderm R^2=0.61, vs. visceral endoderm R^2=0.48. Only mEB surface ectoderm had a higher correlation with another in vivo cell-type (highest *in vivo* correlation to gut, R^2=0.67, we note that the label-transfer from scRNA-seq datasets suggests partial recognition of surface ectoderm as gut, **Ext. Data Fig. 4c**), but still had accessibility highly correlated to the expected cognate cluster (second highest correlation R^2=0.60 to *in vivo* surface ectoderm). Taken together, these show the mEBs harbor complex epigenetic states broadly representative of *in vivo* gene regulation.

2.8 Clonal cell analysis

2.8.1 Clonotype identification, refinement, cell assignments, basic metrics, and dropout assessment

Analysis proceeded as described above for the human cell line experiment with minor modifications. First, only oBC associated with CREs (not exogenous promoters) were considered for clonal assignment. That was to minimise the likelihood of spurious doublets being called as a result of the lower complexity of the promoter library (increasing the likelihood of co-integration of the same pair of barcodes in otherwise unrelated clones). The UMI cutoff for oBC detection per cell was set to >10. Other parameters for the procedure (raw clonotype identification with Fisher exact test, clonotype refinement, cell assignment to high confidence clonotype) were as before.

Across the three replicates, the fraction of cells assigned to high confidence clonotype was 4535/12859 (29%, 896 clonotypes) for replicate A, 6854/15422 (53%, 866 clonotypes) for replicate B, and 8406/15518 (54%, 360 clonotypes) for replicate 2B. The mean numbers of cells assigned for these high confidence clonotypes were respectively 5.0, 7.9, and 23.4 for replicates A, B, and 2B, consistent with replicate 2B having been directly bottlenecked. We note that evidence of substantial clonal expansion in the non explicitly bottlenecked replicates (A and B) suggests that our procedure to select high MOI cells (selection on puromycin from ≈5% of plasmid transfected containing the expressed resistance cassette) did severely reduce the complexity of the cell population. While replicate 2B corresponded to a sub-sampling of replicate B (we were not aware at the time of substantial bottlenecking in our population), most clones and cells did not overlap between the two samples (44 clonotypes identified in both samples, or 44/866 of clonotypes comprising 499/6854 clonotype-assigned cells for replicate B; 44/360 of clonotypes 2533/8406 clonotype-assigned cells for replicate 2B). Summary of clonotypes and assigned cells can be found in **Supp. Data 6**.

oBC dropout analysis (**Ext. Data Fig. 5i-k**) from the clonotypes and cell assignment was performed as described for the human cell line experiment.

2.8.2 CRE expression pattern across clones

From the assignment of cells to high confidence clonotypes, we sought to characterize how the expression of CREs varied across clones, with the assumption that different clones correspond to different genomic positions of integration of the reporter driven by the CRE. For each of the 10 active cell-type-specific CREs identified, we obtained the list of high confidence clones harboring at least one reporter integration corresponding to the CRE. In order to obtain sufficient statistical power to estimate expression, we then restricted the analysis to clones with 5 or more assigned cells in both the cell type of expected CRE expression (e.g., pluripotent for *Sox2*:chr3_2007, parietal endoderm for *Gata4*:chr13_5729, etc.) from the analysis over all cells, and 5 or more cells assigned to the rest of cell-types. We then computed the fold-change in mean reporter expression (average normalised mBC UMI) over cells in these two compartments (cognate vs. rest of cells), and calculated the number of clones per CRE for which fold-change was >5. For 9/10 CRE, more than ⅔ of clones retained a >5 specificity (**Supp. Fig. 6**).

2.9 Analysis of features of profiled putative developmental CREs

Various features of the profiled CRE were considered for correlation with cell-type-specific activity. These were determined as follows:

ATAC accessibility: for each peak in each cell, the corresponding read count was normalised by the total number of TSS reads in that nucleus. The average overall cells assigned to a given cluster was then taken as the mean accessibility for the given peak in that cluster. Fold-change in accessibility was taken as that measure of accessibility over the mean accessibility averaging over cells from well-delineated clusters (pluripotent/epiblast, neuroectoderm, mesoderm, and extraembryonic endoderm/parietal), such that for example fold-change accessibility for parietal endoderm was: mean accessibility in parietal endoderm divided by mean accessibility in all cells from epiblast/pluripotent, neuroectoderm, and mesoderm clusters. The visceral and intermediate parietal endoderm clusters were not considered for the fold change computation to not have cells from the same lineage be included in the comparison, which could have artificially decreased the effect size for parietal endoderm.

Pseudotime opening: In the absence of a time series scATAC-seq dataset, we considered pseudo-time trajectories[109]. First, scATAC data was clustered at higher resolution (resolution=2) using ArchR's addClusters function. A trajectory from pluripotent to parietal endoderm, passing through these more highly resolved clusters, was then defined and created with the addTrajectory function. Accessibility information along the trajectory was extracted with function getTrajectory (useMatrix="PeakMatrix", log2Norm=TRUE, and smoothWindow=10). Pseudotime smoothed accessibility values for each considered peak (distal parietal endoderm) was then obtained. To estimate the pseudotime at which a peak became accessible, we fit an exponential sigmoid (logistic function, using SSlogis and nls in R) to each accessibility vs. pseudotime trace. The pseudotime at which the sigmoid reached 20% of its maximum from baseline was selected as the heuristic value to compare opening times of the different peaks.

Evolutionary conservation: to assess evolutionary conservation, we calculated the average phyloP [108] score (mm10.60way.phyloP60way.bw) over ArchR-defined 500 bp ATAC peaks using function bigWigAverageOverBed[130] similarly to previous assessment of non-coding element conservation[9].

For single-feature classifiers, a simple thresholding on the feature was used to generate the ROC curves (**Supp. Fig. 7b**). To combine cognate ATAC accessibility and number of Gata4 binding sites, we used scikit-learn[131] function LogisticRegression with an l1 penalty (mean=0 and standard deviation=1 input variables) and the roc_curve function to compute the performance metric.

3 Pol III driven circular vs. linear barcode MPRA experiment

3.1 Cloning of plasmids

The Tornado cassette was first cloned in a piggyBac transposon. The piggyBac cloning dock p022 was digested with BbsI (NEB) and the U6-Tornado-Broccoli insert excised from the pAV-U6+27-Tornado-Broccoli plasmid[34] (Addgene #124360) with BamHI and XhoI (NEB) digestion. Both backbone and insert were purified by agarose gel extraction (Zymoclean Gel DNA recovery kit, Zymo Research), the fragments combined by isothermal assembly (HiFi NEBuilder, NEB) into plasmid p051, and transformed in *E. coli* (NEB, C3040H). A single clone was selected and the plasmid confirmed by Sanger sequencing. A truncated version of the Tornado cassette, excluding the 5' and 3' portion of the ribozyme not overlapping with the final circular RNA sequence, was generated by digesting p051 with XbaI and SalI, and combining with gblock linear_TB_CS by isothermal assembly. The resulting plasmid, p052, was transformed in *E. coli* (NEB, C3040H). A single clone was selected and the plasmid confirmed by Sanger sequencing.

To generate complex libraries barcodes, barcoded inserts (5'VNNNVNNNVNNNVNNN) with downstream capture sequence 1 (CS1, 5'GCTTTAAGGCCGGTCCTAGCAA) were amplified from ultramer uJBL519. For circular barcodes, primers oJBL520+oJBL521 were used for amplification, the resulting product PAGE purified, and inserted in NotI+SacII digested p051 purified by agarose gel extraction by isothermal assembly. The plasmid library, p053 (**Ext. Data Fig. 1h**), was concentrated and eluted in water (Zymo Clean and Concentrator, Zymo research), and electroporated in *E. coli* (NEB, C3020) following manufacturer's instruction. A similar procedure was taken for linear barcodes, except that primers oJBL522+oJBL523 were used to amplify uJBL519, and integrated in NotI+SacII digested p052, resulting in plasmid library p054 (**Ext. Data Fig. 1i**). Of note, both circular and linear barcode constructs were compatible for reverse transcription and amplification from the same primers for library preparation to minimise biases. Following outgrowth post electroporation, a dilution series was plated to assess library complexity, and populations estimated at 50k clones were expanded, and resulting plasmids libraries purified (ZymoPure II plasmid Midiprep kit, Zymo Research), and further concentrated to 1 µg/µL by isopropanol precipitation.

3.2 Transfection, cell culture, and cell harvesting

2.75 µL each of plasmids libraries p053 and p054 were mixed to 0.6 µL (0.3 µg) of SBI super piggyBac, and transfected 2.5 M of exponentially growing K562 cells in duplicates using a Nucleofector following manufacturer's protocol for K562 (kit V4XC-2024, Lonza BioResearch) in duplicates. After two weeks of exponential growth with ⅕ split every two days to allow for dilution of unintegrated plasmids, cells were harvested in exponential phase (<0.75 M/mL), and methanol fixed. Briefly, cells were pelleted at 500 g for 5 min, washed with ice cold 1x PBS to 2 M/mL, pelleted at 500 g for 5 min, resuspended to 15 M/mL, and ice cold methanol was added drop by drop to 80%. Aliquots of 4 M fixed cells were stored at -80C until DNA or RNA extractions.

3.3 Massively parallel reporter assay library generation and sequencing:

Bulk MPRA for Pol III barcodes proceeded similarly as described before. Fixed cells were split, and genomic DNA was extracted from methanol fixed cells using the DNeasy kit (Qiagen), and RNA was extracted from cells using TRIzol LS (Thermo Fisher), following manufacturer's instructions in both cases.

Amplicon libraries from DNA were generated in two steps of PCR amplification with Kapa HiFi (Roche). For genomic DNA, 500 ng of input was used, and for plasmids (to map barcodes present in both constructs), 3 ng was used. For low-cycle number PCR1, 500 ng of DNA was mixed with 50 µL 2× Kapa HiFi master mix, 5 µL 10 µM oJBL246, 5 µL 10 µM oJBL424, and water to 100 µL. Cycling parameters: 1 min at 95C, and 4 cycles of: 20 s at 98C, 20 s at 60C, 30 s at 72C, followed by 4C hold. Primer oJBL424 contains 10 random Ns to serve as a pseudo-UMI (hereafter referred to as UMIs for brevity) to correct for PCR jackpotting. Reactions were cleaned up with Ampure XP beads (Beckman Coulter) at 1.75×, and eluted in 20 µL of 10 mM Tris 8. Illumina adapters and sequencing indices were appended through PCR2, with 4 µL of the eluate from PCR1 taken as input, and 25 µL 2× Kapa HiFi master mix, 0.25 µL 100× SYBr green, 2.5 µL 10 µM oJBL076, 2.5 µL 10 µM indexed primers (DNA rep1: oJBL501, DNA rep2: oJBL502, plasmid p053: oJBL427, plasmid p054: oJBL504), and water to 50 µL. Libraries were amplified with tracking by qPCR with: 1 min at 95C, and cycles up to the qPCR inflection point (typically 15-17 cycles) of: 20 s at 98C, 20 s at 60C, 30 s at 72C. Libraries were then cleaned up with Ampure XP beads at 1.75×.

Amplicons libraries for RNA were obtained by first DNase treating the RNA (5 µg RNA, 2 µL TURBO DNase [Thermo Fisher], 2 µL 10× buffer, and waster to 20 µL, incubated at 37C for 30 min, cleaned up with RNA clean & concentrator [Zymo Research], and eluted in 11 Tris 7 10 mM), and taking 1 µg of DNase treated RNA to reverse transcription. Briefly, 2 µL (500 ng/µL) RNA was mixed with 2 µL 1 µM oJBL424, incubated at 65C for 5 min, and placed on ice. 15 µL of reverse transcription master mix was then added (4 µL 5× FS buffer, 1 µL 0.1 M DTT, 1 µL 10 mM dNTP mix, 8 µL water, 1 µL SSIII [Thermo Fisher]), and the reaction incubated at 55C for 60 min, followed by 70C for 15 min. ¼ of the reverse transcription reaction was then directly amplified for PCR1 (37.5 2× Kapa HiFi master mix, 3.75 µL oJBL246, 3.75 µL oJBL076, water to 75 µL), with cycling parameters: 1 min at 95C, and 4 cycles of: 20 s at 98C, 20 s at 60C, 30 s at 72C, followed by 4C hold. Reactions were cleaned up with Ampure XP beads (Beckman Coulter) at 1.75×, and eluted in 20 µL of 10 mM Tris 8. PCR2 proceeded as for libraries prepared from plasmids and genomic DNA, with indexing primers oJBL508 and oJBL509 for replicates 1 and 2 respectively, and reactions stopped at inflexion point from qPCR tracking (cycle 7). Libraries were then cleaned up with Ampure XP beads at 1.75×. Notably, given the circular nature of the Tornado barcodes, rolling-circle loop-the-loop RT products were prominently visible (at least 4-loops products detectable) at the expected size laddering from repeats of the circular RNA length (**Ext. Data Fig. 1j**). To prevent possible phasing issues on the sequencer, the product of the lowest size, which was the same for both linear and circular barcodes, was purified by PAGE extraction.

Final amplicon libraries were quantified with Qubit dsDNA HS (Thermo Fisher), diluted to 3 nM, run on TapeStation D1000 HS (Agilent) for final quality assessment, and adjusted to final 2 nM based on the Tapestation quantification. Libraries were pooled, loaded as a fraction of a NextSeq500 lane, and paired end sequenced with the following parameters: read1 (barcode forward): 25 cycles with primer oJBL431, index1 (index): 20 cycles with primer oJBL432, read2 (barcode reverse): 20 cycles with primer oJBL433, index2 (UMI): 10 cycles with primer oJBL434.

3.4 Data pre-processing and quantification

Sequencing data was demultiplexed using bcl2fastq. Raw fastq files were processed first by trimming unnecessary cycles from the 3’ end (9 cycles from read 1, 4 cycles from read 2) using seqtk (https://github.com/lh3/seqtk). Forward and reverse barcode reads were joined and error corrected with PEAR[95] (options -v 16 -m 16 -n 16 -t 16). Using custom python and R scripts, successfully assembled barcode reads were combined with UMI reads, barcode/UMI pairs were counted, and the read counts and UMI count per barcode was determined. These barcode count files served as the processed inputs for downstream analysis.

For downstream processing, the identity of barcodes present in each transfected plasmid library (p053 and p054) was first determined by inspecting the distribution of barcode UMI counts from separate libraries directly prepared from the respective plasmids. The count distribution displayed clear bimodal nature, and barcodes in the high count mode (>9 UMIs for p053, >5 UMIs for p054) were retained as valid. Following removal of barcodes present in both libraries (34 out of 193705), we were left with a list of barcodes for expression analysis (59.0k for p053, 134.6k for p054).

For quantifying steady-state expression of linear and circular barcodes, we tallied the UMI counts for all valid barcodes for genomic DNA and RNA derived libraries. DNA UMI counts were reasonably correlated from genomic DNA to plasmid (R^2 of log-transformed BC UMI counts= 0.42, and 0.43 respectively for replicate 1 and 2). Steady-state expression (referred to as “activity”) was defined as the normalised RNA UMI counts over the normalised DNA UMI counts, with normalised UMI counts

defined as UMI counts over all UMIs mapping to valid barcodes in the respective libraries. For BC well represented in the library (>50 DNA UMI counts), the activity was >150-fold higher for Tornado barcodes compared to linear barcodes (**Supp. Fig. 2e**, median activity fold-change 162× in replicate 1 and 186× in replicate 2). Difference in activity was largely insensitive to threshold selection on DNA UMI, and the summed RNA/DNA UMI counts across all linear vs. circular barcodes irrespective of DNA UMI counts confirmed >100-fold higher in activity for circular over linear barcodes (107× for rep1, 113× for rep2). Circular barcodes had a tight range in activity across barcode sequences (interquartile range in activity spanning 2.5-fold and 2.6-fold for replicates 1 and 2 respectively).

3.5 Estimating expression levels of oBC per cell per integrated cassette

To estimate the relative steady-state expression level of oBC driven by human U6 Pol III promoters, we used two different reverse-transcription qPCR quantifications, from K562 cells with genome-integrated dual reporter constructs. First, following cell harvesting and RNA extraction as previously described, 1 µg of DNase-treated RNA was combined with 100 pmole of random hexamer in 2 µL of 10 mM Tris 7 buffer, incubated at 65C for 5 min, and placed back on ice. 8 µL of MuLV mix (1 µL 10× buffer, 0.5 µL 10 mM dNTP mix, 6 µL DEPC treated water, 0.5 µL MuLV [NEB]) was added to the RNA and random hexamer mix, and incubated at 25C for 5 min, 42C for 60 min, and 65C for 20 min. RNA was hydrolyzed from the reverse transcription mix by adding 2 µL of 1M NaOH and heating to 95C for 5 min. The cDNA was subsequently neutralised by adding 2 µL of 1M HCl, and diluted ten-fold by adding 86 µL of 10 mM Tris 8. For each primer pair, 2 µL of diluted cDNA was directly used for qPCR, and run by adding 2 µL of 10 mM Tris 8 and 5 µL of PowerUp master mix (ThermoFisher) and 1 µL forward+reverse 5 µM primer mix per well. Each primer pair/sample was run in technical triplicate wells, with PCR conditions (2 min at 50C, 2 min at 95C, and cycles: 15s at 95C, 15s at 60C,, 15s at 72C). qPCR primers targeting both the reporters (Pol III oBC: oJBL246+oJBL247, Pol II GFP mRNA: oJBL039+oJBL040), and highly expressed endogenous genes EEF1A1 (oJBL001+oJBL002, with these primers obtained from [132]) were used. oBC expression was normalised to EEF1A1 level using a ΔCt method. To normalise for multiplicity of integration in the genome, we performed qPCR from extracted genomic DNA extracted with DNeasy (Qiagen), using 100 ng gDNA input per triplicate (5 µL PowerUp SYBr mix, 1 µL forward+reverse 5 µM primer mix, and 10 mM Tris 8 to 10 µL), using the same cycling parameters as for RT-qPCR, and primers targeting the piggyBac reporter payload (GFP: oJBL039+oJBL040, puromycin cassette: oJBL043+oJBL044) in addition to endogenous genes for normalization (RPPH1: oJBL085+oJBL086, TERT: oJBL091+oJBL092). Relative levels of oBC RNA and Pol II GFP mRNA compared to the endogenous EEF1A1 mRNA were normalised by DNA dose per cell inferred from qPCR, leading to 5.2±0.8 for oBC and 0.12±0.04 for GFP (± standard error of the mean from 4 biological replicates), as the estimated expression per integrated copy expression. GFP level was the average produced by the five promoters included in the exogenous library (no promoter, minimal promoter, UBCp, Pgk1p, EEF1A1p), most of the expression coming from EEF1A1 promoter, and indeed close to the expected level of the endogenous EEF1A1 mRNA level when correcting for this factor (5×0.12=0.6≈1).

As an additional measurement, which might be not affected by possible systematic underestimation given the fact that oBCs are short (134 bp), leaving fewer space for priming from random hexamers, we used the qPCR cycle number obtained from preparation of sequencing libraries, which involves reverse transcription from target specific primers instead of random hexamers. For oBC, the same approach described for the linear vs. circular barcode MPRA was taken. For the mBC (the Pol II reporter mRNA), we used the same procedure, except with the following primers: reverse

transcription with primer oJBL358, PCR1 with primers oJBL077+oJBL039, PCR2 with primers oJBL077 and one of oJBL359-oJBL366. Comparing the qPCR Ct value for oBC vs. mBC libraries, we estimated a ΔCt of 10.4±1.2 (± spread across two biological replicates) corresponding to a relative abundance fold-change of ≈1300 for oBC vs. mBC (we note that different RT or PCR primer efficiency could drive part of this difference), which is internally controlled for multiplicity of integration as both are part of the reporter construct. Correcting for the difference between the endogenous EEF1A1 mRNA and GFP reporter (per integrated copy) seen with random hexamers led to an estimate of 1300×0.12=156-fold higher oBC expression compared to the EEF1A1 mRNA, which is one of the most highly expressed mRNA in K562 cells (as assessed from the average of stranded bulk RNA-seq datasets from ENCODE [97] in K562). Given the presence of multiple rolling-circle reverse transcription products (**Ext. Data Fig. 1j**), we note that this quantification can be considered a slight overestimate. Taking the geometric mean of the random hexamer and target specific primer as an estimate of oBC abundance leads to ≈32-fold higher expression of oBC relative to the EEF1A1 mRNA. Given that EEF1A1 comprises 1.2% of mRNAs in K562 (estimated from bulk RNA-seq TPM), and taking 200,000 total mRNAs per cell (BNID109916 [133]), this leads to an estimate of 1.2%×200,000×32 >75,000 oBC RNAs per cell per integrated copy of the cassette in the genome, which converted to concentration assuming a radius of 10 um for K562 cells, leads to ≈30 μM.

4 Assessing the influence of cHS4 and U6/oBC on genomic integration positional effects

4.1 Experiment description and statistical rationale

We sought to quantify whether the Pol III promoter cassette (U6 promoter driven Tornado barcode) affected the sensitivity of the Pol II expression to genomic integration positions in our random transgenesis approach, especially in comparison to a canonical insulator element (cHS4, present in our original reporter architecture). To do so, we constructed a series of four different piggyBac transposon backbone with the four possible combinations of +/- insulators and +/- U6/oBC. The five exogenous promoters (**Fig. 2a**) were then cloned to drive the expression of a barcoded mRNA within the context of all four different reporter architectures (**Supp. Fig. 1a**).

We reasoned that starting from a complex library of redundantly barcoded reporters and following integration of the library in a polyclonal population, bottlenecking of the population such that the number of total integrations ($N_{integrations}$:= number of clones × average number of integration per clones) was much smaller than the total number of barcodes in the starting plasmid library (defined as N_{mBC}) would ensure that each barcode would be associated to a probabilistically unique genomic integration position. Measuring the expression level of these reporters using conventional bulk MPRA and assessing variability in the expression across barcodes would then provide a measure of variation coming from positional effects for the different reporter architecture.

Statistically, the likelihood of having a barcode integrated at multiple positions can be estimated. Assuming equal representation of the barcodes in the library (best case scenario), the distribution of number of integration in the population of any specific barcode follows a Bernoulli process with trial number equal to the total number of integrations $N_{integrations}$, and success probability equal to $1/N_{mBC}$. The probability to have more than one integration for any specific barcode is then:

$$1 - \left(1 - \frac{1}{N_{mBC}}\right)^{N_{integrations}} - \frac{N_{integrations}}{N_{mBC}}\left(1 - \frac{1}{N_{mBC}}\right)^{N_{integrations}-1} \approx \frac{1}{2}\left(\frac{N_{integrations}}{N_{mBC}}\right)^{2} \; if \; N_{integrations} \ll N_{mBC}$$

Hence, if the sampled barcode complexity in a bottlenecked population is much less than that of the full library, each detected barcode will indeed be probabilistically uniquely integrated. In the context of bulk

MPRA (without positional mapping), the number of independent integrations is not directly measurable. Instead, what is observable is the number of detected barcodes from the library, which equals the number of different unique barcodes sampled (above a representation threshold). In the above approximate Bernoulli process, the correction is proportional to the fraction of barcodes sampled twice or more (above), and is small in the considered limit, and the proportion of detected barcodes is then to leading order $N_{integrations} (N_{mBC})^{-1}$. The fraction of detected barcodes that are not uniquely integrated can then be estimated as the proportion of barcodes with more than one integration (above) over the proportion of detected barcodes, or approximately $N_{integrations} (2\ N_{mBC})^{-1}$.

4.2 Construction of reporter libraries with/without cHS4 and U6/oBC

4.2.1 Cloning of reporters

The 20 promoter libraries (five promoters × four reporter architectures) were cloned separately and pooled prior to transfection. Briefly, in order to construct the libraries, four high complexity (large number of mBCs in library) barcoded backbones were used as starting points: p22 (with insulators, without U6/oBC), p25 (with insulators, with U6/oBC), p93 (without insulators, without U6/oBC), and p94 (without insulators, with U6/oBC) (**Supp. Fig. 1a**). The re-cloned high complexity p25 backbone used to construct scQer libraries was used directly for this purpose. A high-complexity p22 library was generated by a three fragment Gibson assembly, each obtained from PCR of the original p22 (backbone fragment1 with primers oJBL524+oJBL527; backbone fragment2 with primers oJBL526+oJBL528; barcoded insert fragments obtained with two rounds of PCR with primers oJBL514+oJBL518 followed by oJBL516+oJBL518; all fragments were size selected on agarose for backbone fragments and PAGE for barcoded insert), and electroporated in in 25 uL of C3020 cells (NEB). p93 and p94 were obtained by Esp3I digest of the insulator free piggyBac transposon plasmid pXL005, followed by addition of barcoded inserts with Gibson assembly (obtained from PCR with primers oJBL679+oJBL680 respectively from p22 and p25) and electroporation in 25 uL of C3020 cells (NEB). All backbone were at high complexity (>1M transformants as estimated by plating a) and were confirmed by Sanger sequencing. We note that the barcode sets of p22/p93 and p25/p94 overlap as a result of the cloning strategy, but that given bottlenecking of the final libraries, the number of colliding barcodes was minimal.

From these barcoded backbones, ectopic promoters were introduced (separately for each backbone/promoter pair) as described before, with BglII+EcoRI digest and Gibson with PCR products containing the different promoters with homologies to the backbone (see section 1.1 for details). The resulting plasmids were transformed individually in 25 uL of C3040 cells (NEB), *de facto* leading to a bottleneck (estimated complexity of transformant ~500 to 20k depending on the library). Cells were outgrown overnight and plasmids purified by midiprep (Zymo).

4.2.2 Association of mBC to promoters

To obtain the list of mBC corresponding to each plasmid library, we prepared amplicons for sequencing similarly to the DNA arm of bulk MPRA, as described before. Starting from 5 ng of each plasmid library, two rounds of PCR were performed (4 cycles PCR1 with primers oJBL039+oJBL753 to append a pseudo 10 bp UMI, followed by 8 cycles with oJBL361 and Nextera v2 P7 indexed primers). Amplicons were cleaned up with Ampure XP beads (1x), and pooled for sequencing. Sequencing was performed on a Nextseq2000 using custom set of primers (read 1: oJBL369, 15 cycles to read mBC; index 1: oJBL335 [Nextera index 1], 10 cycles to read the sample index; read 2: oJBL494 [Nextera read

2], 10 cycles to read the pseudo-UMI; index 2: oJBL371, 15 cycles to read the reverse complement of the mBC). The data was processed to a piled up file (counting number of reads and UMI per barcode per library) as described before. The count distributions per barcode per library were inspected and found to be bimodal. The *bona fide* barcodes present in the libraries were taken to be those in the high count mode (count threshold the minimum of the bimodal distribution), leading to 153.9k barcodes across the 20 libraries. To ensure no inter-library barcode collision (given that the libraries were pooled for the experiment), the final list of barcodes used was filtered to only have barcodes present in a single library out of the 20 pooled for the experiment, leading to a final set of 134.1k barcodes (13% multiply represented barcodes removed). Barcode complexity per library spanned 588 to 22.1k with interquartile range 3.6k to 11.3k.

4.3 Transfection and bottlenecking of cell population

Libraries were pooled in accordance with the estimated number of barcodes per construct to ensure similar representation of individual barcodes (i.e., pooled not equimolar per library, but equimolar per barcode). This was to ensure that barcodes of the less complex libraries remained predominantly uniquely integrated. Pooled libraries were transfected in K562 and HEK293 (6.5 M K562 cells with nucleofector, kit V4XC-2024 (Lonza BioResearch) program FF120, with 8 ug libraries pool [transposon] and 400 ng hyPBase transposase; 2M HEK293 cells, lipofectamine2000 with 8 ug libraries pool [transposon] and 400 ng hyPBase transposase). Cells were allowed to recover for five days and passaged upon confluence (HEK293) or reaching 1 M/mL (K562). Following five days, 2 ug/mL puromycin selection was applied for another 7 days to select for piggyBac-mediated genomic integration of reporter transposons (which contained the GFP-P2A-puromycin resistance ORF). After dilution of the unintegrated plasmids (12 days post transfection), polyclonal cell populations were seeded at an estimated starting number of clones of 3k, 10k, and 30k each in biological duplicates. After expansion of populations to approximately 5M cells (7 to 9 days), cells were harvested, fixed in 80% methanol, and stored at -80C until extraction.

4.4 Bulk MPRA experiment and data analysis

4.4.1 Bulk MPRA library construction

RNA and DNA was extracted as before from methanol fixed cells (AllPrep kit, Qiagen). MPRA libraries were constructed as previously described, with slight modifications. Briefly, for RNA libraries, 5 ug of RNA was reverse transcribed with UMI containing primer oJBL753 using SuperScript IV (10 uL 500 ng/uL RNA mixed with 2 uL 1 uM oJBL753; 5 min at 65C, ice for >2 min; followed by addition of 4 uL 5x buffer, 1 uL 0.1M DTT, 1 uL 10 mM dNTPs, 1 uL water, 1 uL SSIV as a master mix for 20 uL total reaction; 55C for 60 min, 80C for 10 min). The cDNA was taken directly as template for PCR1 (25 uL reactions, 10 uL cDNA, primers oJBL039+Nextera v2 P7 indexed primers; 4 elongation cycles). Following the first PCR, reactions were cleaned up with 1x Ampure XP beads, eluted in 12 uL 10 mM Tris 8. 4 uL of PCR1 eluates were taken to PCR2 (10 uL reactions, primers oJBL077+oJBL359, tracked with qPCR and SYBr green, stopped at cycles 9-15 depending on reaction's inflection point). Reactions were again purified with 1x Ampure XP and eluted in a final volume of 10 uL 10 mM Tris 8. For DNA, approximately 10 ug genomic DNA was amplified in 25 uL reactions (primers oJBL039+oJBL753; 4 cycles). Following 1x Ampure XP cleanup and elution in 12 uL 10 mM Tris 8, 4 uL of the eluate was amplified in a second round of PCR (10 uL reactions, 15 elongation cycles; primers oJBL360+Nextera

v2 P7 indexed primers), cleaned up with 1x Ampure XP, and eluted in 10 uL 10 mM Tris 8. Each biological replicate was process in technical duplicate, for a total of 48 libraries (2 cell lines × 2 library types RNA/DNA × 3 bottlenecking factors × 2 biological replicates × 2 technical replicates). All reactions were performed in 96-well plates and processed at the same time as the bulk MPRA experiment from EBs to assess different aspects of reporter architecture (see section 5). To quantify final amplicon concentrations, a real-time qPCR experiment was performed using P5 and P7 primers (oJBL076, oJBL077 respectively). Samples were pooled according to their concentration as determined by qPCR and sequenced on a Nextseq2000 using custom set of primers (read 1: oJBL369, 15 cycles to read mBC; index 1: oJBL335 [Nextera index 1], 10 cycles to read the sample index; read 2: oJBL494 [Nextera read 2], 10 cycles to read the pseudo-UMI; index 2: oJBL371, 15 cycles to read the reverse complement of the mBC).

4.4.2 Bulk MPRA analysis

Bulk MPRA data was processed as previously described. Libraries were sequenced at an average 2M reads/library (interquartile range 1.5M to 2.75M). A threshold of 10 DNA UMI per barcode was selected to deem a barcode as present in the cell populations. For the majority of conditions, the set of barcodes detected in cells were indeed only a fraction of those present in the libraries (median recovery across 20 libraries: HEK293_03k: 6.0%, HEK293_10k: 10.4, HEK293_30k: 17.8%, K562_03k: 13.1%, K562_10k: 24.8%, K562_30k: 31.0%), as necessary for statistical assumptions of probabilistically unique integrations (see section above). We confirmed that the variability measure was stable over the different populations (except for Pgkp promoters in K562, which showed evidence of lower variability at higher starting population). Within all samples (bottlenecked populations), for each barcode detected (DNA UMI>10), the activity was computed as the normalised RNA UMI (RNA UMI count over the summed RNA UMI count for the sample) over the normalised DNA UMI. Example activity score distributions from active promoters for replicate HEK293 bottlenecked at 30k (rep1.1) are shown in **Supp. Fig. 1b**. Variability across activity scores measured from the mBCs is quantified as the ratio between the 75th and 25th percentile (shown for all replicates in **Supp. Fig. 1c** for HEK293, and **Supp. Fig. 1d** for K562). A two-sided Wilcoxon test (Bonferonni correction) was used to compare this metric of variability between the following pairs of reporter architecture for all active promoters (UBCp, Pgk1p, EEF1A1p): (U6/oBC+, cHS4+) vs. (U6/oBC-, cHS4+), (U6/oBC+, cHS4+) vs. (U6/oBC+, cHS4-), (U6/oBC-, cHS4+) vs. (U6/oBC-, cHS4-), and (U6/oBC+, cHS4-) vs. (U6/oBC-, cHS4-) (**Supp. Fig. 1c**).

5 Control experiments assessing impact of reporter architecture (bulk MPRA in mEBs)

In order to test the influence of various components of the scQer architecture, we constructed a series of libraries harbouring different components/positions of the regulatory elements (summarised in **Supp. Fig. 8a**). We then directly compared the activity of the CREs within the different reporter architecture using bulk MPRA across mouse embryoid body differentiation series.

5.1 Construction of reporter libraries with different architectures

5.1.1 Cloning of reporter libraries

Given that some reporter architectures lacked oBC (to explicitly test the influence of the Pol III cassette), which we had previously used for barcode dictionary generation, we had to adapt our cloning

strategy to be able to directly subassemble the CREs to the mBC. We describe in turn the different approaches taken.

Libraries p058 & p059 (with vs. without oBC): to clone the standard scQer cassette with and without the Pol III cassette, we started from the high complexity barcoded plasmid docks p022 (no oBC, mBC only) and p025 (with both oBC and mBC). In contrast to the early strategy (highlighted in **Supp. Fig. 2**), we first inserted the library of CREs (same inner PCR pool as previously) using Gibson assembly from the NheI and MfeI digested barcoded backbone, leading to intermediate plasmids p056 (no oBC) and p057 (with oBC) respectively. These libraries were bottlenecked to 1% post transformation (electroporation) to be in the appropriate complexity range (~100 mBCs per CRE). p056 and p057 served as template for subassembly (see next section for details). To complete the library, we then integrated the minP-GFP cassette. As another difference from the previous approach (linearization of backbone with EcoRI+BglII), to avoid losing a substantial fraction of our CRE due to these frequent cutters, we used spCas9 (NEB) to perform *in vitro* digestion at a specific site on the plasmid using a specifically designed crRNA (roJBL677). Following duplex formation with the tracrRNA (Alt-R® CRISPR-Cas9 tracrRNA, IDT) (5 uL 100 uM of each crRNA and tracrRNA, 95C for 5 min, then cool to room temperature), 2 ug of p056 and p057 plasmids were digested (150 uL reactions: 15 uL r3.1 buffer, 5 uL spCas9, 15 uL 300 nM crRNA:tracrRNA duplex, 100 uL nuclease free water, incubate 10 min at 25C, addition of plasmid, incubate 15 min at 37C, addition of 5 uL 100 mg/uL ProK, incubate 10 min at room temperature, 0.5x Ampure XP clean up and elution in 20 uL prior to run on gel) and size selected on agarose gel. The minP-GFP insert with compatible homologies for Gibson assembly was obtained from PCR with primers oJBL254+oJBL676, and assembled with the p056 and p057 libraries (serving as template for CRE-mBC subassembly, see below), and electroporated in a high efficiency transformation to maintain complexity. The resulting libraries, p058 (no oBC) and p059 (with oBC), were purified (midiprep, Zymo) and mixed in as parts of the final transfected pools of the bulk MPRA experiments. We note that library p059 effectively consisted in a very similar construct compared to the original p055 (only difference: a short 41 bp added sequence [containing the original BglII+EcoRI insertion site] between minP and the CRE due the different integration strategy for p058), and was used to assess internally the reproducibility of our bulk MPRA measurements.

Library p092 (no minP): to construct scQer reporters without the minimal promoter, we first inserted a no promoter GFP cassette into high oBC-mBC complexity library p025 (Gibson assembly, backbone: BglII+EcoRI selected on agarose, insert: noP-GFP constructed by fusion PCR [primers oJBL254+oJBL314, final product size selected on agarose] with two shorter fragments, themselves obtained by amplifying p029 with primers oJBL314+oJBL417, oJBL254+oJBL414). The resulting library (obtained from high efficiency electroporation), p045, was then digested with MfeI and NheI (NEB) and the CRE pool (same inner PCR pool as before) inserted by Gibson assembly, electroporated (no bottleneck was implemented, instead diluting the electro-competent cells in 10% glycerol 10-fold prior to electroporation). The resulting plasmid library p092, was used for subassembly between CRE and mBC (via the oBC as before), and for the final pool transfected into cells.

Libraries p091 and p096 (CRE downstream, without and with oBC): to create constructs in which the CREs were positioned downstream of the reporter cassette and promoter, we first introduced the minP-GFP fragment in high complexity plasmids p022 and p025 as before (Gibson; backbone: BglII+EcoRI digest with agarose size selection, insert minP-GFP as previously; electroporation). The resulting high complexity libraries, respectively p090 and p095, were then digested using new sites which by happenstance happened to be paired unique cutters between the SV40 poly-A signal and the downstream cHS4 insulator: EcoNI+BtsXI (NEB). To append compatible homology handles to this new

portion of the backbone, the inner PCR pool of CREs was re-amplified in a low-cycle PCR reaction with primers oJBL674+oJBL675, and following 1x Ampure XP clean up, inserted by Gibson assembly. The resulting high complexity libraries, p091 (no oBC, CRE downstream) and p096 (with oBC, CRE downstream), were each bottlenecked to an estimated 50k clones, and plasmid libraries were purified and served as template for CRE-mBC subassembly (new approach, see below), and used directly for transfection into cells as part of final pools.

Cloning of the promoter series without oBC libraries (p033, p034, p035, p039, p040: exogenous promoters without oBC cassette) proceeded exactly as described in a previous section (different pool).

5.1.2 mBC to CRE subassembly strategies

As alluded to above, we took different strategies for the different reporter architectures to map mBC to CREs. We describe below the various strategies used for the different cassettes.

Subassembly of libraries p058 & p059: the connection between mBC and CRE was obtained using a similar strategy as that connecting oBC to CREs. Briefly, the plasmid library was tagmented as described before (section 2.4.4). Instead of using primers upstream of the oBC, primers downstream of the mBC were used. Following 13 cycles of semi-specific PCR (primers: indexed Nextera P5 + oJBL358). Fragments in the range 350-700 bp were size selected on PAGE, and paired-end sequenced on a Nextseq500 (read1 32 cycles Nextera_read1 primer: tagmented CRE; read 2 32 cycles primer oJBL371: mBC; index 2 10 cycles Nextera_index2 primer: sample index). Bioinformatic processing of the data was similar as for oBC-CRE subassembly, yielding 15.5k mBC with median 65 mBC/CRE for p058 and 27.8k mBCs with median 117 mBC/CRE for p059 passing the individual library controls (see below for additional filter to avoid inter-library collisions in the pool).

Subassembly of library p092: given the architecture of the reporter was similar to the original p055 (except without the minimal promoter), we used the same strategy (oBC-CRE) as previously described obtaining 49.4k valid mBCs (via the original oBC-mBC pairing) and a median of 207 mBCs/CRE.

Subassembly of libraries p091 & p096: since the CREs were inserted downstream of the GFP reporter, the CREs could be directly subassembled to the mBCs in this context. To do so, we again used tagmentation followed by 13 cycles of semi-specific PCR (primers: indexed Nextera P7 + oJBL708) and a PAGE size selection (600 bp to 900 bp). The library was paired-end sequenced on a Nextseq500 (read1 32 cycles oJBL707 primer: start of inserted CRE; index1 18 cycles Nextera_index1 primer: sample index, read 2 32 cycles primer oJBL371: mBC). Data processing for subassembly was similar as for the oBC-CRE mapping in p055, and leading to identification of 32.0k and 47.3k valid mBCs, and a median of 162 and 241 mBCs/CRE for libraries pJBL091 and pJBL096 respectively.

Subassembly of promoters without oBC (p033 series) was performed by obtaining the list of mBC (PCR amplification and sequencing of the product) from sequencing of PCR products (two steps: PCR1 with Kapa HiFi in 20 uL, 4 cycles, primers oJBL039+oJBL358, Ampure 1x cleanup, PCR2 with Kapa HiFi in 20 uL, primers oJBL077+oJBL362-o366 indexed series for 10 cycles). The product was sequenced as a spike-in on Nextseq 2000 with custom primers (read 1: 148 cycles, primer oJBL369; index 1: 10 cycles, empty [blank read]; index2: 10 cycles, primer oJBL370). Read 1 was sufficiently long to cover both the mBC and the pseudo-UMI installed by PCR1 of the library preparation. Read1 fastq was then trimmed to separate files for the two pieces (mBC and UMI) of information for downstream processing. mBC and UMI where the piled-up as for MPRA amplicons. The bona fide set of mBC present in each respective libraries was then determined from the high-count mode of the bimodal

distribution of UMI count per mBC, yielding a median of 2.8k mBCs per promoter (span of 0.3k to 4.3k mBC for the different promoter, with UBCp being less well represented).

Given that all the libraries cloned were generated as bottlenecked versions of starting high complexity plasmids (p022 and p025), mBCs shared between the final pooled libraries were expected to (rarely) occur. In order to obtain the final list of unique and valid CRE-mBC pairs, we therefore excluded from the final table, any mBC that was present across multiple libraries pooled for the experiment (see next section), as these would have been impossible to interpret (if associated with multiple CREs).

We provide here additional information on quality control checks that were performed as part of these subassemblies. First, oBC-CRE subassemblies were performed on libraries p057 and p059 (only difference being the addition of minP-GFP between the CRE and mBC in going from p057 and p059), to assess possible loss in library complexity in the cloning step. We found an identified oBC overlap of >98% between the two libraries, and an R^2 of the log-transformed oBC counts of 0.91, both arguing in limited loss in complexity in the step of adding the reporter in the library backbone. Second, as a way to test the faithfulness of our triplet dictionary, we performed oBC-CRE and CRE-mBC subassembly on library p057 (feasible because the reporter is not yet integrated as described above). We found 99.5% concordant associated CREs (fraction of agreement in associated CREs between detected predetermined valid oBC-mBC pairs).

5.2 Transfection of final pooled libraries and mEB induction

Two pools of libraries were generated for the purpose of experiments, with the following final compositions (by mass). Pool A: 30% p058, 17.5% p059, 30% p092, 17.5% p055 (original library from the first round of experiments), 2.5% p27 series (original exogenous promoter pool), 2.5% p33 series (exogenous promoters without oBC cassette, itself equal mass pool of p033, p034, p035, p039, p040). Pool B: 37.5% p091, 37.5% p096, 20% p055 (original), 2.5% p27 series (original), 2.5% p33 series.

The two respective pools were transfected (lipofectamine 2000) in mESCs as previously described, except each reaction scaled up by 2-fold. Specifically, each pool was transfected in 6 separate reactions (2M cells transfected and plated in 6 cm plates) with 400 ng hyPBase plasmid, 8 ug of reporter pool. Following recovery of cells for 2 days, cells from pairs of transfection replicates from each pool were combined (from 6 to 3 plates of cells), and the resulting three set of cells were hereafter maintained separately, constituting our biological triplicates. Puromycin (2 ug/mL) selective pressure was applied 3 days post transfection and until induction of mEBs, which happened 11 days post transfection. mEB induction proceeded as previously described, with 15M of cells split in 5 plates (3M/plate) for each biological replicate. 3M of cells per replicate were also sampled for the day 0 time point. Plates from the same replicates were mixed at each medium change (once every two days). One plate's worth of mEB was harvested on days 4, 18, 20, and 22. At harvest, cells/mEBs were fixed in 80% methanol and placed at -80C until RNA/DNA extraction for library preparation.

5.3 Bulk MPRA experiment and analysis

RNA/DNA extraction, MPRA library preparation (with the updated amplicon design performed in technical duplicate for each sample), sequencing, preprocessing and aggregation to count tables proceeded as described in section 4.4, with the only modification that the threshold for including mBCs in the derived MPRA activity was taken to be >3 DNA reads per barcode. The per-CRE activity derived

from MPRA counts was taken as the summed normalised RNA UMI over summed normalised DNA UMI from all mBCs corresponding to a CRE (from the subassembly). The activity score for each CRE was calculated separately for the different libraries (reporter architectures) for each sample (corresponding to sample time, biological/technical replicate). The displayed scores (**Supp. Fig. 8b-c**, **f**, and **h**) corresponded to the median across replicates, and the error bars mark the interquartile range (25th to 75th percentiles).

6 Singleton validation experiments

In order to obtain orthogonal (not relying on single-cell genomics) evidence of the autonomous & cell-type-specific activity of the CREs identified in our initial screen (experiment of **Fig. 3-4**), we cloned active elements individually. These constructs were then respectively transfected and genome-integrated in separate cultures of mESCs, following which each culture was differentiated to embryoid bodies (as described above) over >3 weeks. Epifluorescence pictures were taken throughout the time course and with structured illumination (end point) to assess the resulting domains of reporter expression within mEBs.

6.1 Cloning of singleton mCherry scQer reporters

To allow for co-transfection and selection on puromycin with the promoter series (reporter ORF: puromycin-P2A-GFP), we constructed a scQer reporter with mCherry instead of GFP. Briefly, barcoded backbone p025 was digested with EcoRI and BglII and size selected on agarose. The minP-mCherry cassette was generated by splice PCR from two PAGE size selected PCR fragments (fragment minP: primers oJBL314+oJBL416, template p027; fragment mCherry: primers oJBL254+oJBL414, template p060) using primers oJBL254+oJBL314. The resulting minP-mCherry insert was size selected on PAGE, inserted by Gibson assembly into the digested p025 backbone to generate plasmid p062, and the library electroporated in *E. coli* as before (NEB, C3020). Plasmid library p062 was digested with MfeI and NheI and size selected on agarose. The resulting linear backbone was compatible with the inner PCR products used to clone the initial library of CREs (see section 2.4.3), and the products for the 8 most specific CREs were separately inserted by Gibson assembly to generate 8 distinct plasmid libraries (p065 *Lama1*:chr17_7784, p066 *Lamb1*:chr12_2183, p067 *Foxa2*:chr2_13858, p068 *Gata4*:chr14_5729, p069 *Sox2*:chr3_2007, p070 *Sox2*:chr3_2009, p071 *Bend5*:chr4_8201, p072 *Epas1*:chr17_10063). Assembled plasmids were transformed into *E. coli* (NEB, C3040). The singleton plasmids were separately purified for transfection into cells. The number of unique barcode pairs per construct was estimated to be around 100-400 (by counting plated colonies). In this case, we however did not sub-assemble the barcode dictionary as a sequencing-based readout was not used for these experiment. All plasmids were verified by Sanger sequencing.

6.2 Singleton mEB differentiation experiment

We transfected the plasmids in mESC grown as before with lipofectamine 2000 individually in separate cultures (0.5 M cells in 1 well of 12-well plate; 1 ug scQer mCherry singleton plasmid, 100 ng promoter puromycin-GFP series plasmid pool [from **Fig. 1**], 100 ng hyPBase plasmid). Following cell recovery, puromycin selection (2 ug/mL) was applied (day 3) until EB induction (day 10). On EB induction day, cells from the single-cell suspension were profiled by FACS to quantitatively measure mCherry expression in the pluripotent state (**Supp. Fig. 4b**). Two plates of mEBs (3 M/plate) per singleton construct were initiated as described before. On every medium change (every two days), epifluorescence images were taken (examples in **Supp. Fig. 4c**) from each culture to assess mCherry expression. While the *Sox2* elements both showed high activity at day 0, consistent with their activity in the pluripotent state, both in FACS and from epifluorescence (**Supp. Fig. 4b-c**), mEBs harbouring parietal endoderm elements initially displayed essentially no expression above background, with a fraction of them (for all elements) inducing expression over the time course (**Supp. Fig. 4c**). Domains of expression between the Sox2 (pluripotent-specific) elements sharply contrasted (internal, spotted) with

that of parietal-specific elements (all on surface, **Supp. Fig. 4d-m** and **Supp. Fig. 5**). These observed domains of mCherry expression were in line with early observations in embryoid bodies reporting endodermal cells on the surface with a rough morphology[49] indicative of basement membrane component production, and similar to spatial patterns of expression directly observed for *Gata4*[134].

7 Example applications of scQers: CRE pairs and TF binding sites allelic series

To illustrate the usefulness of scQers to study questions in regulatory genomics, we constructed three new libraries (literature-selected, paired CREs, perturbed TFBS). These were then profiled as before using scQers (integration at high MOI with piggyBac in mESC, differentiation to mEBs over 3 weeks and single-cell endpoint profiling).

7.1 Identification of putative transcription factor binding sites

We hypothesised that putative transcription factor binding sites within the identified cell-type-specific CREs would be important sequence features to perturb and lead to large changes in activity.

7.1.1 Putative Transcription factor binding sites identification

To characterise the transcription factor binding composition of tested elements using a biophysically grounded empirical approach (in the absence of high resolution ChIP-seq data in our system), we took an approach inspired by Farley and colleagues[27,115]. Briefly, we obtained protein array binding data from endodermal transcription factors *Gata4*, *Foxa2*, and *Sox17* from Uniprobe[104–106], which provides affinity measures for all DNA 8-mers. We converted the raw measurements ("Median" column in the raw data files) to relative affinities. To do so, we treated the mode of affinities as the experimental noise floor, and computed the relative affinity as: (affinity-baseline)(max(affinity)-baseline). We note that the final list contains a relative affinity for an 8-mer and its reverse complement (as the protein binding arrays hold double stranded DNA). Before computing the maximum affinity, we divided the score of palindromic 8-mers by two, as we found those to be anomalously high. The resulting relative affinity 8-mer table was then used to scan all regulatory elements and genomic regions, yielding a value for each 8-bp stretch (in a strand agnostic manner). We then identified local maxima in the relative affinity trace. For a given relative affinity threshold, the local maxima above threshold were retained. To collapse maxima close to each other, we generated a graph between maxima (one node per maximum) with an adjacency matrix determined by distance (connect maxima less than 3 bp apart). Connected components of the graphs were identified, with one putative TF binding site assigned per connected component (typically a single maximum) at the highest-affinity position. The procedure was applied across a range of affinity thresholds, and both on individual CREs (e.g., **Ext. Data Fig. 10** and **Supp. Fig. 7d**) across full genomic loci (±100 kb from TSS, 500 bp windows with 250 bp sliding step and excluding tested CREs and surrounding 500 bp, **Supp. Fig. 7c**).

7.1.2 TF binding site optimization and disruption

To generate CRE variants to test (**Ext. Data Fig. 10d**), we identified putative binding sites for transcription factors *Gata4* and *Sox17* as described above (relative affinity threshold = 0.3) within 6 parietal endoderm CREs. Then, for each class of perturbation (optimization/disruption) and target TF (*Gata4*, *Sox17*, or both), we cycled through the corresponding putative sites. For disruption, the set of 8-mer within a Hamming distance of 2 of the binding site were identified, and the nearby 8-mer with lowest affinity was selected. For optimization, to comprehensively identify the local maximum in sequence, we searched for the highest affinity 8-mer within Hamming distance of 2 of the seven 8-mers within ±3 bp of the local maximum (buffer distance = -3, -2, -1, 0, 1, 2, 3 bp), replacing the optimal

8-mer in its original position within the sequence. For perturbations to both *Gata4* and *Sox17*, we first mutated the *Sox17* sites, and then the *Gata4* sites. For simplicity, we did not prevent optimization/disruption mutations that would affect the other TF (e.g., not preventing mutations to Gata4 sites overlapping with Sox17 sites and vice versa). An example of the affinity traces for original and mutated variants are shown in **Supp. Fig. 7d**. Variant CRE sequences and information on mutations can be found in **Supp. Data 8**.

7.2 Cloning of scQer libraries

7.2.1 Literature selected elements

In order to provide additional tests for the ability of scQers to detect cell-type-specific activity, we search for additional CREss with evidence for function in pluripotent or differentiated mouse stem cells. We selected two regulatory elements from Buecker et al[64] (*Tbx3* and *Esrrb* pluripotent-specific), two *Nodal* CREs from Papanayotou et al[65], one neural *Sox2* element[67], and one *Cdx2* intronic CRE[66]. These were PCR cloned as previously (unburdened outer PCR followed by inner PCR with cloning handles, oJBL632-641 and oJBL644-657; see **Supp. Data 4** for description of primers and the sequences and genomic coordinates of CREs tested). Cloning handles were compatible for Gibson assembly with p043 (digested with MfeI+NheI), were integrated as a pool as before, electroporated in *E. coli* (NEB, C3020). The resulting library was bottlenecked to ~20k clones, and the plasmid purified for subassembly and transfection in mESCs.

7.2.2 Paired CREs combinatorial assembly

To assemble pairs of CREs on the same construct, we used the common handles (homology arms used to clone in the original barcoded backbone p043) as primer binding sites to append new homology arms compatible with pairwise combinatorial assembly. Briefly, a new barcoded backbone with updated homology arms for integrating CREs was created. p043 was digested with HindIII and NcoI, size selected on agarose and a new replacement insert with updated homology arms, gene block gJBL009, was integrated by Gibson assembly. The resulting library, p063, was electroporated (NEB, C3020) and expanded while maintaining complexity. Plasmid library p063 was the same as p043, but now with new homology arms: upstream 5’ AGGACTCTACCAACGCTAGTCCAAGCAAGG and downstream 5’ gtgcagcgcgatgtatagcagtgcgcgaag. For each of the six selected CREs (pluripotent specific: P1 *Sox2*:chr3_2007, P2 *Sox2*:chr3_2009; parietal endoderm specific: E1 *Epas1*:chr17_10063, E2 *Gata4*:chr14_5729; inactive: I1 *Cdk5r1*:chr11_12590, I2 *Col5a1*:chr2_2586), four PCR products were generated, corresponding to targeted order and orientation in the final assembly (oJBL578+oJBL579 upstream forward Uf, oJBL580+oJBL581 upstream reverse Ur, oJBL584+oJBL585 downstream forward Df, oJBL582+oJBL583 downstream reverse Dr), using the previous inner PCR products as template (all with same common handle). The 8 above primers were designed to either have one homology to the backbone, or one homology to a central joining region (to pair two CREs, central junction sequence: 5’TGACGAAGCTATACTCGGTCGCGAGGACGT), such that the resulting PCR products would be assembled obligately as a pair. The 24 different products were size selected on agarose. Two Gibson assemblies were performed for combinatorial assembly with the MfeI & NheI digested updated backbone p063 described above. With the shorthand notation just introduced, assembly 1 pluripotent upstream/endoderm downstream: [P1_Uf, P1_Ur, P2_Uf, P2_Ur, I1_Uf, I1_Ur] assembled with [E1_Df, E1_Dr, E2_Df, E2_Dr, I2_Df, I2_Dr]. Assembly 2 endoderm

upstream/pluripotent downstream: [E1_Uf, E1_Ur, E2_Uf, E2_Ur, I2_Uf, I2_Ur] assembled with [P1_Df, P1_Dr, P2_Df, P2_Dr, I1_Df, I1_Dr]. All 72 (9 possible CRE pairs × 2 orders × 4 relative orientations) possible combinations of order and orientation of CREs would then be represented across these two pools, for example E1_Uf::P2_Dr, etc. An example annotated plasmid with combination P1_Ur::E1_Dr is included. The two libraries were each bottlenecked to ~20k and ~1k constructs, and purified for subassembly and transfection.

7.2.3 CREs with mutated putative transcription factor binding sites

The mutated CREs with optimised and disrupted putative binding sites to *Gata4* and *Sox17* (see above) were synthesised (eBlocks, IDT) with the same homology handles for Gibson assembly in the barcoded backbone (p043) as the original cloning strategy. The eBlock CREs were pooled at equimolar ratio and inserted in p043 digested with MfeI & NheI as before. The resulting library was electroporated in *E. coli* (NEB, C3020), bottlenecked to about 75k clones, and purified for barcode subassembly and transfection.

<u>7.3 Generation of barcode to CRE dictionaries</u>

Subassembly of the CRE to barcodes for the literature selected elements was performed as before (tagmentation with semi-specific PCR, size selection on PAGE, sequencing of oBC & tagmented CRE on a paired-end Illumina run). On the other hand, given the nature of the libraries, the paired CREs and mutated regulatory elements required slightly different approaches, as detailed below.

7.3.1 Connecting pairs of CREs to barcodes with long read (Nanopore) data

The minimum distance between the oBC and the second CRE in a construct with two ~1kb size CRE is about 1.4 kb (shorter if the distal CRE is less than 1 kb). Based on quantification of clustering efficiency (~100-fold lower for amplicons of 1.5 kb) on patterned flow cells[135], we attempted to perform the subassembly in the same way as for previous scQer constructs, except size-selecting a larger region on the gel to try to capture the CRE junction and obtain information about the oBC and the two CREs in a single paired-end read (with the oBC read as one of the indexed reads). However, despite selecting for sizes exceeding the first CRE in principle, the recovered reads predominantly were tagmented in the first CRE, underscoring the severe length bias at these amplicon sizes (1-3-1.5 kb) on the short read platforms. While coverage was insufficient to map the oBC-CRE1-CRE2, we obtained enough reads per oBCs in the library to be able to error-correct the slightly error prone and shallowly covered long-read data (see below).

To circumvent the size limitation of the short-read platform, we sequenced the four libraries (pluripotent up::endoderm down high complexity, pluripotent up::endoderm down low complexity, endoderm up::pluripotent down high complexity, endoderm up::pluripotent down low complexity) on a MINion flow cell of the Nanopore GridION platform, using sparse tagmentation to linearize the plasmid libraries prior to adapter ligation (Rapid Sequencing Kit, Nanopore).

The nanopore data was processed with custom scripts with the following set of heuristics. First, constant 30 bp ‘signposts’ sequences flanking variable regions were selected to establish as reference positions within the nanopore read:

CRE upstream: 5’ TGGCGAGGACTCTACCAACGCTAGTCCAAG,
CRE junction: 5’ TGACGAAGCTATACTCGGTCGCGAGGACGT,

CRE downstream: 5’ GTGCAGCGCGATGTATAGCAGTGCGCGAAG,
mBC upstream: 5’ CGAGCTGTACAAGTGAACGCGTTAAGTCGA,
mBC downstream: 5’ TCGACAAGCTCACCTATTAGCGGCTAAGGC.

We then performed local alignments with the Smith-Waterman algorithm (leveraging a fast implementation[136]) using the above signpost sequences as query, and the nanopore reads as targets. We retained reads with at least 4 of 5 signpost alignments with score ≥50 (≥80% match). Further, only reads with sequences with roughly correct sizes were kept (distance [start to start] in read between CRE upstream and CRE junction, and CRE junction and CRE downstream >500 and <1500, and distance between mBC upstream and mBC downstream >42 and <52). The sequences of the regions intervening between signposts (CRE upstream, CRE downstream, mBC) were then extracted from the reads. The extracted CRE sequences were then aligned by Smith-Waterman (again with the fast implementation) to the expected set cloned in the library as query, and the identity (with the relative orientation) of the maximum scoring CRE was stored (alignment score ≥750 [threshold determined from the distribution of scores], otherwise treated as unmapped). Finally a pile-up table counting the number of reads supporting any given combination of oriented CRE upstream/downstream and mBC was compiled and served as starting point for error correction from the Illumina sequencing (this step was useful given that the libraries were not fully saturated from the Nanopore data and each construct not supported by multiple reads, especially for the high complexity libraries).

We leveraged the high coverage of the oBC from our attempt at subassembly from Illumina data to error-correct the Nanopore assembled CREup-CREdown-mBC triplets. Briefly, high read count oBC-CREup pairs (libraries saturated, threshold coverage determined from bimodal distribution of counts to select the high count mode) was determined from the Illumina run, and the associated set of mBCs determined from the pre-determined pairs obtained from the starting oBC-mBC p025 library. Then, for each mBC from the Nanopore pile-up file, the Levenshtein distance to all mBC in the Illumina-identified set was determined, and the minimum distance mBC was stored if unique and within a distance of 2 at most. If the oriented upstream CRE (oBC proximal) identified by the Illumina subassembly matched the oriented upstream CRE from the Nanopore data, the error corrected mBC was considered valid. A Nanopore read count threshold was further applied for the low complexity libraries (>1 read for pluripotent up::endoderm down low complexity, >4 reads for endoderm up::pluripotent down low complexity). Following removal of non-duplicated mBC sets and non-unique oBC-mBC pairs, the oBC-CREup-CREdown-mBC tables were saved for downstream analysis.

7.3.2 CRE with allelic series of perturbed transcription factor binding sites

Associating oBC to CRE was also more challenging for the library of variants because of the similarity between the different sequences, requiring overall better coverage (not only in terms of read counts, but also positionally across the full element) and a refined computational strategy.

Following an analogous approach as before for oBC-CRE association (tagmentation & semi-specific PCR), we however size selected three different ranges on the PAGE gel (360 to 600 bp, 600 to 900 bp, and 900 to 1.4 kb). Following separate purifications, the different size ranges were then re-pooled prior to sequencing with a preference towards the longer products (ratio 1:3:9 for short:mid:long) to mitigate the size bias for bridge amplification on the Illumina flow cell and therefore get more uniform coverage of tagmentation event along the variant CREs, helping with sequencing single-nucleotide variants introduced by design to optimise/disrupt putative TF binding sites.

Following sequencing, we applied the following computational strategy to map oBC to variant CRE. First, following alignment (to the set of unperturbed WT CRE as query) of the tagmentation-based read (in CRE), a pile-up table compiling for each pair of detected oBC and associated WT CRE (prior to

variant call) the read count and set of all identified mutations within the reads (with associated read counts per mutation). Only oBC-(WT CRE) with sufficient coverage (empirically set to 90 reads) were retained. From a list of mutations per CRE in the allelic series (see **Supp. Data 8**) and the pile-up table, the following metrics were calculated for every oBC-(WT CRE) pair: total read coverage, mean read coverage per in-variant mutation (for each possible variant), total number in-variant mutations covered by reads, total number of out-variant mutations covered by reads. These metrics were finally used to classify each oBC-(WT CRE) pair to its most likely CRE variant as follows. Non perturbed CREs were identified as sequences with low normalised read coverage per mutation (maximum [across all possible variants] mean in-variant mutation coverage divided by total read counts), with threshold set by the bimodality of the distribution. In cases where the maximum [across variant types] total in-variant mutation read counts was <1.5-fold the second maximum, the variant type with highest per-mutation mean coverage was maintained. In cases where the maximum was >1.5 the second maximum, the corresponding maximum variant was retained. We note that these criteria were selected on the basis of certain mutation sets per CRE being strict subset of larger sets (e.g., *Sox17*-high ⊂ *Gata4*-*Sox17*-high), further complicating the variant call. oBC-(WT CRE) pairs with more out-variant mutations than in-variant mutations were excluded as too heavily mutated and thus unusable.

7.3.3 Final barcode dictionary for pooled experiment

After identification of barcodes in the separate libraries above, we ensured that there were no collisions between barcodes. More specifically, a table compiling all oBC to CRE dictionaries from the libraries used in the experiment (literature-selected CRE, paired CRE, allelic series of mutated putative transcription binding sites, and exogenous promoter set). Then, barcodes duplicated across the libraries were removed as ambiguous for interpretation of the single-cell data. The final number of unique (unambiguously usable) scQer constructs was 114.3k, with the following construct coverage per library:

Paired CREs: pluripotent up::endoderm down high complexity: 14.8k
Paired CREs: pluripotent up::endoderm down low complexity: 2.0k
Paired CREs: endoderm up::pluripotent down high complexity: 21.1k
Paired CREs: endoderm up::pluripotent down low complexity: 0.3k
Literature-selected CREs: 13.7k
Mutated CREs (optimised/disrupted binding sites): 61.3k
Exogenous promoter series: 1.1k

7.4 scQer experiment, data processing, and analysis

The 7 libraries above were pooled with the following proportions (pluripotent up::endoderm down high 13%, pluripotent up::endoderm down low 13%, endoderm up::pluripotent down high 13%, endoderm up::pluripotent down low 13%, mutated CREs 33%, literature-selected CREs 12%, exogenous promoters 3%) prior to transfection in mESCs (biological triplicates, 2M cells per transfection, lipofectamine 2000). Puromycin selection (2 ug/mL) was applied following cell post-transfection recovery (day 3), and mEB induction (4 plates of mEB per replicate, one set of mEB per transfection biological replicate) initiated on day 10 post-transfection after unintegrated plasmid dilution. On day 23 post mEB induction, 2 plate's worth of mEBs cells were processed and sequencing libraries constructed as described before (dissociation of mEB to single-cell suspension, FACS sorting for individual cells, 10X library prep [3' v3.1 with feature barcoding, one lane per biological replicate], scQer library generation: GEx, mBC, and oBC).

Sequencing data was processed as before (oBC & mBC: cell barcode error correction through cellRanger, extraction of barcodes from bam file, UMI error correction and pile-up; GEx: cellRanger 6.0.1 with mm10-3.0.0 transcriptome, retaining high read count cells [>450 transcriptome UMI, >1% and <12.5% fraction of reads mapping to mitochondrial genes], doublet removal with scrublet [<0.3 doublet score], initial processing and dimensional reduction with Seurat [NormalizeData, normalization.method ='LogNormalize'; FindVariableFeatures, nfeatures=1000; ScaleData; RunPCA; FindNeighbors with 50 top principal components; FindClusters, resolution=0.2; RunUMAP]. Putative doublets were further removed by sub-clustering (processing from full data applied to clusters, sub-clusters identified with FindClusters, resolution=0.5; cells from sub-clusters with median scrublet score ≥0.15 were removed). Cells from clusters making up <1% of all cells were not considered. Cells with outlier transcriptome counts (GEx UMI count > 8000) and MOI (number of detected oBC [>10 UMI/cell] > 110) were also not considered further as possible doublets. After these quality control thresholds were applied, we were left respectively with n=6124, 6442, and 7911 cells across our three biological replicates. Clusters were annotated by inspection of marker genes and comparison with previously systematically integrated data (i.e., **Ext. Data Fig. 4a**).

Bootstrap resampling was used to assess activity of CREs. Specifically, for each cell-type and CRE whose activity was to be tested, the set of normalised mBC UMI counts corresponding to detected reporter events (oBC UMI>10/cell) were collated for tests and controls. Given the lower representation (resulting from smaller MOI & less differentiated cells), the bootstrapping analysis was performed on all replicates pooled. These normalised mBC UMI counts were then sampled with replacement for 10^4 bootstraps (number of sampling per bootstrap equal number of detections). For each bootstrap, the 1% winsorised mean was calculated. The bootstrap p-value was taken as the proportion of bootstraps in which the mean from test ≤ control (to assess higher expression, respectively ≥ to assess lower expression). The bootstrap p-values were adjusted to an FDR by the method of Benjamin-Hochberg.

Supplementary Information References

108. Pollard, K. S., Hubisz, M. J., Rosenbloom, K. R. & Siepel, A. Detection of nonneutral substitution rates on mammalian phylogenies. *Genome Res.* **20**, 110–121 (2010).
109. Trapnell, C. *et al.* The dynamics and regulators of cell fate decisions are revealed by pseudotemporal ordering of single cells. *Nat. Biotechnol.* **32**, 381–386 (2014).
110. Shin, H. Y. *et al.* Hierarchy within the mammary STAT5-driven Wap super-enhancer. *Nat. Genet.* **48**, 904–911 (2016).
111. Ma, S. *et al.* Chromatin Potential Identified by Shared Single-Cell Profiling of RNA and Chromatin. *Cell* **183**, 1103–1116.e20 (2020).
112. Crocker, J. *et al.* Low affinity binding site clusters confer hox specificity and regulatory robustness. *Cell* **160**, 191–203 (2015).
113. Samee, M. A. H. *et al.* Quantitative Measurement and Thermodynamic Modeling of Fused Enhancers Support a Two-Tiered Mechanism for Interpreting Regulatory DNA. *Cell Rep.* **21**, 236–245 (2017).
114. Visel, A. *et al.* Functional autonomy of distant-acting human enhancers. *Genomics* **93**, 509–513 (2009).
115. Lim, F. *et al.* Affinity-optimizing variants within the ZRS enhancer disrupt limb development. *bioRxiv* 2022.05.27.493789 (2022) doi:10.1101/2022.05.27.493789.
116. Artus, J., Piliszek, A. & Hadjantonakis, A.-K. The primitive endoderm lineage of the mouse blastocyst: sequential transcription factor activation and regulation of differentiation by Sox17. *Dev. Biol.* **350**, 393–404 (2011).
117. White, M. A. *et al.* A Simple Grammar Defines Activating and Repressing cis-Regulatory Elements in Photoreceptors. *Cell Rep.* **17**, 1247–1254 (2016).
118. Visel, A., Minovitsky, S., Dubchak, I. & Pennacchio, L. A. VISTA Enhancer Browser--a database of tissue-specific human enhancers. *Nucleic Acids Res.* **35**, D88–92 (2007).
119. Hansen, T. J. & Hodges, E. ATAC-STARR-seq reveals transcription factor-bound activators and silencers across the chromatin accessible human genome. *Genome Res.* **32**, 1529–1541 (2022).
120. Glaser, L. V. *et al.* Assessing genome-wide dynamic changes in enhancer activity during early mESC differentiation by FAIRE-STARR-seq. *Nucleic Acids Res.* **49**, 12178–12195 (2021).
121. Datlinger, P. *et al.* Ultra-high-throughput single-cell RNA sequencing and perturbation screening with combinatorial fluidic indexing. *Nat. Methods* **18**, 635–642 (2021).
122. David, F. N. & Johnson, N. L. The Truncated Poisson. *Biometrics* vol. 8 275 Preprint at https://doi.org/10.2307/3001863 (1952).
123. Simeonov, K. P. *et al.* Single-cell lineage tracing of metastatic cancer reveals selection of hybrid EMT states. *Cancer Cell* **39**, 1150–1162.e9 (2021).
124. Granja, J. M. *et al.* ArchR is a scalable software package for integrative single-cell chromatin accessibility analysis. *Nat. Genet.* **53**, 403–411 (2021).
125. Thibodeau, A. *et al.* AMULET: a novel read count-based method for effective multiplet detection from single nucleus ATAC-seq data. *Genome Biol.* **22**, 252 (2021).
126. Stuart, T. *et al.* Comprehensive Integration of Single-Cell Data. *Cell* **177**, 1888–1902.e21 (2019).
127. Rhodes, K. *et al.* Human embryoid bodies as a novel system for genomic studies of functionally diverse cell types. *Elife* **11**, (2022).
128. Mohammed, H. *et al.* Single-Cell Landscape of Transcriptional Heterogeneity and Cell Fate Decisions during Mouse Early Gastrulation. *Cell Rep.* **20**, 1215–1228 (2017).
129. Korsunsky, I. *et al.* Fast, sensitive and accurate integration of single-cell data with Harmony. *Nat. Methods* **16**, 1289–1296 (2019).
130. Kent, W. J., Zweig, A. S., Barber, G., Hinrichs, A. S. & Karolchik, D. BigWig and BigBed: enabling browsing of large distributed datasets. *Bioinformatics* **26**, 2204–2207 (2010).
131. Garreta, R. & Moncecchi, G. *Learning scikit-learn: Machine Learning in Python*. (Packt Publishing Ltd, 2013).
132. David, F. P. A., Rougemont, J. & Deplancke, B. GETPrime 2.0: gene- and transcript-specific qPCR primers for 13 species including polymorphisms. *Nucleic Acids Res.* **45**, D56–D60 (2017).
133. Milo, R., Jorgensen, P., Moran, U., Weber, G. & Springer, M. BioNumbers--the database of key numbers in molecular and cell biology. *Nucleic Acids Res.* **38**, D750–3 (2010).
134. Leahy, A., Xiong, J. W., Kuhnert, F. & Stuhlmann, H. Use of developmental marker genes to define temporal and spatial patterns of differentiation during embryoid body formation. *J. Exp. Zool.* **284**, 67–81 (1999).
135. Gohl, D. M. *et al.* Measuring sequencer size bias using REcount: a novel method for highly accurate Illumina sequencing-based quantification. *Genome Biol.* **20**, 85 (2019).
136. Zhao, M., Lee, W.-P., Garrison, E. P. & Marth, G. T. SSW library: an SIMD Smith-Waterman C/C++ library for use in genomic applications. *PLoS One* **8**, e82138 (2013).
