# Supplementary material for: Multiplex profiling of developmental cis-regulatory elements with quantitative single-cell expression reporters
Source: Nat Methods. 2024 May 9;21(6):983–93. doi: 10.1038/s41592-024-02260-3 (PMC11166576; doi:10.1038/s41592-024-02260-3)
Supplement: Supplementary file 2 — Reporting Summary [file 41592_2024_2260_MOESM2_ESM.pdf]

nature portfolio

Corresponding author(s): Shendure

Last updated by author(s): Jan 17, 2024

Reporting Summary

Nature Portfolio wishes to improve the reproducibility of the work that we publish. This form provides structure for consistency and transparency in reporting. For further information on Nature Portfolio policies, see our Editorial Policies and the Editorial Policy Checklist.

Statistics

For all statistical analyses, confirm that the following items are present in the figure legend, table legend, main text, or Methods section.

n/a	Confirmed	
☐	☒	The exact sample size (*n*) for each experimental group/condition, given as a discrete number and unit of measurement
☐	☒	A statement on whether measurements were taken from distinct samples or whether the same sample was measured repeatedly
☐	☒	The statistical test(s) used AND whether they are one- or two-sided<br>*Only common tests should be described solely by name; describe more complex techniques in the Methods section.*
☒	☐	A description of all covariates tested
☒	☐	A description of any assumptions or corrections, such as tests of normality and adjustment for multiple comparisons
☐	☒	A full description of the statistical parameters including central tendency (e.g. means) or other basic estimates (e.g. regression coefficient) AND variation (e.g. standard deviation) or associated estimates of uncertainty (e.g. confidence intervals)
☐	☒	For null hypothesis testing, the test statistic (e.g. *F*, *t*, *r*) with confidence intervals, effect sizes, degrees of freedom and *P* value noted<br>*Give P values as exact values whenever suitable.*
☒	☐	For Bayesian analysis, information on the choice of priors and Markov chain Monte Carlo settings
☒	☐	For hierarchical and complex designs, identification of the appropriate level for tests and full reporting of outcomes
☒	☐	Estimates of effect sizes (e.g. Cohen's *d*, Pearson's *r*), indicating how they were calculated

Our web collection on statistics for biologists contains articles on many of the points above.

Software and code

Policy information about availability of computer code

Data collection	bcl2fastq/2.20, BD FACSDiva™ Software v9.0
Data analysis	pear/0.9.11, seqtk/1.3, R/3.5.1, python/3.6.5, python/3.7.7, samtools/1.10, bedtools/2.29.2, cellranger-6.0.1, bowtie2/2.4.4, Seurat 4.3.1, scrublet 0.2.3. All custom scripts to analyze data have been deposited to GitHub: https://github.com/shendurelab/scQers

For manuscripts utilizing custom algorithms or software that are central to the research but not yet described in published literature, software must be made available to editors and reviewers. We strongly encourage code deposition in a community repository (e.g. GitHub). See the Nature Portfolio guidelines for submitting code & software for further information.

Data

Policy information about availability of data

All manuscripts must include a data availability statement. This statement should provide the following information, where applicable:

- Accession codes, unique identifiers, or web links for publicly available datasets
- A description of any restrictions on data availability
- For clinical datasets or third party data, please ensure that the statement adheres to our policy

Raw sequencing data and processed files generated in this study have been deposited to GEO, with accession number GSE217690. Published data used: transcription factor binding data (Uniprobe: Gata4 accession UP01372, Sox17 accession UP00014, Foxa2 accession UP00073), mouse embryo in vivo scRNA-seq (https://pubmed.ncbi.nlm.nih.gov/30787436/ obtained from R library "MouseGastrulationData") and scATAC-seq (GEO accession GSE205117).

Research involving human participants, their data, or biological material

Policy information about studies with human participants or human data. See also policy information about sex, gender (identity/presentation), and sexual orientation and race, ethnicity and racism.

Reporting on sex and gender	Research not involving human data or participants.
Reporting on race, ethnicity, or other socially relevant groupings	N/A
Population characteristics	N/A
Recruitment	N/A
Ethics oversight	N/A

Note that full information on the approval of the study protocol must also be provided in the manuscript.

Field-specific reporting

Please select the one below that is the best fit for your research. If you are not sure, read the appropriate sections before making your selection.

☒ Life sciences ☐ Behavioural & social sciences ☐ Ecological, evolutionary & environmental sciences

For a reference copy of the document with all sections, see nature.com/documents/nr-reporting-summary-flat.pdf

Life sciences study design

All studies must disclose on these points even when the disclosure is negative.

Sample size	Biological triplicates (n=3) were used, and were sufficient due to high reproducibility in quantification across replicates as detailed in our extended data and supplementary figures.
Data exclusions	No data were excluded from the analyses apart from a single sample/time point from bulk MPRA in mEBs (day 20, replicate 2B1, first round of experiment). This library had been generated from a lower amount of starting RNA (yield from that sample had been lower, suggesting RNA degradation in that sample). Inspection of read counts to basal promoters showed drastically higher apparent activity compared to other samples, suggesting that signal in the RNA originated from trace contaminant genomic DNA, which had a disproportionate weight in that sample due to the low starting RNA quality. This sample was thus excluded from downstream analysis.
Replication	Experiments were reproducible across biological replicates.
Randomization	Not relevant because the samples were not grouped.
Blinding	Not relevant because the sequences tested and samples were not assigned to experimental groups and rather tested all at once as a pool in an unbiased manner.

Reporting for specific materials, systems and methods

We require information from authors about some types of materials, experimental systems and methods used in many studies. Here, indicate whether each material, system or method listed is relevant to your study. If you are not sure if a list item applies to your research, read the appropriate section before selecting a response.

Materials & experimental systems

n/a	Involved in the study	
☒	☐	Antibodies
☐	☒	Eukaryotic cell lines
☒	☐	Palaeontology and archaeology
☒	☐	Animals and other organisms
☒	☐	Clinical data
☒	☐	Dual use research of concern
☒	☐	Plants

Methods

n/a	Involved in the study	
☒	☐	ChIP-seq
☐	☒	Flow cytometry
☒	☐	MRI-based neuroimaging

Eukaryotic cell lines

Policy information about cell lines and Sex and Gender in Research

Cell line source(s)	Male ES cell lines originated from a pure background of BL6 (WD44) obtained as a gift from C. Disteche and C. Ware at the University of Washington. K562 (CCL-243, ATCC), HepG2 (HB-8065, ATCC), HEK293T (CRL-3216, ATCC).
Authentication	Cell lines used were not authenticated.
Mycoplasma contamination	Cell used tested negative for mycoplasma contamination (qPCR with primers pairs 5'-ggt gtg ggt gag tta tta caa art caa tt-3' + 5'-gga gtg agt gga tcc ata aat tgt ga-3', and 5'-ctt cwt cga ctt yca gac cca agg cat-3' + 5'-aca cca tgg gag ytg gta at-3').
Commonly misidentified lines (See ICLAC register)	No commonly misidentified cell lines were used.

Plants

Seed stocks	N/A
Novel plant genotypes	N/A
Authentication	N/A

Flow Cytometry

Plots

Confirm that:

- [x] The axis labels state the marker and fluorochrome used (e.g. CD4-FITC).
- [x] The axis scales are clearly visible. Include numbers along axes only for bottom left plot of group (a 'group' is an analysis of identical markers).
- [x] All plots are contour plots with outliers or pseudocolor plots.
- [x] A numerical value for number of cells or percentage (with statistics) is provided.

Methodology

Sample preparation	Described in Methods.
Instrument	BD FACSAria II
Software	BD FACSDiva v9.0 software was used for acquisition and setting the gates
Cell population abundance	FACS was used to obtain clean single-cell suspensions prior to 10x experiments. Fraction of events considered as bona fide cells (passing gate) was upwards of 55%.
Gating strategy	Supplementary Figure 9 shows gating used. Clear unimodal peak distribution in forward and side scatter intensity (away from the low intensity signal likely coming from debris) was used to mark clean single cells.

- [x] Tick this box to confirm that a figure exemplifying the gating strategy is provided in the Supplementary Information.
